# EDTA‐Functionalized Nanoscale Metal–Organic Framework for Onco‐Cardiology via Radiochemotherapy Synergy and Spatiotemporally Matched Iron Chelation

**DOI:** 10.1002/advs.202521451

**Published:** 2026-02-08

**Authors:** Daojing Yuan, Liyang Tian, Luwen Zhuang, Yongyu Liang, Lingfei Chen, Xuetao Wang, Yanli Li, Xiyong Yu, Teng Gong

**Affiliations:** ^1^ Guangzhou Municipal and Guangdong Provincial Key Laboratory of Molecular Target & Clinical Pharmacology the NMPA and State Key Laboratory of Respiratory Disease School of Pharmaceutical Sciences Guangzhou Medical University Guangzhou China; ^2^ Center for Water Resources and Environment and Guangdong Key Laboratory of Marine Civil Engineering School of Civil Engineering Sun Yat‐sen University Guangzhou China; ^3^ The Second Affiliated Hospital of Guangzhou University of Chinese Medicine Guangzhou China

**Keywords:** cardiotoxicity, EDTA, ferroptosis, metal–organic framework, oncotherapy

## Abstract

Doxorubicin (DOX)‐induced cardiotoxicity (DIC) has become a major obstacle for clinical application. While ferroptosis represents a critical therapeutic target for DIC, current intervention strategies are limited by spatiotemporal mismatches between DOX accumulation and iron chelation. Here, we engineered an EDTA‐functionalized Hf‐based metal–organic framework for DOX delivery. This nanoplatform (**DME**) simultaneously mediates tumor radiochemotherapy synergy and inhibits ferroptosis via real‐time iron chelation in cardiac tissue. The EDTA modification not only enhances drug penetration to kill deep‐seated tumors, but also endows **DME** superior iron‐scavenging ability, which effectively suppresses mitochondrial‐dependent ferroptosis in cardiomyocytes. Using primary cultured neonatal mice cardiomyocytes and a chronic DIC murine model, we demonstrated **DME**’s significant cardioprotection and elucidated its mechanistic basis. Thus, our work establishes a bifunctional nanoplatform that unifies oncotherapy and cardioprotection, offering a spatiotemporally matched iron‐chelation strategy for safe and effective clinical use of DOX.

## Graphical abstract

Doxorubicin (DOX)‐induced cardiotoxicity is a major limitation to its broader clinical application, while cardioprotective interventions often risk reducing the anticancer efficacy of DOX. To address these challenges, this work constructs an EDTA‐functionalized metal–organic framework that enhances tumor therapy and provides efficient cardioprotection through radiochemotherapy synergy and spatiotemporally matched iron chelation, respectively.

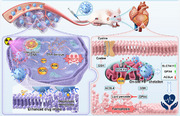

## 1 Introduction

Onco‐cardiology primarily explores shared risk factors and therapeutic strategies for cancer and cardiovascular disease, with a focus on cardiovascular complications caused by anti‐cancer treatments, comorbid conditions, and cardiac tumors [1]. Advances in therapy modalities have significantly prolonged cancer survival, but this progress has also been accompanied by a rising incidence and mortality of treatment‐induced cardiotoxicity. Chemotherapeutics can directly impair cardiac structure and function or indirectly trigger myocardial remodeling through hypertension. Among these, anthracyclines such as doxorubicin (DOX) are the leading cause of cardiotoxicity, primarily through mechanisms involving reactive oxygen species (ROS) generation and ferroptosis [2, 3, 4]. Dexrazoxane remains the only first‐line clinical drug approved to mitigate DOX‐induced cardiotoxicity (DIC), but its application is constrained by myelosuppression and potential interference with anticancer efficacy [5]. In recent years, nanomedicine has opened new avenues for alleviating DIC, leveraging multifunctional integration to create innovative therapeutic platforms [6, 7, 8, 9, 10]. Strategies include co‐delivery of DOX with anti‐inflammatory agents or iron chelators, as well as nanozymes designed to efficiently scavenge ROS. However, because anti‐tumor and DIC alleviation involve different mechanisms, developing nanomedicines that can both enhance oncotherapy and cardioprotection remains a major challenge.

Currently, two major challenges exist in the concurrent treatment of tumors and DIC: (1) Complex interactions between detoxifying agents and DOX may compromise the intended therapeutic efficacy, reducing both cardioprotection and anti‐tumor potency. To prevent this antagonistic effect, a combination of regimen that integrate chemotherapy with other clinical oncology approaches is essential. (2) Spatiotemporal mismatch between DOX cardiac accumulation and detoxifier delivery severely limits cardiotoxicity management. Because of its high affinity for cardiolipin, abundantly expressed in the inner mitochondrial membrane of cardiomyocytes, DOX preferentially accumulates in cardiac mitochondria at concentrations up to 100‐fold higher than plasma levels [11, 12, 13]. This excessive accumulation induces mitochondrial damage, triggering both mitochondrion‐dependent ferroptosis and ROS‐mediated apoptosis, ultimately driving cardiotoxicity [11, 14]. Critically, once DOX causes cardiac injury, particularly the long‐term toxicity resulting from chronic accumulation, the damage is typically progressive and irreversible. Therefore, detoxifying agents must act immediately upon DOX cardiac accumulation to achieve meaningful cardioprotection. An ideal therapeutic platform should not only enhance anti‐tumor efficacy at the tumor site but also mount a real‐time response to off‐target DOX deposition in cardiac tissue.

**SCHEME 1:**
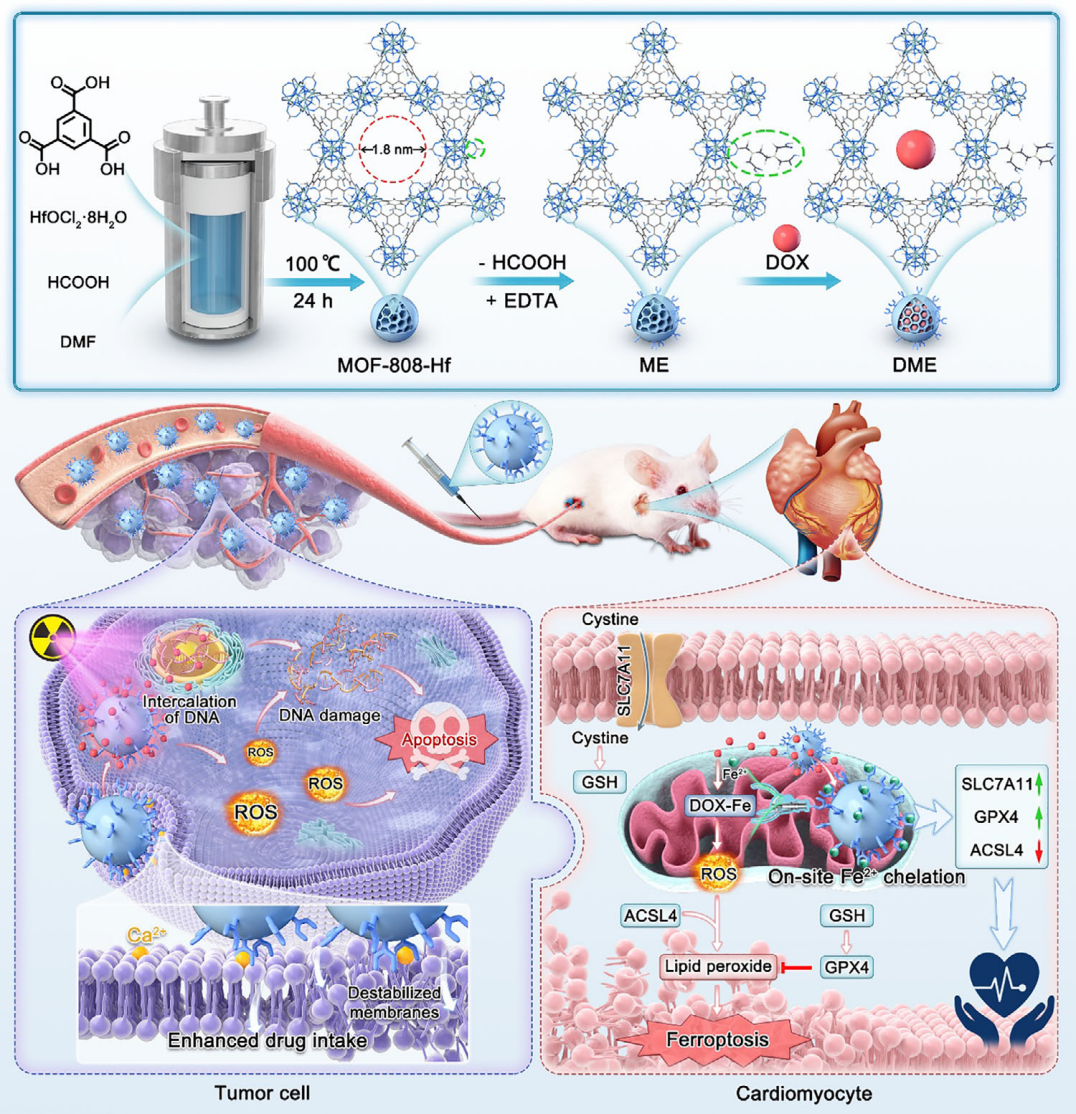
Schematic illustration for the synthesis of EDTA‐functionalized MOFs and their role in oncotherapy and cardioprotection. Incorporating EDTA and Hf_6_ clusters into nanoscale MOFs not only enhances DOX penetration into tumors but also synergistically improves radiosensitization, effectively combating resistant tumors. For cardioprotection, **DME** reduces Fe^2+^ ions accumulation in cardiomyocytes, thereby mitigating mitochondrial‐dependent cardiac injury.

Metal–organic frameworks (MOFs) are infinitely extended porous coordination networks composed of metal ions or clusters connected by organic ligands. Their structural diversity, ordered porosity, and tunable properties have enabled broad applications in adsorption, storage, separation, energy conversion, catalysis, sensing, and biomedicine [15]. Among these, hafnium‐based MOFs, which employ Hf‐oxo clusters as metal nodes, show exceptional promise in radiosensitization and radio‐immunotherapy owing to the enhanced X‐ray energy deposition of high‐Z elements [16, 17, 18, 19]. Several Hf‐MOF‐derived nanomedicines have already progressed to clinical trials. Building on this foundation, we anchored EDTA onto the metal nodes of Hf‐MOFs via coordination substitution. The resulting post‐modified MOFs were loaded with DOX (termed **DME**) for synergistic tumor radiochemotherapy while mitigating DIC (Scheme 1). For oncotherapy, **DME** possesses enhanced chemotherapeutic synergy and retains robust efficacy even against drug‐resistant tumors. Meanwhile, EDTA modification enables **DME** to capture iron ions at sites of DOX leakage, markedly suppressing DOX‐induced ferroptosis in cardiomyocytes. The abundant coordination sites of EDTA occupy the catalytic centers of Fe^2+^‐mediated Fenton reactions, concurrently inhibiting intracellular ROS generation. Using both primary neonatal mice cardiomyocyte models and chronic DIC mouse models, we validated **DME**’s superior cardiotoxicity mitigation. This so‐called “spatiotemporally matched” iron‐chelating strategy represents a promising design paradigm for precision DIC therapeutics.

## 2 Results

### 2.1 Design and Characterization of DME Nanoparticles

**FIGURE 1:**
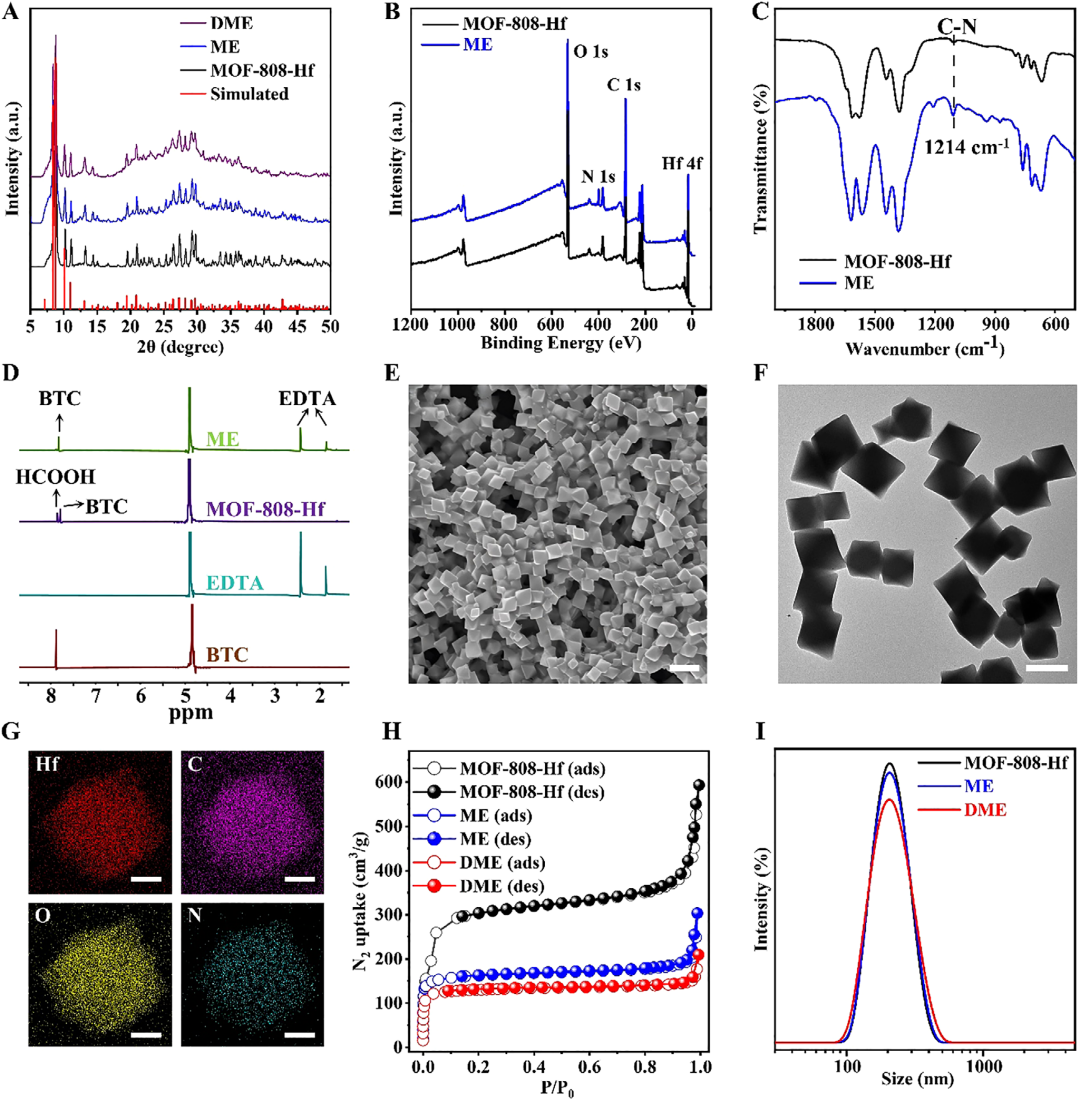
Characterization of DME. PXRD patterns (A), XPS data (B), and IR spectra (C) of different as‐synthesized nanoparticles. (D) ^1^H NMR spectra of digested **ME**, digested MOF‐808‐Hf, trimesic acid (BTC), and EDTA. (E–G) SEM, TEM, and element mapping images of **DME**. Scale bars: 500 nm (H), 200 nm (F), 50 nm (G). (H) N_2_ adsorption/desorption isotherms of MOF‐808‐Hf, **ME**, and **DME**. (I) Particle size distributions of different nanoparticles.

The design and preparation of toxicity‐reducing and efficacy‐enhancing nanoformulations rely on the functionalization of Hf‐based MOF‐808 (termed MOF‐808‐Hf). The formate groups on the Hf_6_ clusters can be readily replaced with other carboxylic acid ligands, while the nanoscale, cage‐like channels of the MOF facilitate DOX encapsulation [20, 21]. Uniform octahedral MOF‐808‐Hf nanoparticles, approximately 200 nm in size, were synthesized via a classic solvothermal method (Figure S1). PXRD spectra confirmed that the as‐synthesized nanoparticles possess a well‐defined crystalline structure (Figure 1A). Subsequently, EDTA was anchored onto the metal clusters of the MOF through a coordination substitution reaction in aqueous media (termed **ME**). The successful EDTA functionalization was confirmed through multiple spectroscopic techniques. In comparison with MOF‐808‐Hf, the IR spectrum of **ME** displayed a characteristic peak at 1214 cm^−1^, attributed to the C─N stretching vibration of EDTA, which is consistent with the N 1s peak observed in the XPS spectra (Figure 1B,C) [22]. Furthermore, high‐resolution O 1s peak revealed a new peak at 532.8 eV in **ME**, corresponding to uncoordinated carboxylate groups from the anchored EDTA (Figure S2). More direct evidence was provided by the ^1^H NMR spectra of the digested materials. As shown in Figure 1D, the formate peaks were completely absent, while distinct methylene proton signals at 1.8 and 2.4 ppm appeared in the **ME** sample, clearly indicating that EDTA functionalization was achieved via substitution of the formate ligands on the MOF‐808‐Hf [23]. Elemental analysis determined the EDTA loading amount to be 30.5 wt.% in **ME**.

DOX was loaded into the **ME** pores via physical adsorption, referred to as **DME**. TEM and SEM images showed no significant changes in material morphology or particle size compared to **ME** (Figure 1E,F). Element mapping further demonstrated the uniform distribution of Hf, C, O, and N elements throughout the nanoparticle (Figure 1G). Following drug loading, **DME** exhibited a red coloration and inherited the photoluminescent properties of DOX (Figure S3). Brunauer–Emmett–Teller (BET) analysis revealed that all three MOF nanoparticles showed type I N_2_ adsorption isotherms at 77 K, indicating their microporous structural characteristics. The BET surface areas were determined to be 937.3 m^2^/g for MOF‐808‐Hf, 502.5 m^2^/g for **ME**, and 391.8 m^2^/g for **DME** (Figure 1H). The sequential introduction of EDTA and DOX into the framework leads to a corresponding decrease in the measured surface area. What's more, a decrease in the average pore size from 1.72 to 0.61 nm was observed, implying that the pores were progressively occupied or blocked upon cargo loading (Figure S4). The drug loading rate was determined to be 20 wt.% using a fluorescent‐based method. The molar ratio of EDTA to DOX is 5:2 in **DME**. This substantial drug loading capacity is attributed to the large surface area of **ME** and the interactions between DOX and **ME**, such as π‐π stacking and hydrogen bonding. Dynamic light scattering (DLS) analysis showed that the average hydrodynamic diameters of the three nanoparticles (∼206.7 nm) remained largely unchanged (Figure 1I).

**FIGURE 2:**
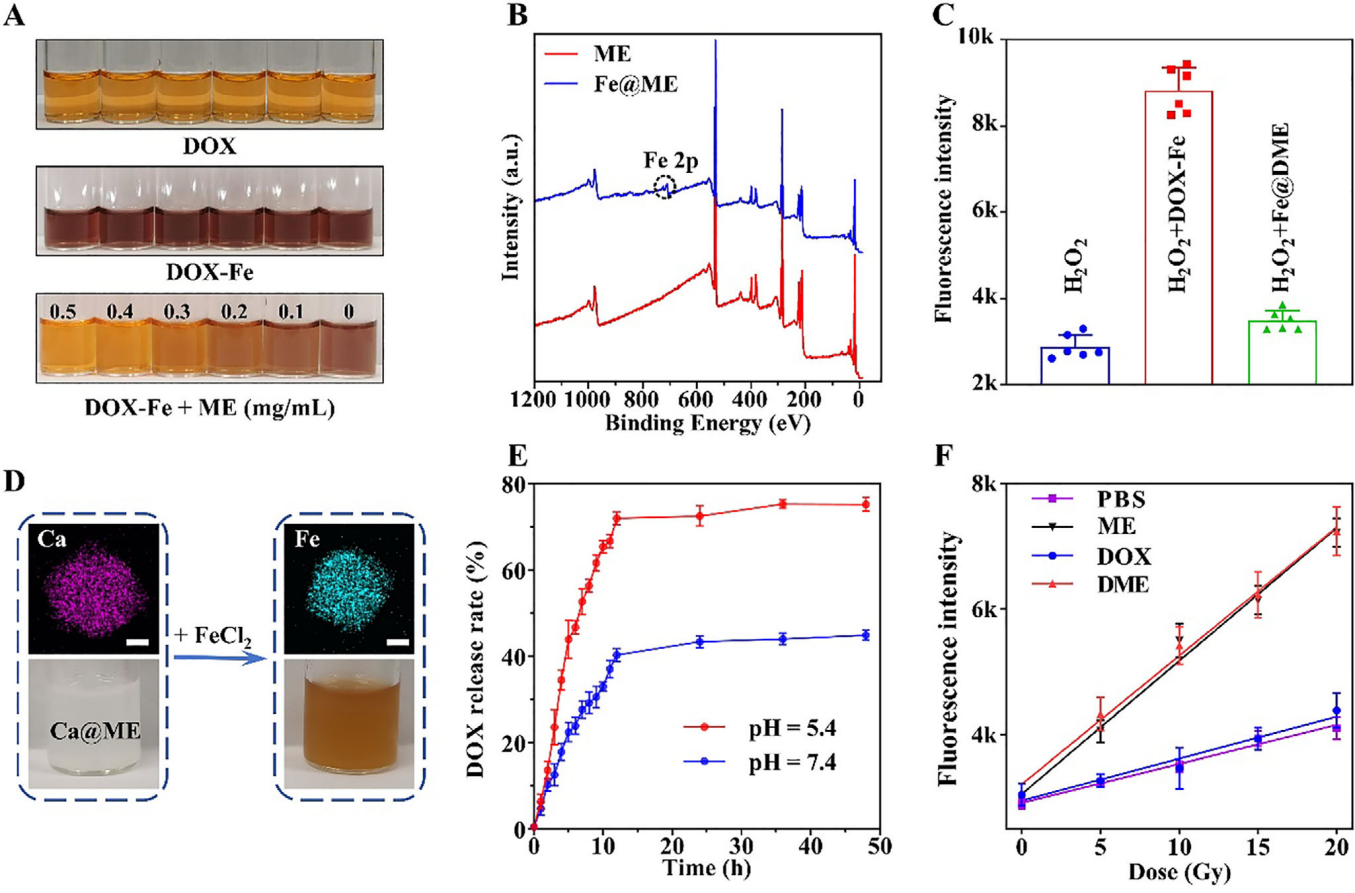
Validation of DME function in aqueous solution. (A) Photographs showing competitively Fe^2+^ deprivation from DOX‐Fe complexes by **ME** at various concentrations. (B) XPS spectra of **ME** before and after Fe^2+^ chelation. (C) Fluorescence intensity of 7‐hydroxycoumarin (λ_em_ = 456 nm) under different reaction conditions (pH = 7.4), demonstrating inhibition of the Fe^2+^‐mediated Fenton reaction by chelation. (D) Photographs and element mapping of **Ca@ME** before and after FeCl_2_ treatment. Scale bar: 60 nm. (E) DOX release profile from **DME** under pH 7.4 and 5.4. (F) Radiation dose‐dependent changes in fluorescence intensity (λ_em_ = 456 nm) monitored with 7‐hydroxycoumarin in different solutions.

Fe^2+^ ions and DOX‐Fe complexes are the major contributors to lipid peroxidation and ferroptosis in DIC [24, 25]. **DME** alleviates this cardiotoxicity by chelating Fe^2+^ ions via EDTA, preventing the formation of DOX‐Fe complexes. To explore this mechanism, we systematically investigated the competitive coordination between **ME** and DOX‐Fe complexes. Aqueous DOX solution appeared orange‐yellow, exhibiting characteristic UV–vis absorption peaks at 233, 253, and 290 nm (Figure 2A; Figure S5). Upon addition of Fe^2+^ ions, DOX‐Fe complex formation caused a color change to dark brown along with increased absorption intensity. Subsequent introduction of **ME** nanoparticles with different concentrations induced a concentration‐dependent color reversal and a concomitant decrease in absorption intensity, indicating effective Fe^2+^ sequestration from DOX‐Fe complexes by **ME**. XPS analysis confirmed the presence of Fe 2p signals in Fe‐absorbed material (termed **Fe@ME**), further validating Fe^2+^ chelation (Figure 2B) [23]. Kinetic studies demonstrate that this competitive coordination follows pseudo‐first‐order kinetics with a rate constant of *k* = 1.96 h^−1^ (Figure S6). Considering the role of Fe^2+^ in cellular ROS generation, we evaluated the Fenton catalytic activity of Fe^2+^‐chelated **DME** (**Fe@DME**) and DOX‐Fe in aqueous solution (pH 7.4) [26, 27]. Fluorescence monitoring with a coumarin‐based indicator showed that **Fe@DME** failed to catalyze H_2_O_2_ conversion to hydroxyl radicals (•OH), likely because polydentate EDTA blocked the Fe^2+^ active sites and inhibited the Fenton process (Figure 2C). These results further support the cardioprotective potential of **DME** via Fe^2+^ ions sequestration.

The potential interference of metal ions (e.g., Ca^2+^, Mg^2+^, Zn^2+^, and Cu^2+^) with **DME** in the circulatory system should be evaluated. Because Ca^2+^ and Mg^2+^ exist at millimolar concentrations in blood, significantly higher than the micromolar levels of Zn^2+^, Cu^2+^, and Mn^2+^, we prioritized evaluating their impact on **DME**. Grounded in coordination chemistry, the stability constants of EDTA‐metal complexes vary considerably, with the stronger complex displacing the weaker one in a competitive manner. The stability constants (log *K*) for the complexes of EDTA with Mg^2+^, Ca^2+^, and Fe^2+^ under neutral conditions are 8.7, 10.7, and 14.3, respectively [28]. Because Ca^2+^ has a stability constant closer to that of Fe^2+^ than Mg^2+^, we evaluated the iron‐removal efficacy of **ME** after pre‐chelating Ca^2+^. In particular, we dispersed Ca^2+^‐chelated **ME** (**Ca@ME**) nanoparticles into an equivalent Fe^2+^ solution. After 1 h reaction, elemental mapping analysis revealed that most Ca^2+^ had been replaced by Fe^2+^, which can be attributed to the competitive coordination effect that favors the formation of more stable chelates (Figure 2D; Figure S7). These findings suggest that the **ME**‐mediated Fe^2+^ chelation is not significantly affected by the physiological concentrations of Ca^2+^ in the blood.

The drug release behavior was evaluated under pH 7.4 and 5.4. As shown in Figure 2E, **DME** exhibited time‐dependent and sustained DOX release. While drug release profiles were comparable within the first two hours, a marked divergence occurred by 3 h, with release at pH 5.4 reaching ∼32% compared to ∼18% at pH 7.4. The cumulative drug release reached ∼75% under acidic conditions by 12 h, compared to only ∼40% under neutral conditions. The acidic condition accelerated dug release, probably because the DOX protonation disrupted these host‐guest interactions within MOFs [29]. Beyond its sustained drug‐release capability, we also examined the X‐ray energy deposition properties of **DME**. The yield of •OH serves as a critical indicator for assessing radiosensitization efficacy. Using the coumarin‐trapping spectrofluorimetry, **DME**‐containing aqueous solutions produced high •OH levels under X‐ray irradiation, attributable to the high‐Z element Hf efficiently absorbing high‐energy radiation (Figure 2F) [19]. To assess the stability of these nanomedicines in biological media, we measured the DLS and Zeta potential of different as‐synthesized nanoparticles in saline, PBS, and DMEM after 7 days storage. As shown in Figure S8 and Table S1, the DLS and Zeta potential of different nanoparticles were almost unchanged in different media, indicating their remarkable stability. The high stability of the nanoparticles originates from the robust MOF‐808 platform, which arises from the strong coordination bonds between high‐valence Hf^4+^ ions (hard acid) and carboxylate ligands (hard base) [30, 31].

### 2.2 Evaluating the Efficacy of DME‐Mediated Radiochemotherapy Synergy

**FIGURE 3:**
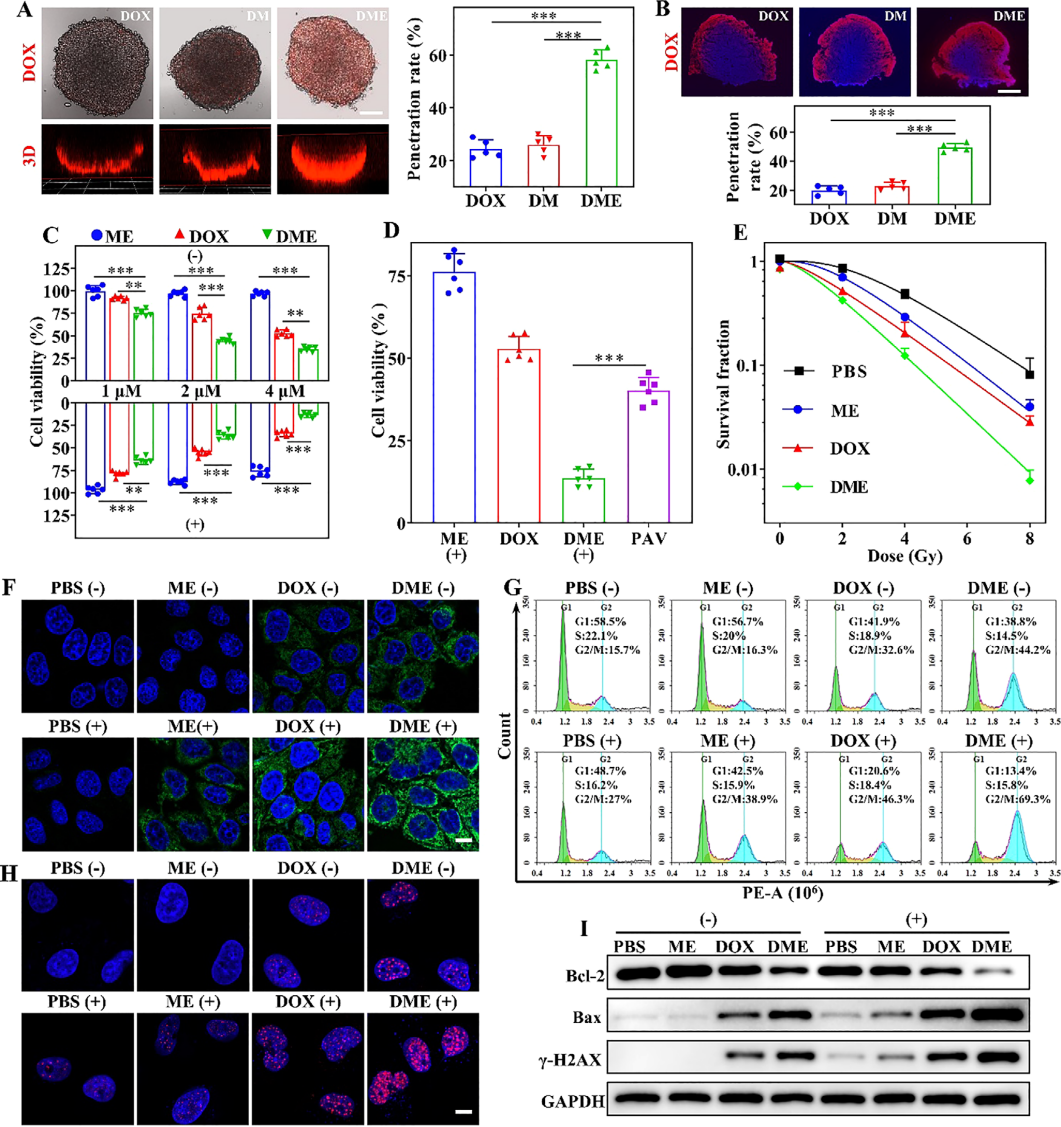
Evaluation of the anti‐tumor efficacy of DME in vitro. (A) Penetration of DOX, DOX‐loaded MOF‐808‐Hf (**DM**), and **DME** in MCF‐7 3D cell spheroids after 12 h of co‐incubation. Adjacent panel shows quantification of drug permeability based on DOX fluorescence intensity. Scale bar: 80 µm. (B) Cryosection analysis 12 h after peritumoral injection of different drugs to evaluate penetration into solid tumors. Scale bar: 1.5 mm. (C) Cell viability after treatment with **ME**, DOX, and **DME** at varying concentrations with or without X‐ray irradiation. (D) Synergistic therapeutic effect of **DME**‐mediated chemoradiotherapy. The projected additive value (PVA) was calculated by multiplying the cell viability of the **ME** + X‐ray group by that of the DOX group. (E) Radiation dose‐dependent cell survival curves determined by colony formation assay. (F) Detection of intracellular •OH. Scale bar: 10 µm. (G) Flow cytometry for the cell cycle distribution in different treatment groups. (H) *γ*‐H2AX immunofluorescence analysis to assess DNA double‐strand breaks following various treatments. Scale bar: 10 µm. (I) Western blot analysis of apoptosis‐related proteins and *γ*‐H2AX across treatment groups. + and − indicate samples treated with or without 4 Gy X‐ray irradiation, respectively. ^**^p <0.01, ^***^p <0.001.

Studies have reported that EDTA can enhance cellular uptake and tumor penetration through fluidizing the cell membrane or Ca^2+^ chelation, improving drug delivery efficiency [32]. Building on this, we first investigated the cellular uptake of DOX‐loaded MOF‐808‐Hf (termed **DM**) and **DME**. Compared with the **DM** group, **DME** showed significantly higher cellular internalization, as confirmed by DOX fluorescence quantitation (Figure S9). In 3D cell spheroids that mimic solid tumors in vitro, EDTA also enhanced DOX delivery (Figure 3A). Furthermore, DOX, **DM**, and **DME** were administered via peri‐tumoral injection, and tumor sections were analyzed 12 h post‐injection to assess DOX penetration depth in vivo. Consistent with in vitro results, the **DME** group exhibited markedly improved drug penetration into solid tumors (Figure 3B). Overall, data from both in vitro and tumor‐bearing mouse models demonstrate that EDTA modification facilitates deeper tumor penetration.

The effects of **DME** on cellular viability and proliferative capacity were evaluated based on CCK‐8 and colony formation assays with/without X‐ray treatment (4 Gy). MCF‐7 and multidrug‐resistant MCF‐7/ADR tumor cell lines served as the evaluation platform. The CCK‐8 assay revealed no significant cytotoxicity in the **ME** group, suggesting favorable biocompatibility of this carrier system (Figure 3C). Compared to free DOX, **DME** demonstrated significantly enhanced cytotoxicity, owing to more DOX internalization. Moreover, combining **DME** with radiotherapy further amplified cytotoxic effects across all groups, attributable to heavy metal‐enhanced X‐ray deposition and chemoradiotherapy synergy (Figure 3C; Figure S10A) [33]. This synergistic effect was also confirmed by comparing the observed cell survival rates to predicted additive values, with **DME**‐mediated chemoradiotherapy showing lower survival than expected (Figure 3D; Figure S10B). Since radiotherapy primarily impairs tumor cell proliferation, clonogenic assays were conducted to evaluate the radiosensitization potential of these nanodrugs. The multitarget single‐hit model was employed to calculate sensitization enhancement ratio (SER) for each group [34]. Results were consistent with the CCK‐8 assays: **DME** group exhibited the highest radiosensitization, further highlighting the advantages of combined chemoradiotherapy (Figure 3E; Figures S11, S12, and Table S2).

Encouraged by the promising therapeutic effects observed in vitro, we conducted preliminary validation of the **DME**‐mediated chemoradiotherapy synergistic mechanism in tumor cells. Both DOX and high‐energy radiation induce substantial intracellular ROS during cell death. Accordingly, DCFH‐DA and HPF were used as indicators to measure intracellular ROS levels (Figure 3F; Figures S13 and S14). DCFH‐DA reflects overall ROS levels, whereas HPF is primarily used for the quantitative detection of •OH. Before X‐ray irradiation, the **DME**‐treated group exhibited stronger intracellular fluorescence than the DOX group, consistent with the cellular uptake and CCK‐8 results. Following X‐ray exposure, the ROS levels, including •OH, increased markedly in both the **ME** and **DME** groups, indicating ROS accumulation is a major cause of cell damage. In addition, DOX intercalates into DNA base pairs to inhibit replication and transcription, thereby inducing cell cycle arrest [35]. Cell cycle analysis showed that **DME** specifically arrested the tumor cells at the radiosensitive G2/M phase (44.2% for **DME** vs. 32.6% for DOX), representing a key mechanism underlying **DME**‐mediated chemoradiotherapy synergy (Figure 3G).

DNA serves as the primary target for both DOX and radiotherapy. We assessed DNA damage across different treatment groups using the comet assay. The longest comet tails observed in the **DME** + X‐ray group implied the most severe DNA damage, which was concordant with the observed cell cycle arrest (Figure S15). DNA double‐strand breaks (DSBs) are considered the most deleterious form of DNA damage, typically heralding irreversible cellular death. *γ*‐H2AX foci are a validated biomarker for quantifying DSBs. In agreement with the comet assay results, the **DME** + X‐ray group showed the highest number of *γ*‐H2AX foci, with 3.7 and 1.9 times increase compared to the **ME** + X‐ray and DOX + X‐ray groups, respectively (Figure 3H; Figure S16). Elevated *γ*‐H2AX levels in the **DME** + X‐ray group were further confirmed by Western blot (WB) analysis (Figure 3I). EdU (5‐ethynyl‐2’‐deoxyuridine), a thymidine analog incorporated into newly synthesized DNA, serves as a precise indicator of cell proliferation and DNA replication. EdU staining revealed that **DME** alone significantly suppressed DNA replication and proliferation due to efficient DOX delivery. Upon X‐ray treatment, MCF‐7 cell proliferation in the **DME** group was almost inhibited (Figure S17). These results correlate with the cell cycle and DNA damage data, confirming the synergistic efficacy of **DME**‐mediated chemoradiotherapy. DNA damage and ROS accumulation trigger apoptosis. Apoptosis was assessed across treatment groups based on Annexin V/RedNucleus II flow cytometry and analysis of apoptosis‐related proteins. The **DME** + X‐ray group displayed the highest apoptosis rate (∼84.2%) (Figure S18) and pronounced changes in Bax and Bcl‐2 expression (Figure 3I; Figure S19). Collectively, the strong in vitro antitumor efficacy of **DME** establishes a solid foundation for subsequent in vivo therapeutic studies.

To enhance tumor‐targeting capability of **ME** and **DME** in vivo, tumor cell membranes were coated onto the nanoparticles, yielding a formulation termed **MEC** and **DMEC**. After coating, the zeta potential of **DMEC** shifted from 21.4 to −18.2 mV, accompanied by only minor changes in morphology and size (Figure S20). Compared with **DME**, the additional P and S elements observed in elemental mapping originated from the cell membranes (Figure S21). Protein gel electrophoresis was performed to analyze the membrane proteins of **DMEC**, isolated cell membranes, and tumor cells. The protein band profiles of **DMEC** closely matched those of pure cell membranes, confirming successful coating (Figure S22). To evaluate biosafety, **DMEC** (5 mg/kg based on DOX) was administered to Balb/c mice via tail vein injection. Compared with the PBS group, no significant differences were observed in body weight, blood biochemical parameters, or histological sections of major organs after 3 and 30 days, demonstrating favorable biosafety (Figure S23). For biodistribution studies, DOX was replaced with indocyanine green (ICG) as a tracer, and the time‐dependent tissue distribution of nanoparticles before and after membrane coating was monitored using a small animal fluorescence imaging system. As seen in Figures S24 and S25, tumor cell membrane‐camouflaged nanoparticles exhibited significant accumulation in tumor tissue, attributed to homologous targeting [36]. Based on Hf^4+^ ions concentration measurements, the blood half‐life of **DMEC** was calculated to be approximately 52.8 min (Figure S26). At 12 h post‐administration, **DMEC** reached peak tumor accumulation, consistent with the fluorescence imaging results (Figures S24 and S25).

**FIGURE 4:**
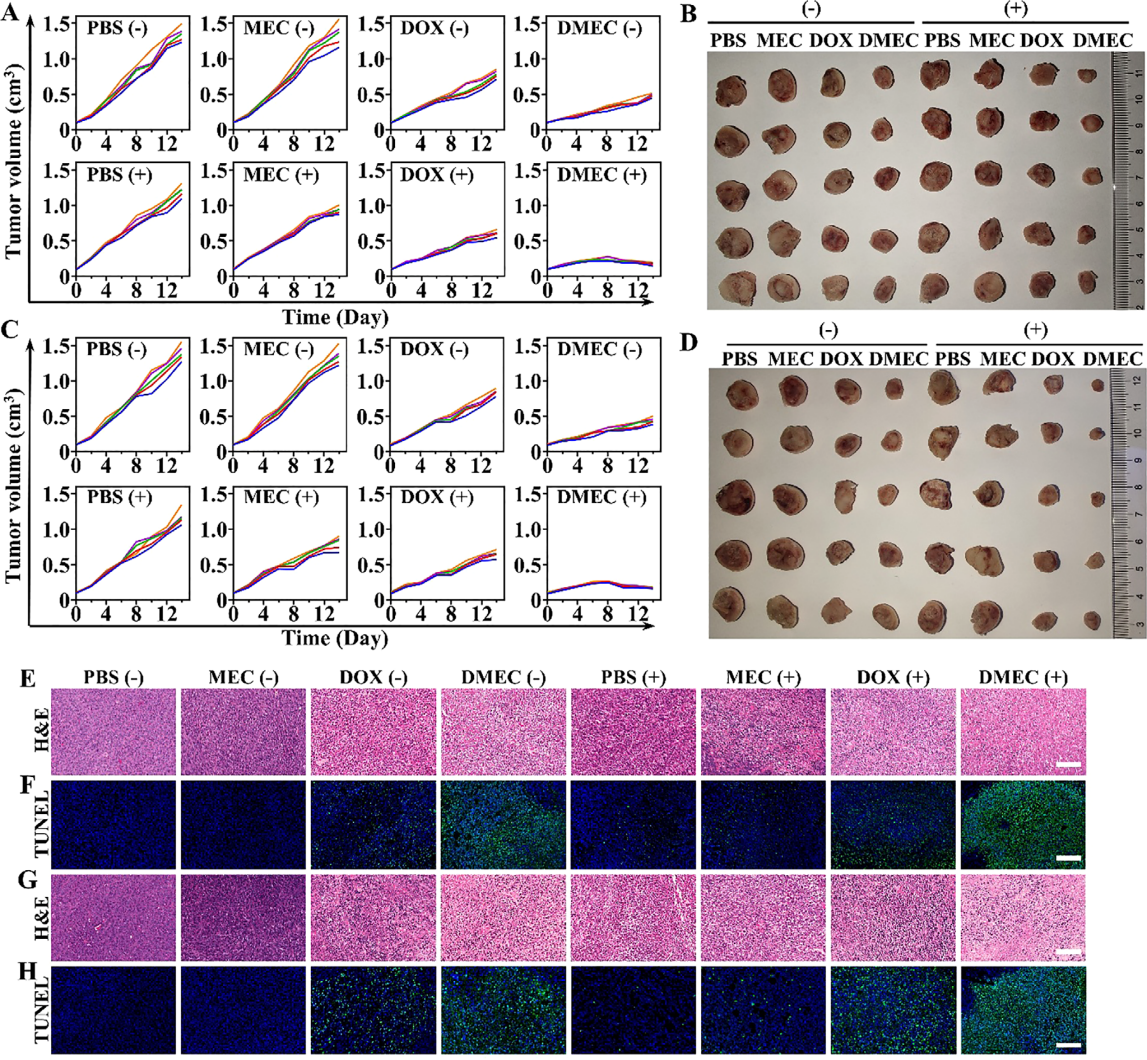
DMEC‐mediated enhancement of chemoradiotherapy efficacy in MCF‐7 and MCF‐7/ADR subcutaneous tumor models. (A) Tumor volume changes in MCF‐7 tumors during treatment. (B) Representative images of MCF‐7 tumors after 14 days of treatment. (C) Tumor volume changes in MCF‐7/ADR tumors during treatment. (D) Representative images of MCF‐7/ADR tumors after 14 days of treatment. (E,F) Representative H&E and TUNEL staining images of MCF‐7 tumors following the second radiotherapy (10th day of treatment). Scale bar: 100 µm. (G,H) Representative H&E and TUNEL staining images of MCF‐7/ADR tumors following the second radiotherapy (10th day of treatment). Scale bar: 100 µm. + and − indicate tumors treated with or without cumulative 8 Gy X‐ray irradiation, respectively.

Human breast cancer (MCF‐7) and chemotherapy‐resistant MCF‐7/ADR xenografts models were established to evaluate the therapeutic efficacy of nanodrug‐mediated chemoradiotherapy. When tumors reached ∼100 mm^3^, mice were randomly assigned to eight groups (*n* = 6): PBS, **MEC**, DOX, **DMEC**, PBS + X‐ray, **MEC** + X‐ray, DOX + X‐ray, **DMEC** + X‐ray. During the 14‐day treatment regimen, intravenous injections were administered via the tail vein on day 0 (treatment initiation) and day 7, with doses of 2 mg/kg for MCF‐7 tumors and 5 mg/kg for MCF‐7/ADR tumors based on DOX content per injection. Tumors subsequently received 4 Gy X‐ray irradiation after 12 h of administration based on biodistribution studies (Figures S24 and S25). Tumor volume monitoring and post‐treatment tumor weight analysis demonstrated that the **DMEC** + X‐ray group achieved the most pronounced tumor suppression (85.6% for MCF‐7 and 82.1% for MCF‐7/ADR based on tumor mass), consistent with the effectiveness observed in vitro (Figure 4A–D; Figures S27 and S28). All mice exhibited steady body weight gain, indicating minimal treatment‐related toxicity (Figures S29 and S30). Histopathological analysis of post‐treatment tumor sections revealed increased cellular damage, apoptosis, and DNA damage marker *γ*‐H2AX, consistent with the observed therapeutic outcomes, reaffirming the efficacy of **DMEC**‐mediated chemoradiotherapy synergy in enhancing oncotherapy (Figure 4E–H; Figures S31 and S32).

### 2.3 Evaluation of DME in Alleviating DOX‐Induced Cardiotoxicity

**FIGURE 5:**
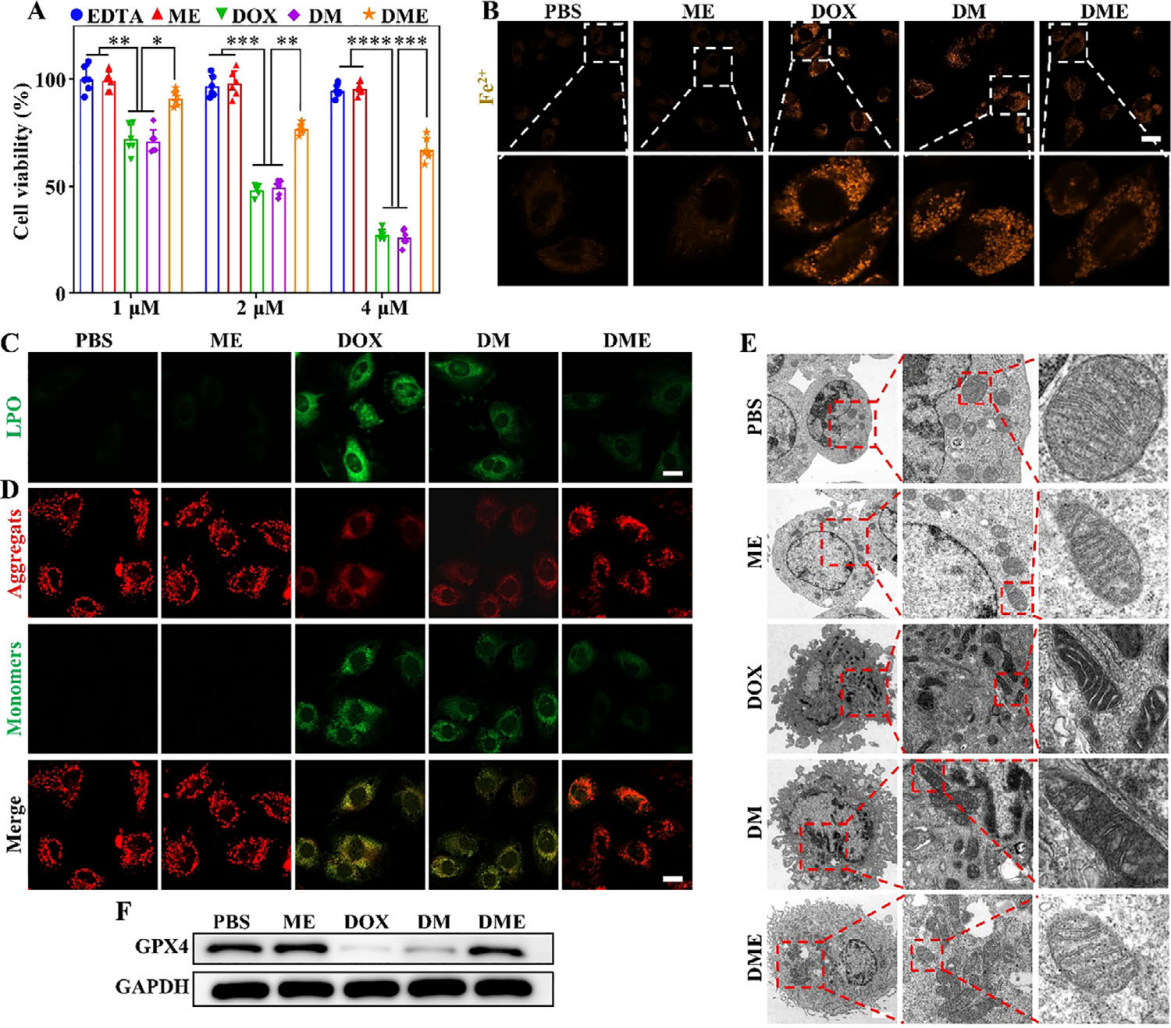
Assessment of DME‐mediated protection against DIC in neonatal mice cardiomyocytes. (A) CCK‐8 assay of varying concentrations of DOX, **ME**, **DM**, and **DME** on cardiomyocytes. (B) Detection of intracellular Fe^2+^ levels after different treatments. Scale bar: 20 µm. (C) Assessment of lipid peroxidation in cardiomyocytes after different treatments. Scale bar: 20 µm. (D) Confocal laser scanning microscopy images of JC‐1 staining in cardiomyocytes. An increased JC‐1 monomer signal is indicative of a reduction in mitochondrial membrane potential. Scale bar: 20 µm. (E) Bio‐TEM images of cardiomyocytes subjected to different treatments. Scale bar: 2 µm. (F) GPX4 expression levels in cardiomyocytes after different treatments. ^**^p <0.01, ^***^p <0.001, ^****^p <0.0001.

To date, dexrazoxane is the only FDA‐approved drug for alleviating DIC. Its protective effect is attributed to the reduction of cellular damage and lipid peroxidation through iron chelation. Recent studies have further highlighted that mitochondrial‐dependent ferroptosis is a key mechanism underlying DIC [24, 25]. In this context, DME, with potent iron‐chelating properties, holds promise for mitigating cardiac damage. An in vitro evaluation model of DIC was established using primary cardiomyocytes isolated from neonatal mice. At equivalent DOX concentrations, both DOX and DM induced comparable cytotoxicity, whereas DME exhibited significantly lower toxicity, especially at higher doses (Figure 5A). FerroOrange is an intracellular Fe^2+^ detection probe that forms an irreversibly orange‐fluorescent complex through specific binding to Fe^2+^. Following co‐incubation with FerroOrange, DOX treatment led to substantial Fe^2+^ release in cardiomyocytes (Figure 5B). In contrast, DME effectively attenuated this Fe^2+^ surge via iron chelation, preventing intracellular Fe^2+^ accumulation. Moreover, we also evaluated the levels of Fe^2+^ in cardiomyocytes treated with DOX, DM, DME, and Ca^2+^‐chelated DME (Ca@DME). As shown in Figure S33, Ca@DME group also showed excellent inhibition effect of free Fe^2+^ generation. These results suggested that Ca@DME still possessing good chelating ability for Fe^2+^, because EDTA has a higher chelating effect for Fe^2+^ than for Ca^2+^ ions. DOX can coordinate with Fe^2+^ to form DOX‐Fe. This complex induces extensive lipid peroxidation in cardiomyocytes, which is an established hallmark of the ferroptosis cascade [24]. To assess lipid peroxidation, we employed C11‐BODIPY as an indicator. Confocal images demonstrated that DME markedly suppressed intracellular lipid peroxide accumulation compared with DOX and DM groups (Figure 5C). A similar trend was observed in DCFH‐DA‐based intracellular ROS assays (Figure S34). These findings indicate that DME confers cardioprotection against DOX‐driven ferroptosis via high‐affinity iron sequestration.

Mitochondrial dysfunction represents an important pathological feature of DIC, including mitochondria‐driven ferroptosis. Using JC‐1 ratiometric imaging, we directly visualized DOX‐induced mitochondrial depolarization and observed its subsequent recovery after **DME** treatment (Figure 5D; Figure S35). Complementary bio‐TEM analysis revealed that DOX increased mitochondrial membrane density, reduced mitochondrial volume, and caused cristae fragmentation or loss in cardiomyocytes, all of which were effectively mitigated by **DME** treatment (Figure 5E). A comprehensive evaluation of lipid oxidative and mitochondrial pathology further confirmed **DME**’s anti‐ferroptotic effects in cardiomyocytes. Mechanistically, **DME** strongly upregulated the master regulator of ferroptosis defense glutathione peroxidase 4 (GPX4), facilitating enzymatic detoxification of cytotoxic lipid peroxides and preventing ferroptosis (Figure 5F; Figure S36).

**FIGURE 6:**
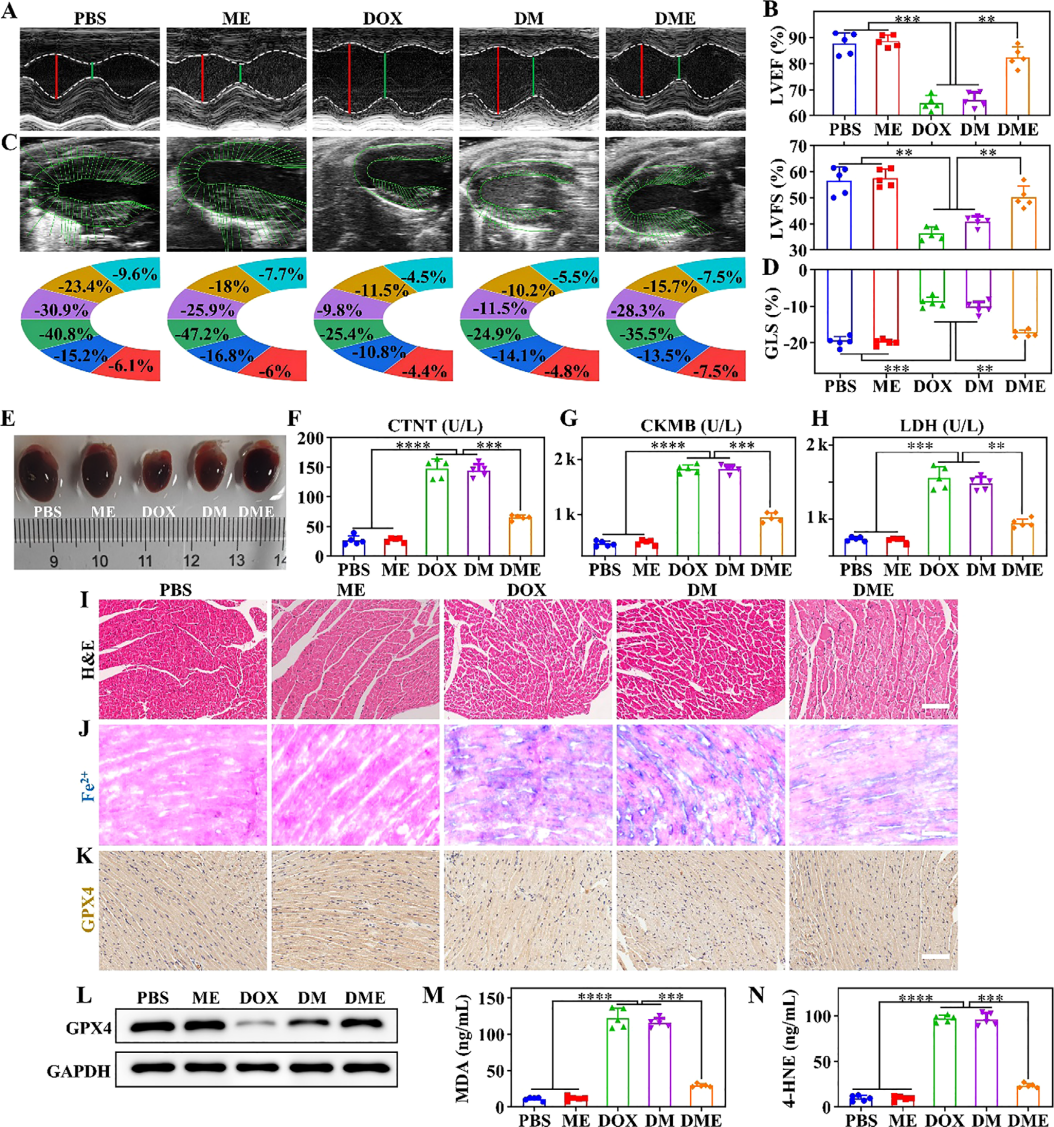
In vivo evaluation of the protective effect of DME against DIC. (A) Representative echocardiographic images of mice from different groups after four weeks of drug administration. The red line indicates the left ventricular internal diameter in end‐diastole (LVIDd), and the green line indicates the left ventricular internal diameter in end‐systole (LVIDs). (B) Left ventricular Ejection Fraction (top) and left ventricular fractional shortening (bottom) in different groups determined by echocardiography. (C) Representative echocardiographic images of the left ventricle with corresponding longitudinal peak strain values in a six‐segment model determined by speckle‐tracking analysis. Segment colors: light blue, anterior base; orange, anterior middle; purple, anterior apex; green, posterior apex; dark blue, posterior middle; red, posterior base. (D) Quantification of Global Longitudinal Strain (GLS), a key parameter reflecting left ventricle longitudinal deformation. (E) Representative photographs of hearts from different treatment groups. (F‐H) Measurement of cardiac injury biomarkers across treatment groups. (I‐K) Histopathological analysis of tissue sections, including H&E staining, Lillie's Fe^2+^ iron staining (blue), and immunohistochemistry for GPX4. Scale bar: 100 µm. (L) Western blot analysis of GPX4 expression in cardiac tissue across treatment groups. (M, N) Measurement of lipid peroxide biomarkers in different treatment groups. ^**^p <0.01, ^***^p <0.001, ^****^p <0.0001.

The cardiotoxicity of anthracycline drugs is classified into three types: acute, chronic, and delayed‐onset. Chronic cardiotoxicity, the most clinically prevalent form, typically arises within the first year of treatment. It primarily manifests as congestive heart failure and/or cardiomyopathy, with echocardiography revealing reduced left ventricular ejection fraction (LVEF). Once established, these changes are irreversible. To recognize this, a chronic progressive cardiotoxicity model, mimicking clinical DOX dosing regimens, was developed to rigorously assess **DME**’s protective efficacy against DIC. The model was established via tail vein injection of DOX (5 mg/kg/week × 4 weeks) or various nanoformulations delivering the same DOX dose. Figure S37 shows DOX tends to accumulate in cardiac tissues. Therapeutic efficacy was evaluated across five groups: PBS, **ME**, DOX, **DM**, and **DME**. Cardiac function was assessed at the experimental endpoint via echocardiography, with primary evaluation metrics including LVEF, left ventricular fractional shortening (LVFS), and global longitudinal strain (GLS). As shown in Figure 6A,B and Movie S1, significant declines in LVEF (63.8%) and LVFS (36.3%) were observed in DOX and **DM** groups vs. controls. Notably, **DME** attenuated these functional impairments (LVEF 80.5%, LVFS 50.2%), validating its cardioprotective efficacy. Additionally, the left ventricle was segmented into six regions for speckle‐tracking analysis, and dynamic GLS curves were acquired throughout the cardiac cycle [37]. As seen in Figure 6C,D, Figure S38 and Movie S2, DOX caused a significant reduction in GLS, whereas partial recovery of cardiac function was observed in the **DME** group, further supporting its cardioprotective efficacy.

Post‐experimental cardiac harvest revealed that the **DME** group exhibited increased heart mass and volume compared with the DOX and **DM** groups, suggesting that **DME** mitigates DOX‐induced suppression of cardiac development (Figure 6E). This effect contributed to the normalization of body weight in **DME**‐treated mice (Figure S39). Blood biochemical analysis showed that **DME** significantly suppressed DOX‐induced elevations in serum cardiac biomarkers, including creatine kinase‐MB (CK‐MB), cardiac troponin T (cTnT), and lactate dehydrogenase (LDH) (Figure 6F–H). These findings indicate that **DME** effectively attenuates DOX‐driven cardiac injury and dysfunction. Histological assessments using H&E staining revealed pathological alterations of DIC, such as poorly defined cellular boundaries, hypertrophy, and myofibrillar fragmentation, all of which were alleviated by **DME** treatment (Figure 6I). Fe^2+^ staining of murine cardiac sections showed minimal detectable free Fe^2+^ in non‐DOX treated groups, because Fe^2+^ exists as transient intermediates in normal tissue (Figure 6J). However, DOX‐induced cardiac injury triggered significant Fe^2+^ accumulation, especially in mitochondria. **DME** treatment substantially reduced free Fe^2+^ levels, which restated **DME**’s iron‐chelating cardioprotection observed in vitro. Furthermore, consistent with ferroptosis inhibition, reduced lipid peroxide biomarkers and downregulated GPX4 expression in **DME**‐treated cardiac tissues, indicating that iron sequestration suppressed key ferroptotic pathways in the heart (Figure 6K–N; Figure S40).

**FIGURE 7:**
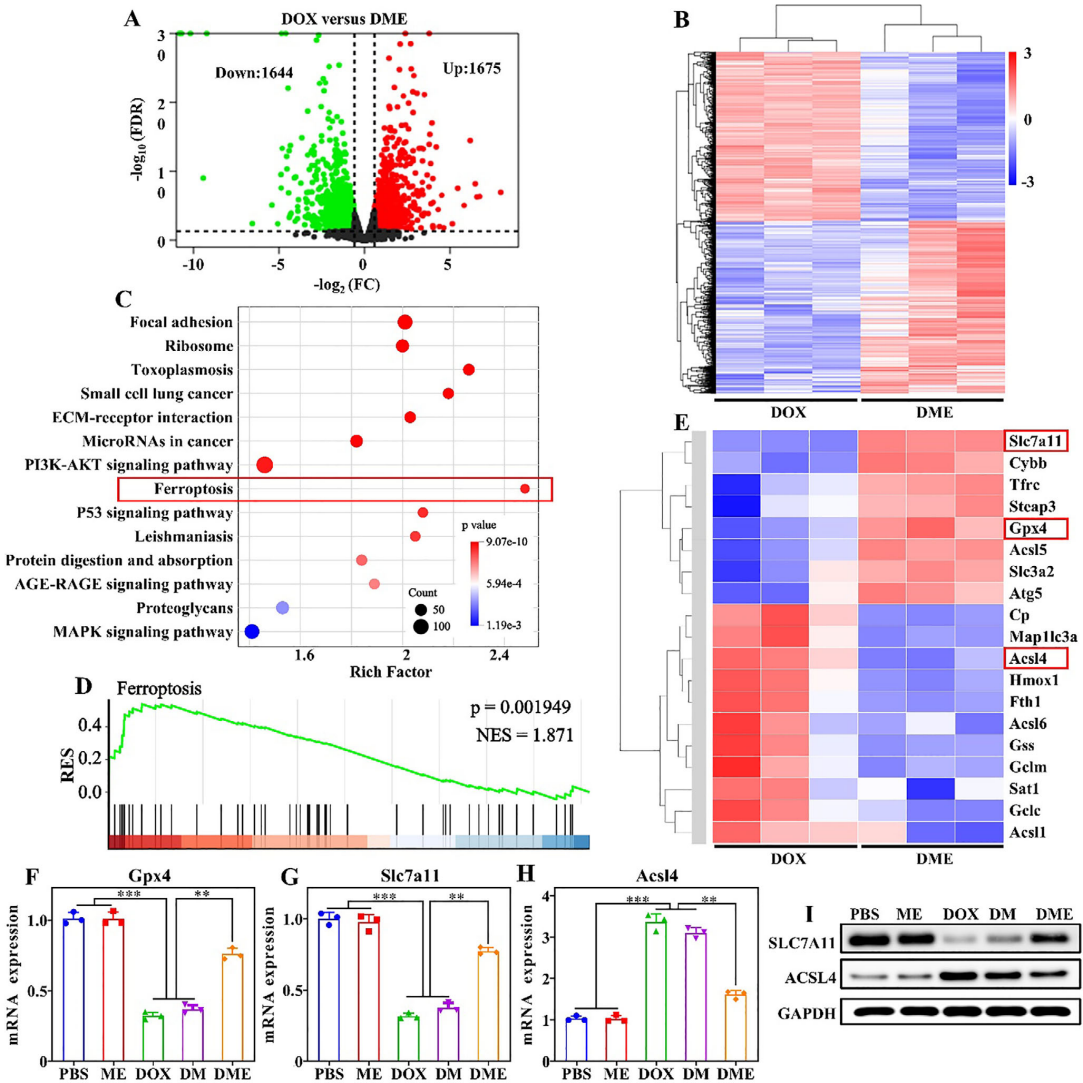
RNA sequencing analysis to investigate the mechanism of DME‐mediated cardioprotection. (A) Volcano plot of cardiac tissues showing the differentially expressed genes following **DME** treatment compared to DOX treatment. (B) Heatmap depicting clustering of the differentially expressed genes. (C) The top 14 enriched pathways identified by KEGG enrichment analysis (DOX vs. **DME**). (D) GSEA of the ferroptosis pathway (DOX vs. **DME**). (E) Heatmap of gene expression in the ferroptosis pathway. (F–H) RT‐PCR analysis of GPX4, SLC7A11, and ACSL4 expressions under various treatment conditions, validating RNA sequencing results. (I) Western blot analysis of the corresponding proteins. ^**^p <0.01, ^***^p <0.001.

To elucidate the molecular mechanism of **DME**’s protective effect against DIC, we performed RNA sequencing on heart tissues from mouse models of cumulative DOX‐induced cardiotoxicity. A total of 3319 differentially expressed genes were identified, comprising 1675 up‐regulated genes and 1644 down‐regulated genes (Figure 7A,B). GO analysis indicated that these differentially expressed genes were predominantly enriched in biological processes such as biological regulation, response to stimulus, and metabolic processes etc., as well as in cellular functions including catalytic activity (Figure S41). Notably, KEGG pathway analysis revealed significant enrichment of ferroptosis‐associated differentially expressed genes (Figure 7C). Combined with Gene Set Enrichment Analysis (GSEA), these findings suggested that **DME** primarily modulates the ferroptosis pathway (Figure 7D). Focusing on ferroptosis‐associated differentially expressed genes, the heatmap showed that **DME** treatment upregulated SLC7A11 of the cystine/glutamate antiporter (System Xc‐) and GPX4, while downregulating the lipid metabolic enzyme ACSL4 compared with the DOX group (Figure 7E) [38, 39]. To validate these key regulators, we performed qPCR and WB, which confirmed the RNA sequencing results (Figure 7F–I; Figure S42).

Interestingly, KEGG enrichment analysis of differentially expressed genes also indicated significant enrichment in the P53 signaling pathway. During the progression of heart disease, activation of the P53 pathway can influence pathological changes in the heart by promoting apoptosis and regulating metabolism, among other mechanisms [40]. Therefore, we further examined the differentially expressed genes within this pathway. As shown in Figures S43 and S44, the downregulation of Trp53 and pro‐apoptotic genes, along with the upregulation of anti‐apoptotic genes in the **DME** group, suggests that **DME** also alleviates cardiotoxicity by inhibiting cardiomyocyte apoptosis. This effect is likely associated with the clearance of free iron ions and suppression of ROS levels. These findings were further confirmed through flow cytometry apoptosis analysis, qPCR, WB, and TUNEL staining in the in vivo evaluation (Figures S45–S48).

Finally, upon completing the in vivo assessment of **DME**, we turned our attention to elucidating its biodegradation pathway. We tracked the biodistribution of **DME** in healthy mice at 1, 7, and 30 days following intravenous administration. The concentration of Hf^4+^ was measured by ICP‐MS. As shown in Figure S49, Hf was initially highly enriched in the liver, kidney, and spleen at 24 h, followed by gradual clearance from all major organs over time. Meanwhile, detection of significant Hf levels in feces and urine within the first few days confirms excretion of the material via renal and fecal pathways (Figure S50). Considering the high stability of the Hf─O coordination bonds, we speculate that **DME** undergoes in vivo breakdown into small molecules and Hf─O clusters, which are subsequently metabolized and excreted [41].

## 3 Discussion

Does **DME**’s iron chelation compromise anti‐tumor efficacy? Our data indicates that EDTA modification exerts no significant negative impact. Mechanistically, EDTA disrupts cell membrane stability via phospholipid interaction and calcium ion chelation, thereby enhancing **DME**’s tumor penetration for deep‐tissue drug delivery (Figure 3A,B; Figure S5) [32]. Additionally, combined radio‐chemotherapy synergistically improves therapeutic outcomes, including in drug‐resistant tumors. Importantly, DOX's DNA‐targeting properties complement radiation‐induced DNA damage by inhibiting repair and promoting apoptosis [42]. Thus, EDTA functionalization positively contributes to tumor therapy. Regarding cardioprotection, is iron chelation of **DME** an effective strategy against DIC? The heart contains uniquely high iron levels compared to other organs [6, 43], and DOX preferentially accumulates in cardiac mitochondria due to its strong affinity for cardiolipin on the inner mitochondrial membrane [12, 13]. The resulting DOX‐Fe complexes trigger excessive lipid peroxidation even without H_2_O_2_, driving mitochondrion‐dependent ferroptosis, a key factor in DIC progression [24, 25]. Mitochondrial Fe^2+^ accumulation further elevates oxidative stress, inducing apoptosis. Therefore, spatiotemporally targeted iron chelation at DOX leakage sites is critical to prevent DIC. **DME** achieves immediate local iron scavenging, while EDTA's superior coordination capacity: (1) deprives Fe from DOX‐Fe complexes (Figure 2A–D), and (2) permanently inactivates catalytic centers in **Fe@DME** via multi‐dentate binding, blocking Fe‐mediated redox cycling and preventing ROS/lipid peroxides accumulation (Figures 5B‐D and Figure S34). Both in vitro and in vivo data demonstrate that **DME**’s spatiotemporally matched iron‐chelating strategy effectively mitigates DIC.

In clinical practice, dexrazoxane is used to mitigate DIC. However, the timely administration of this antidote is often not feasible once cardiac injury. A major challenge in DIC management lies in the spatiotemporal mismatch between the two agents. Unlike this, our designed nanoplatform integrates iron chelation and DOX delivery into a single system to address DIC. It simultaneously removes free Fe^2+^ during DOX release, thereby spatiotemporally matching the toxic mechanism of DOX and achieving effective cardiotoxicity mitigation. Consequently, this “spatiotemporally matched” iron chelation strategy represents an innovative approach for mitigating DIC, as it precisely targets the toxic mechanism by aligning chelation with DOX's pharmacokinetics and site of injury.

## 4 Conclusion

Clinical management of anthracycline‐induced cardiotoxicity remains challenging, with limited therapeutic options. Adjunctive cardiovascular drugs often produce pharmacodynamic interactions that can compromise anticancer efficacy. Conventional systemic administration further fails to achieve spatiotemporal precision in controlling cardiotoxicity, frequently resulting in irreversible cardiac structural damage. To address these limitations, we engineered EDTA‐functionalized Hf‐based MOFs, creating a novel interdisciplinary approach in onco‐cardiology. The resulting **DME** nanomedicine provides dual benefits: enhanced tumor therapy through synergistic radio‐chemotherapy and cardioprotection via localized iron chelation. By integrating tumor treatment with cardiac safeguarding, this strategy establishes a new paradigm in onco‐cardiology and offers a pathway toward next‐generation chemotherapy with improved safety profiles. Our ongoing research aims to develop multifunctional nanotherapeutics that simultaneously boost efficacy and mitigate toxicities, with particular focus on clinical immunoadjuvants and antibody‐drug conjugates in oncology.

## 5 Experimental Section

### 5.1 Synthesis of MOF‐808‐Hf, ME, DM, DME, MEC, and DMEC Nanoparticles

MOF‐808‐Hf was synthesized via a solvothermal method. Briefly, 122.9 mg HfOCl_2_∙8H_2_O and 21 mg trimesic acid were dissolved in 6 mL of a DMF/formic acid mixed solvent (2:1 v/v). After ultrasonication for 30 min, the solution was transferred into a high‐pressure reactor and heated 100°C for 24 h. Once cooled to room temperature, the precipitate was collected by centrifugation (12 000 rpm, 5 min) and washed twice with DMF and ethanol. The obtained solid was dried and activated in a vacuum oven at 100°C for 12 h to yield MOF‐808‐Hf.

To prepare **ME**,100 mg of MOF‐808‐Hf was mixed with 1.86 g EDTA‐2Na in 50 mL water, and the reaction was carried out at 60°C for 24 h. **DM** and **DME** were prepared by stirring MOF‐808‐Hf or **ME** powders with DOX (2:1 mass ratio) in DMF at room temperature for 18 h without light. The products were washed twice with DMF/ethanol and subsequently stored in ethanol.

For synthesis of **MEC** and **DMEC**, MCF‐7 or MCF‐7/ADR cells in optimal growth condition were harvested. A total of 1 × 10^7^ cells were digested with trypsin and collected by centrifugation (1000 rpm for 5 min). The cell pellet was resuspended in 1 mL of pre‐cooled ultrapure water and subjected to ultrasonic treatment at 4°C for 5 min to induce lysis. The suspension then underwent two freeze‐thaw cycles in liquid nitrogen, followed by centrifugation (12 000 rpm, 10 min) to isolate the cell membranes. The obtained membranes were dispersed in PBS containing **ME** or **DME** nanoparticles and performed multiple extrusion cycles. Finally, the resulting **ME** or **DMEC** nanoparticles were washed twice with PBS, centrifuged (12 000 rpm, 10 min), and redispersed in PBS.

The drug loading capacity (LC) and encapsulation efficiency (EE) were determined by measuring the UV–vis absorption of DOX at 480 nm before and after the loading process. In brief, DOX standard solutions (0, 0.5, 1, 2, and 4 mg/mL) were prepared in DMF, and their UV–vis absorption was measured at 480 nm. The concentration of encapsulated DOX was determined using the established calibration curve. The LC and EE were calculated based on the formulas:

$$LC\left(\%\right)=\frac{Mass\;of\;loaded\;DOX}{Mass\;of\;DME}\times 100\%$$

$$EE\left(\%\right)=\frac{Mass\;of\;loaded\;DOX}{Total\;Mass\;of\;DOX\;employed}\times 100\%$$

### 5.2 Study of Fe^2+^‐Chelating Capability of ME in Chemical Systems

To evaluate the Fe^2+^‐chelating ability of **ME**, 0.1 mg/mL FeCl_2_·4H_2_O was added to 0.1 mg/mL DOX solution and stirred for 30 min to form DOX‐Fe complexes. **ME** at various concentrations (0, 0.1, 0.2, 0.3, 0.4, and 0.5 mg/mL) were then added to the mixture and incubated for 30 min. The samples were centrifuged, and the supernatants were collected for Fe^2+^ concentration measurement. To further investigate the chelation state of Fe in **Fe@ME**, the same procedure was repeated using 0.4 mg/mL **ME**. The corresponding supernatant and precipitate were analyzed by UV–vis spectroscopy and XPS, respectively.

For kinetic studies of the competitive coordination reaction, 0.1 mg/mL FeCl_2_·4H_2_O and 0.1 mg/mL DOX were reacted at room temperature for 30 min, after which 0.4 mg/mL **ME** was added. The mixtures were incubated for varying times (0, 5, 10, 20, and 30 min, 1, 2, 3, 6, 12, and 24 h), followed by centrifugation. The supernatants were collected for Fe^2+^ quantification, and concentration‐time curves were plotted. Non‐linear fitting was performed using OriginPro 2021.

The catalytic activity of DOX‐Fe and **Fe@DME** was assessed in a Fenton reaction system. A mixture of coumarin (1 mm) and H_2_O_2_ (400 µm) was added to aqueous solutions of DOX‐Fe and **Fe@DME** (pH 7.4), each containing equivalent concentrations of Fe^2+^ ions. The reactions were incubated in the dark for 4 h, followed by centrifugation. The fluorescence intensity of the resulting supernatants was measured at λ_ex_/λ_em_ = 345/453 nm. Each experimental group was performed in six replicates.

### 5.3 Study of •OH Generation Under X‐ray Irradiation in Aqueous Phase

Four sets of 3 mL aqueous solution samples, including PBS, DOX (equivalent to the DOX loading in **DME**), **ME** (40 ppm based on Hf), and **DME** (40 ppm based on Hf), were each mixed with 1 mm coumarin and exposed to X‐ray radiation at doses of 0, 5, 10, 15, and 20 Gy. The resulting solutions were immediately analyzed by fluorescence spectroscopy (λ_ex_/λ_em_ = 345/453 nm). Each group was tested in six replicates.

### 5.4 3D Cell Sphere Uptake Assay

MCF‐7 cells were seeded into 6‐well plates and incubated for 24 h. After trypsin digestion, the cells were diluted to 500 cells/µL and seeded into ultra‐low adhesion 96‐well plates (200 µL per well). The medium was replaced every 2 days for a total of 6 days. The resulting cell spheres were then treated with DOX (2 µm), **DM** (2 µm, based on DOX), or **DME** (2 µm, based on DOX) for 12 h. Uptake was visualized by CLSM through DOX fluorescence.

### 5.5 Cytotoxicity Assay

MCF‐7 or MCF‐7/ADR cells (4 × 10^3^ cells/well) were seeded into 96‐well plates and incubated for 24 h. The medium was then replaced with fresh medium containing PBS, DOX, **ME**, or **DME** (1, 2, or 4 µm for MCF‐7; 2.5, 5, or 10 µm for MCF‐7/ADR, all based on DOX) and incubated for 12 h. Cells were subsequently irradiated with 0 or 4 Gy of X‐rays and incubated for another 12 h. Cell viability was assessed by adding 10 µL of CCK‐8 solution to each well, followed by incubation for 2 h. The absorbance at 450 nm was measured using a microplate reader, with the amount of orange formazan dye directly proportional to the number of viable cells.

### 5.6 Detection of Intracellular ROS and •OH

MCF‐7 cells (1 × 10^5^ cells/mL) were seeded in confocal culture dishes and incubated for 24 h. The medium was then replaced with fresh PBS, DOX, **ME**, and **DME** (2 µm, based on DOX) and incubated for an additional 12 h. Cells were subsequently exposed to X‐ray irradiation at either 0 or 4 Gy, followed by 1 h incubation. After removing the medium, DCFH‐DA and Hoechst 33 342 were added and incubated for 30 min to visualize ROS using CLSM. Detection of •OH was carried out using the same procedure, except hydroxyphenyl fluorescein (HPF) was used as the indicator.

### 5.7 *γ*‐H2AX Immunofluorescence Assay

MCF‐7 cells were seeded in confocal dishes for 12 h. Culture mediums were replaced with fresh mediums containing PBS, DOX, **ME**, or **DME** (2 µm, based on DOX) and incubated for an additional 12 h. The cells were irradiated with 0 or 4 Gy of X‐rays, followed by 1 h incubation. Subsequently, the cells were fixed, permeabilized, blocked, and incubated with primary and secondary antibodies. Nuclear staining was performed with DAPI, and DNA damage was visualized using CLSM.

### 5.8 Isolation and Culture of Primary Cardiomyocytes

Newborn C57BL/6J mice (2‐day‐old) were euthanized by decapitation, and their hearts were collected and rinsed with pre‐cooled PBS. The heart tissue was minced into ∼1 mm^3^ fragments and transferred into a digestion solution containing 0.25% trypsin and 0.025% type IV collagenase, followed by incubation at 37°C for 5 min. The remaining tissue was subjected to ten further digestions under the same conditions. All supernatants were pooled and transferred into DMEM/F12 medium supplemented with 10% FBS to terminate enzymatic digestion. After filtration through a 100 µm mesh filter, the cell suspension was centrifuged at 1000 rpm for 5 min. The resulting pellet was resuspended and plated in DMEM/F12 medium containing 10% FBS and 1% penicillin streptomycin for 1 h at 37°C. Adherent cells were discarded, and the non‐adherent cells in the supernatant were transferred to new dishes. After 24 h of culture, the primary cardiomyocytes were ready for subsequent experiments.

### 5.9 Analysis of Fe^2+^ Scavenging in Cardiomyocytes

Primary cardiomyocytes (4 × 10^5^ cells/well) were seeded into 6‐well plates and cultured for 24 h. The cells were then treated with PBS, **ME**, DOX, **DM** and **DME** (2 µm, based on DOX) for 12 h. After treatment, the cells were incubated with Fe^2+^ indicator probes for 20 min, washed with PBS, and stained with FerroOrange. Intracellular Fe^2+^ fluorescence was observed by CLSM.

### 5.10 Mitochondrial Membrane Potential Analysis

Primary cardiomyocytes (4 × 10^5^ cells/well) were seeded into 6‐well plates and cultured for 24 h. The cells were then treated with PBS, **ME**, DOX, **DM**, and **DME (**2 µm, based on DOX) for 24 h. Mitochondrial membrane potential was assessed using a JC‐1 kit according to the manufacturer's introductions. Nuclei were counterstained with Hoechst 33 342 for 15 min. The samples were analyzed by CLSM and flow cytometry.

### 5.11 Lipid Peroxide Analysis

Primary cardiomyocytes (4 × 10^5^ cells/well) were seeded into 6‐well plates and cultured for 12 h. The cells were then incubated with PBS, **ME**, DOX, **DM** and **DME** (2 µm, based on DOX) for 24 h. Lipid peroxidation was evaluated by staining with C11‐BODIPY, followed by imaging with CLSM.

### 5.12 Bio‐TEM

Primary cardiomyocytes (4 × 10^5^ cells/well) were seeded into 6‐well plates and cultured for 12 h. The cells were then treated with PBS, **ME**, DOX, **DM**, and **DME** (2 µm, based on DOX) for 24 h, harvested, and fixed with 4% glutaraldehyde at 4°C. Secondary fixation was performed with 1% osmium tetroxide for 1 h. After graded dehydration with alcohol and acetone, the samples were embedded in Epon816 resin. Ultrathin sections were prepared, stained with acetic acid uranyl and lead citrate, and imaged using TEM.

### 5.13 Animal Experiments

Six‐week‐old female BALB/c nude mice (∼19 g) and eight‐week‐old male C57BL/6 mice (∼20 g) were obtained from Vital River Laboratory Animal Technology (Beijing, China). All animal procedures were performed in accordance with the guidelines and regulations of the Guangzhou Medical University (Approval No. GY‐2024‐303).

### 5.14 Tumor Penetration Analysis of DM and DME

To evaluate the tumor penetration, DOX, **DM**, and **DME** (2 mg/mL, based on DOX) were administered via peritumoral injection to MCF‐7 tumor‐bearing BALB/c nude mice (*n* = 3). After 12 h, the mice were euthanized, and tumors were excised. The tissues were immediately fixed in paraformaldehyde, processed into frozen sections, stained with DAPI, and imaged using CLSM.

### 5.15 In Vivo Anti‐Tumor Effect

Bilateral xenograft models were established in BALB/c nude mice by subcutaneous injection of 1 × 10^7^ MCF‐7 or MCF‐7/ADR cells. When tumor volume reached ∼100 mm^3^, the mice were randomly divided into four groups (*n* = 6), and treated intravenously with PBS, **ME**, DOX, and **DMEC** (2 mg/kg for MCF‐7, 5 mg/kg for MCF‐7/ADR, based on DOX). After 12 h of administration, tumors on the right flank were irradiated with 4 Gy of X‐rays, while those on the left flank were shielded. A second round of treatment was given 7 days later. During the 14‐day treatment period, tumor volumes and body weights were measured every 2 days. Tumor volume was calculated using the formula:

$$V=\frac{length\times width^{2}}{2}$$

Two days after the second radiotherapy, one mouse from each group was euthanized, and tumors were harvested for pathological analysis, including H&E staining, TUNEL assay, and immunohistochemistry. On day 14, all remaining mice were euthanized, and tumors were obtained for imaging.

### 5.16 Establishment of the DIC Mouse Mode

C57BL/6J mice were randomly divided into 5 groups (*n* = 5): PBS, **ME**, DOX, **DM** and **DME**. A cumulative DIC mice model was established by intravenous injections of DOX (5 mg/kg/week) for 4 weeks. Mice in the **ME**, **DM** and **DME** groups received the same DOX dosing schedule. Subsequent experiments, including echocardiography, were performed after model establishment.

### 5.17 Echocardiography Analysis

Cardiac function was evaluated using high‐resolution echocardiography (Vevo 3100 animal ultrasound imaging system). LVEF and LVFS values were calculated from an M‐Mode image of the parasternal short axis view using Vevo Analysis software. Based on the parasternal long axis view in B‐Model, strain rate and GLS of left ventricle were calculated. The formulas are as follows:

$$LVEF=\frac{EDV-ESV}{EDV}\;\times 100\%$$

$$LVFS=\frac{LVIDd-LVIDs}{LVIDd}\;\times 100\%$$

$$GLS=\frac{L-L_{0}}{L}\;\times 100\%$$

where EDV is the End‐diastolic volume of left ventricular, ESV is the End‐systolic volume of the left ventricle, L_0_ is the Initial length of myocardium in end‐diastole, L is the length of myocardium after contraction in end‐diastole.

### 5.18 Statistical Analysis

All experiments were performed at least three times. Data are expressed as mean ± standard deviation (SD). Statistical analyses were conducted using GraphPad Prism version 8. One‐way analysis of variance (ANOVA) followed by Tukey post‐hoc test was used for comparisons among groups. A *p* value <0.05 was considered statistically significant, with significant indicated as: ^*^p <0.05, ^**^p <0.01, ^***^p <0.001, ^*^ ^***^p <0.0001.

## Funding

National Key R&D Program of China 2022YFE0209700 (X. Yu), National Natural Science Foundation of China 22175046, 21805090 (T. Gong), and 22205044 (Y. Li)

## Conflicts of Interest

The authors declare no conflicts of interest.

## Supporting information




**Supporting File 1**: advs74322‐sup‐0001‐SuppMat.docx.

**Supporting File 2**: advs74322‐sup‐0002‐Movie S1.mp4.

**Supporting File 3**: advs74322‐sup‐0003‐Movie S2.mp4.

## Data Availability

The data that support the findings of this study are available from the corresponding author upon reasonable request.
