# Supplementary material for: EDTA‐Functionalized Nanoscale Metal–Organic Framework for Onco‐Cardiology via Radiochemotherapy Synergy and Spatiotemporally Matched Iron Chelation
Source: Adv Sci (Weinh). 2026 Feb 8;13(22):e21451. doi: 10.1002/advs.202521451 (PMC13088290; doi:10.1002/advs.202521451)
Supplement: Supplementary file 1 — Supporting File 1: advs74322‐sup‐0001‐SuppMat.docx. [file ADVS-13-e21451-s001.docx]

Supporting Information

**EDTA-Functionalized Nanoscale Metal-Organic Framework for Onco-Cardiology via Radiochemotherapy Synergy and Spatiotemporally Matched Iron Chelation**

*Daojing Yuan^1†^, Liyang Tian^1†^, Luwen Zhuang^2†^, Yongyu Liang^1^, Lingfei Chen^1^, Xuetao Wang^3^, Yanli Li^1*^, Xiyong Yu^1*^, and Teng Gong^1*^*

^1^Guangzhou Municipal and Guangdong Provincial Key Laboratory of Molecular Target & Clinical Pharmacology, the NMPA and State Key Laboratory of Respiratory Disease, School of Pharmaceutical Sciences, Guangzhou Medical University, Guangzhou 511436, China

^2^Center for Water Resources and Environment, and Guangdong Key Laboratory of Marine Civil Engineering, School of Civil Engineering, Sun Yat-sen University, Guangzhou 510275, China

^3^The Second Affiliated Hospital of Guangzhou University of Chinese Medicine, Guangzhou 510 0 06, PR China

^*^Corresponding author. Email: ylli@gzhmu.edu.cn (Y.L.); yuxycn@gzhmu.edu.cn (X.Y.); tgong@gzhmu.edu.cn (T.G.)

^†^These authors contributed equally to this work.

Experimental Section

**Materials.** Doxorubicin (DOX), hafnium dichloride oxide octahydrate (HfOCl_2_∙8H_2_O), N, N-Dimethylformamide (DMF), ethylenediaminetetraacetic acid disodium salt (EDTA-2Na), formic acid, and trimesic acid were purchased from Aladdin Biochemical Technology (Shanghai, China). Cell counting kit-8 (CCK-8), 2’,7’-dichlorodihydrofluorescein diacetate (DCFH-DA), 5-ethynyl-2’-deoxyuridine (EdU), C11-BODIPY and sodium dodecyl sulfate polyacrylamide gel electrophoresis (SDS-PAGE) kits were purchased from Beyotime Biotechnology (Haimen, China). Lillie Fe^2+^ staining kit was purchased from Solarbio Science & Technology (Beijing, China). Dulbecco’s Modified Eagle Medium (DMEM), Dulbecco’s Modified Eagle’s Medium/Ham’s F12 Nutrient Medium (DMEM/F12), Roswell Park Memorial Institute 1640 (RPMI 1640), and fetal bovine serum (FBS) were purchased from Procell Biotechnology (Wuhan, China). Antibodies (Bax, Bcl-2, GPX4, SLC7A11, ACSL4, P53, GAPDH and Tubulin) were purchased from Proteintech Biotechnology (Wuhan, China). γ-H2AX antibody was purchased from Abcam (United Kingdom). Annexin V/Red Nucleus Ⅱ kit was purchased from UElandy Biotechnology (Suzhou, China). 4-HNE (4-Hydroxynonenal) ELISA and MDA (Malondialdehyde) ELISA kits were purchased from Elabscience (Wuhan, China).

**Characterization.** The morphologies and element distribution of samples were observed by a scanning electron microscopy (SEM, JSM-7800F) and a transmission electron microscopy (TEM, JEM-2800). DLS analysis was obtained with Malvern Zetasizer Nano ZSZEN3600 (United Kingdom). UV-vis spectra were obtained with a Shimadzu RF-5301PC spectrophotometer (Japan). Fourier-transform infrared (FT-IR) spectra were obtained with a Hyperion1000 Spectrometer (Bruker, Germany). X-ray photoelectron spectroscopy (XPS) spectra were obtained by an X-ray photoelectron spectrometer (Thermo Scientific ESCALAB 250Xi). The ^1^H nuclear magnetic resonance (NMR) spectra were obtained using JNM-EC Z400S/L1 (JEOL, Japan) with D_2_O containing KOH as the solvent, in which chemical shifts were recorded. Confocal laser scanning microscopy (CLSM) images were obtained with a Zeiss LSM800 microscope (Germany). Ion concentrations were recorded with ICP-OES 5800. Flow cytometer results were obtained with NovoCyte 3005 (Agilent, USA). Western blot images were obtained by Bio-Rad Chemi Doc XRS System (USA). The in vivo luminescence imaging of mice was detected on a PerkinElmer Health Sciences IVIS Spectrum bioluminescence imager (USA) and analyzed by Living Image 4.5.5 Software. Echocardiographic images were obtained with Vevo 3100 (Canada). The X-rays used in the evaluation of chemical systems and cellular levels are derived from an X-ray irradiation instrument Rad Source RS2000pro (160 KV X-rays with the does rate 7.2 Gy/min). The animal radiotherapy experiment was performed in the Second Affiliated Hospital of Guangzhou University of Chinese Medicine. 6 MeV X-rays derived from VARIAN Trilogy linear accelerator were used with the does rate 4 Gy/min.

**Drug release.** The drug release was assessed by dispersing 3 mg **DME** into 3 mL PBS buffer at pH 5.4 and 7.4 for incubation without light. The fluorescence of DOX in the supernatant was measured at various time points, quantified using a standard curve, and the drug release profile was plotted accordingly.

**Fe^2+^ chelation selectivity.** A 1 mL ethanol solution containing 2 mg/mL **ME** (with 2.1 mM EDTA) was prepared. To this solution, 1 mL of 0.55 mg/mL CaCl_2_ in ethanol solution (5 mM) was added, and the mixture was allowed to react at room temperature for 1 hour. The resulting precipitates were collected by centrifugation (12000 rpm, 5 min), washed twice with ethanol, and then ultrasonically dispersed in ethanol to obtain the **Ca@ME** dispersion. Subsequently, 1 mL of 0.64 mg/mL FeCl_2_ ethanol solution (5 mM) was induced, and the reaction proceeded for another 1 hour. The product of the coordination substitution was isolated using the same centrifugation and washing procedure. Finally, the color changes of the solutions were recorded, and elemental mapping analysis was conducted.

**Ca@DME** was synthesized following the same procedure. After isolating and culturing cardiomyocytes for 24 hours, the cells were treated for another 24 hours with DOX (2 μM), **DM** (2 μM based on DOX), **DME** (2 μM based on DOX), and **Ca@DME**. Finally, FerroOrange probe staining was performed, followed by laser confocal imaging.

**Cell culture.** MCF-7 cells were cultured with DMEM medium containing 10% FBS and 1% penicillin-streptomycin. MCF-7/ADR cells were incubated in 1640 medium supplemented with 10% FBS and 1% penicillin-streptomycin. All cells were maintained under conditions of 37 ℃, 5% CO_2_ and 95% air.

**Cellular** **uptake analysis.** MCF-7 cells (1×10^5^ cells/mL) were seeded in confocal dishes and incubated for 24 h. After that, DOX (2 μM), **DM** (2 μM based on DOX), and **DME** (2 μM based on DOX) were incubated with MCF-7 cells in DMEM respectively. After 6 h incubation, cells were washed twice with PBS and subjected to confocal microscope imaging by the fluorescence of DOX. Additionally, the treated cells were resuspended by trypsin, washed with PBS, and recorded the fluorescence of DOX by a flow cytometer.

**Colony formation assay.** MCF-7 cells or MCF-7/ADR cells were seeded into 6-well plates at various densities (600, 800 and 1000 cells/well) for 24 h. The cells were treated with PBS, **ME**, DOX, DME (2 μM for MCF-7, 5 μM for MCF-7/ADR based on DOX) for 12 h. Subsequently, 600 cells were irradiated with 0 Gy or 2 Gy X-rays, 800 cells were irradiated with 4 Gy of X-rays, and 1000 cells were irradiated with 8 Gy of X-rays. After irradiation, the cells were washed with PBS, and fresh culture mediums were used to incubate the cells for another 10 days. After being fixed with paraformaldehyde, the cells were stained with crystal violet. A cell colony should contain at least 50 cells. or each group, the experiment will be carried out with three replicates. The sensitization enhancement ratios (SER) were evaluated using multitarget single-hit models. The formula is as follows ^[1]^:

*SF* = 1 − (1− *e*^−^*^D^* ^/^ *^D0^*) *^N^*

ln *N* = *D_q_ / D_0_*

*SER* = *D_q_* (PBS groups) / *D_q_* (other groups)

where SF, D, D_0_, N and Dq represent the survival fraction, radiation dose, mean lethal dose, extrapolation number and quasi-threshold dose, respectively.

**Comet assay.** MCF-7 cells (1×10^5^ cells/mL) were seeded in 6-well plates for 24 h. Then, the culture mediums were replaced to fresh mediums containing PBS, DOX, **ME**, or **DME** (2 μM based on DOX). Following 12 h treatment, these cells were irradiated with 0 Gy and 4 Gy of X-rays. For another 12 h incubation, the cells were washed three times with PBS. Finally, cells were digested and harvested, and the standard single cell gel electrophoresis procedure was employed to observe the DNA damage by CLSM.

**EdU assay.** MCF-7 cells were seeded in confocal dishes for 24 h. After removing the old mediums, cells were co-incubated with PBS, DOX, **ME**, or **DME** (2 μM based on DOX) for 12 h. Subsequently, the cells were irradiated with 0 Gy or 4 Gy of X-ray. Following 12 h incubation, culture mediums were replaced with fresh mediums containing EDU working solution I according to the kit instructions and incubated for 2 h. After washing twice with PBS, the cells were fixed with polyformaldehyde and stained with working solution II. Finally, these cells were imaged by CLSM.

**Cellular cycle analysis.** MCF-7 cells were seeded in 6-well plates for 24 h, followed by replacement of the old culture mediums with fresh mediums containing PBS, DOX, **ME**, and **DME** (2 μM based on DOX) for an additional 12 h treatment. Subsequently, the cells were irradiated with 0 Gy or 4 Gy of X-ray. After 12 hours, the cells were harvested and fixed in 75% ice-cold ethanol for 30 minutes. Following fixation, they were washed twice with PBS and then stained according to the manufacturer's protocol provided with the cell cycle detection kit. Finally, the cellular cycles were analyzed by flow cytometry.

**Cell apoptosis assessment.** MCF-7 cells were cultured in 6-well plates for 24 h. Then, the mediums were replaced with fresh mediums containing PBS, DOX, **ME**, and **DME** (2 μM based on DOX) for 12 h. Subsequently, the cells were irradiated with 0 Gy or 4 Gy of X-rays and further cultured for 12 h. After collecting cells by trypsin and dispersing them in PBS, the suspensions were incubated with Annexin V/Red Nucleus Ⅱ for 15 min in the dark. Finally, the apoptosis levels of the cells were detected by flow cytometry.

**Western blot analysis.** MCF-7 cells were seeded in 6-well plates for 24 h, followed by replacement of the old culture mediums with fresh mediums containing PBS, DOX, **ME**, and **DME** (2 μM based on DOX) for additional 12 h treatment. Subsequently, the cells were irradiated with 0 Gy or 4 Gy of X-ray. After continuing incubation for 12 h, cell lysate was prepared according to protein extraction kit instructions. These lysates were separated by SDS-PAGE and transferred to polyvinylidene fluoride (PVDF) membranes. After blocking with 5% skim milk for 1 hour, the membranes were incubated with specific primary antibodies (Bax, γ-H2AX, Bcl-2 and GAPDH) overnight at 4 °C and followed by HRP-conjugated goat anti-rabbit lgG for 1 h. Finally, protein images were obtained using Bio-Rad Chemi Doc XRS System. Additionally, the protein expression level of GPX4, SLC7A11, ACSL4, P53, Bax, Bcl-2, Tubulin and GAPDH were also measured in primary cardiomyocytes. Cardiomyocytes were cultured in 6-well plates and treated with PBS, DOX, ME, DM, or DME (2 μM based on DOX) for 24 h. Following the above protocol, Western blot images were obtained.

**Biosafety assessment of DMEC *in vivo*.** Firstly, the BALB/c nude mice were randomly divided into three groups (n = 4). The mice were administered either PBS or **(**5 mg/kg based on DOX) via intravenous injection at a volume of 150 μL. Subsequent body weight changes were monitored at two-day intervals. Blood samples (200 μL) were collected via retro-orbital bleeding for hematological analysis after the mice were euthanized at the 3-day and 30-day time points. The Mindray automatic blood cell analyzer (BC-2800 Vet) was used to detect the complete blood panel parameters, including aminotransferase (ALT), alanine aspartate aminotransferase (AST), nephric blood alkaline phosphatase (ALP), urea nitrogen (UREA), creatinine (CREA) white blood cells (WBC), red blood cells (RBC), hemoglobin (HGB), hematocrit (HCT), mean corpuscular hemoglobin concentration (MCHC) and lymphocytes (LYM). Furthermore, the major organs of mice were collected for H&E section staining.

**Biodistribution and blood circulation study.** Indocyanine green (ICG) was used instead of DOX (termed **ICG@ME** and **ICG@MEC**) to trace the distribution of nanomedicine. Briefly, female MCF-7 tumor-bearing nude mice (n = 3) were intravenously injected with ICG, **ICG@ME**, or **ICG@MEC** (at a dose of 2.5 mg/kg ICG equivalent). Bioluminescence imaging was performed at predetermined time points (4, 8, 12, and 24 h post-injection).

Biodistribution of **DMEC** in major organs of mice was further evaluated. The female MCF-7 tumor-bearing nude mice (n = 3) were intravenously injected with **DMEC** (2 mg/kg based on DOX) and sacrificed after 12 h, 24 h and 48 h post injection. The major organs (heart, liver, spleen, lung, and kidney) and tumors were excised and weighed. Finally, all tissues were fully dissolved in aqua regia and the concentrations of Hf were measured via ICP-OES.

To investigate the blood circulation half-life of **DMEC**, the mice (n = 3) were intravenously injected with **DMEC** (2 mg/kg based on DOX). Then, 10 μL blood was acquired from the mice at different time points (10 min, 20 min, 30 min, 40 min, 1 h, 2 h, 4 h, 6 h, 12 h and 24 h) and diluted with 10 mM EDTA-2Na as anticoagulant. After being fully dissolved, blood was centrifuged to obtain supernatants and the concentration of Hf was measured by ICP-OES.

**Evaluation of Fe^2+^ scavenging in cardiac tissue.** Following the successful induction of the DIC mouse model, cardiac tissues were harvested from mice in each treatment group. These cardiac tissues were fixed with polyformaldehyde and sliced into frozen sections. Sections were stained according to the instructions of Lillie Fe^2+^ staining kit and imaged by CLSM.

**Detection of 4-HNE and MDA in cardiac tissue.** After finishing establishment of the DIC mouse model, the extraction of cardiac tissues from mice in each treatment group was performed, followed by washes with ice-cold PBS to eliminate any remaining blood. These cardiac tissues were mixed with a corresponding volume of protease-inhibited PBS (9 mL PBS per 1 g tissue) and then homogenized in an ice bath. The homogenates were centrifuged at 12000 rpm for 10 min at 4 °C to separate the supernatant. The concentrations of 4-HNE and MDA were measured using commercial ELISA kits.

**RNA-sequencing analysis.** Upon completion of the 4-week DIC model establishment, hearts were collected from DOX- and **DME**-treated mice (n=3) for RNA sequencing. The total mRNA of cardiac tissues was obtained by TRIzol method and mRNA integrity was assessed by Agilent Bioanalyzer 2100 system. Then, each sample used 1 μg mRNA as input material for sequencing libraries preparation. Libraries were constructed using the Hieff NGS Ultima Dual-mode mRNA Library Prep Kit for Illumina (Yeasen Biotechnology). After preparation of the library, the data analysis was performed using BMKCloud (www.biocloud.net). Briefly, data analysis was performed using R Foundation for Statistical Computing and used DESeq2 to normalize the raw counts and identify differentially expressed genes (fold change ≥ 1.5, false discovery rate < 0.05). Study employed the R package cluster profiler for Gene Ontology (GO) enrichment analysis. The selected differentially expressed genes were used as input data and analyzed through the Kyoto Encyclopedia of Genes and Genomes (KEGG) functional enrichment analysis. Gene Set Enrichment Analysis (GSEA) was applied to verify whether these research-worthy gene sets exhibited significant enrichment under corresponding experimental conditions.

**Quantitative reverse transcription PCR (qPCR)**. Primary cardiomyocytes (4×10^5^ cells/well) were seeded into 6-well plates for 24 h. Then, the cells were incubated with PBS, ME, DOX, **DM** and **DME** (2 μM based on DOX). After another 24 h of incubation, the total RNA of cells was extracted by TRIzol reagent (Yeasen Biology) and then transcribed into cDNA by the Evo M-MLV RT kit (Accurate Biology). Next, qPCR was performed using SYBR Green Premix Pro qPCR kit (Accurate Biology) on qPCR system (CFX OPUS 384, Bio-Rad). The following qPCR conditions were used: 40 cycles of denaturation at 95 °C for 10 s and annealing at 60 °C for 30 s. A comparative threshold cycle method was used to analyze the qPCR data, where the number of targets were normalized to the endogenous reference of GAPDH in each sample. The primer sequences of each gene are as follows:


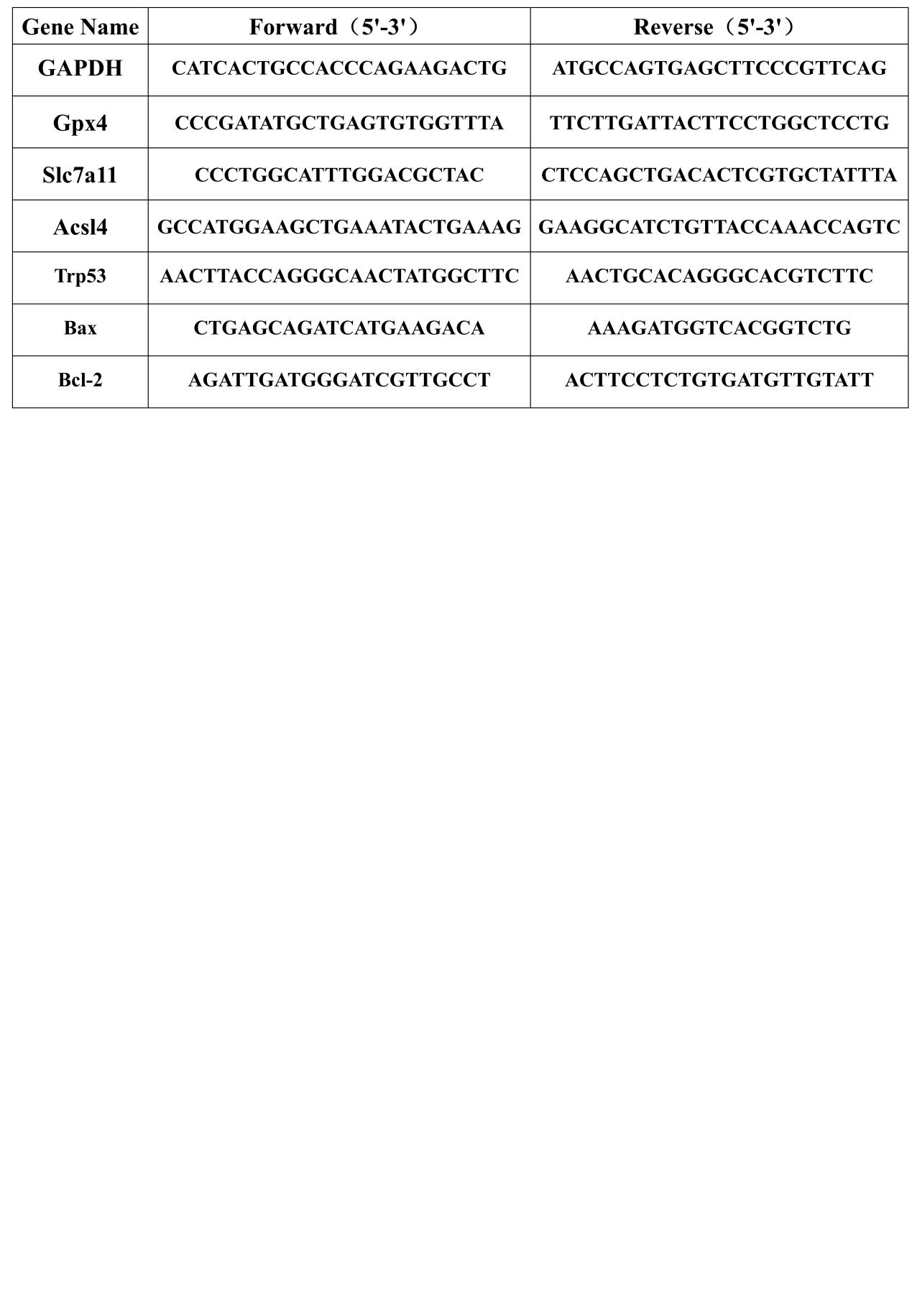


**Supplementary Figures**


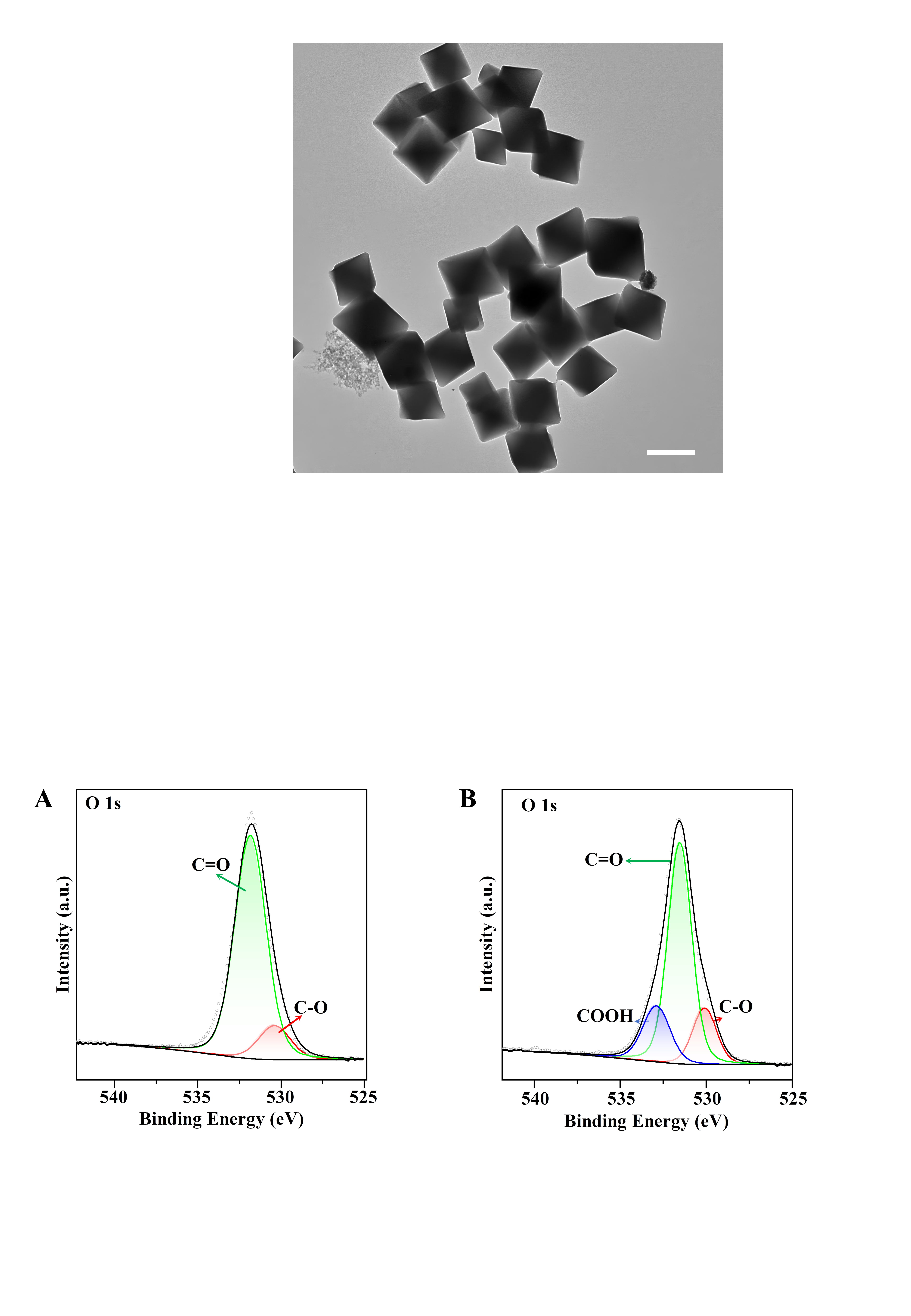


**Figure S1.** TEM image of pristine MOF-808-Hf. Scale bar = 200 nm.


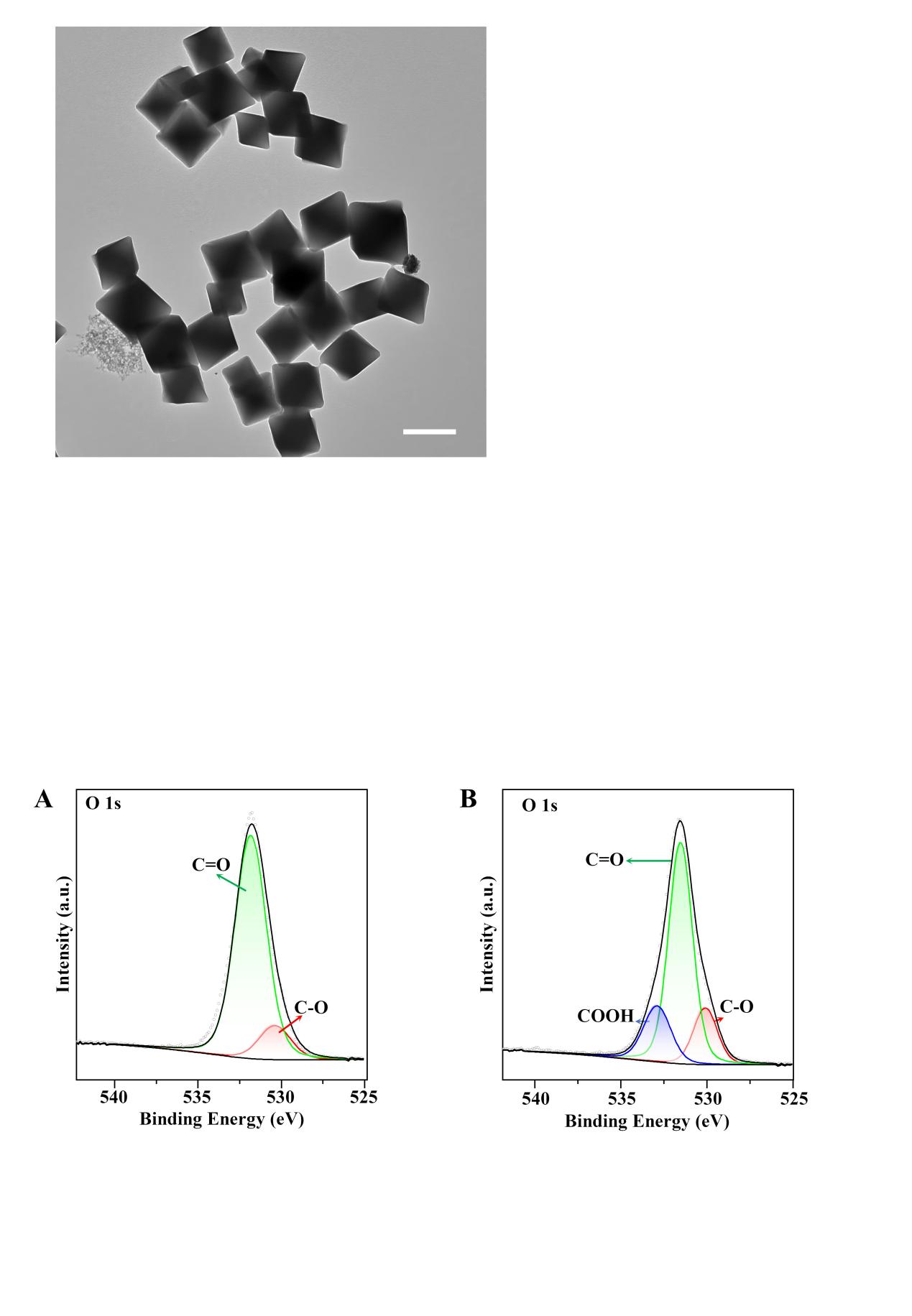


**Figure S2.** O 1s XPS spectra of MOF-808-Hf (A) and **ME** (B).


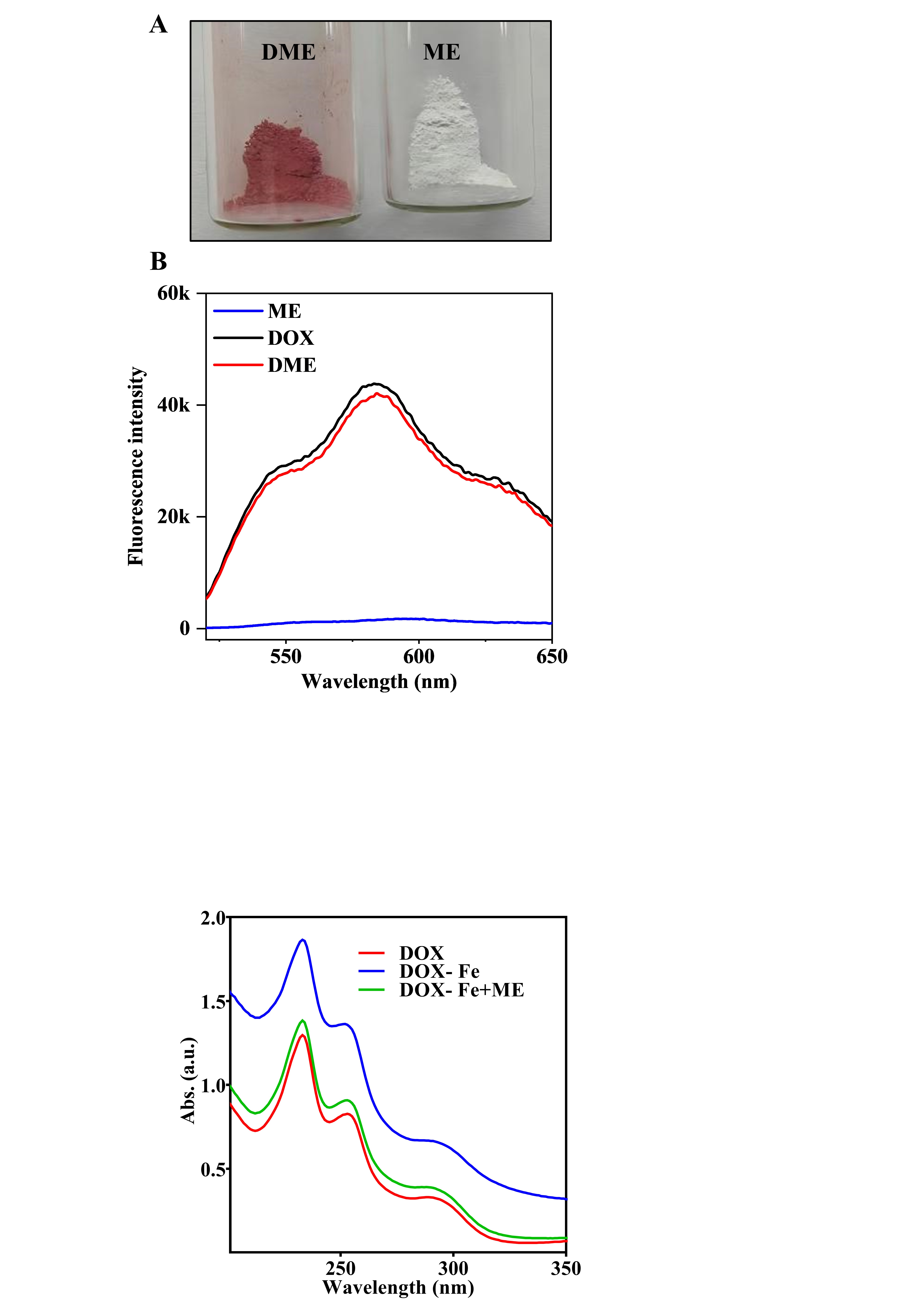


**Figure S3.** (A) Photos of **DME** and **ME**. (B) Fluorescence spectra of DOX, **ME** and **DME**.


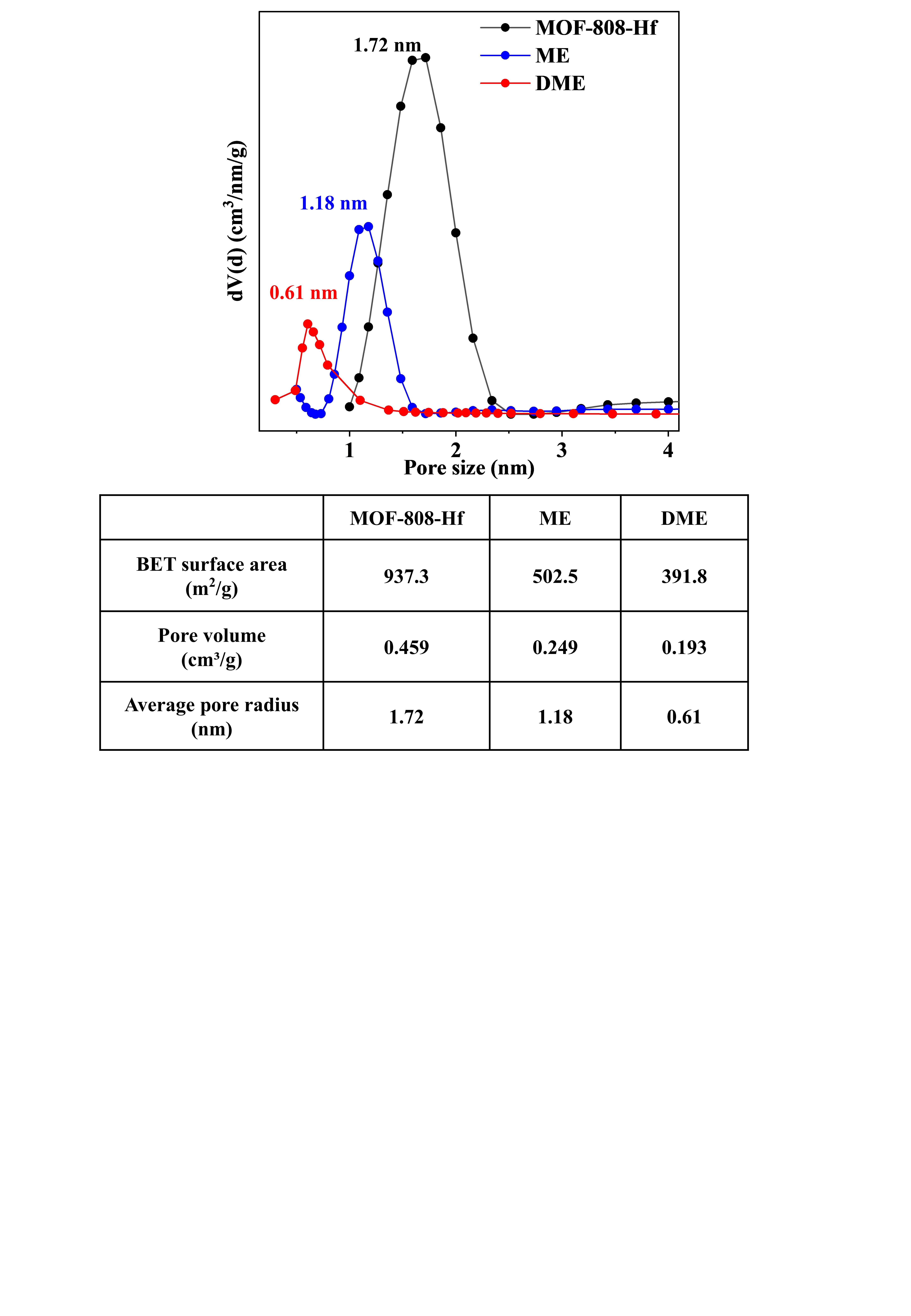


**Figure S4.** BET analysis of the pore size distribution of MOF-808-Hf, **ME** and **DME**.

**Figure S5.** UV-vis spectra of DOX, DOX-Fe complexes, and DOX-Fe after **ME** treatment.


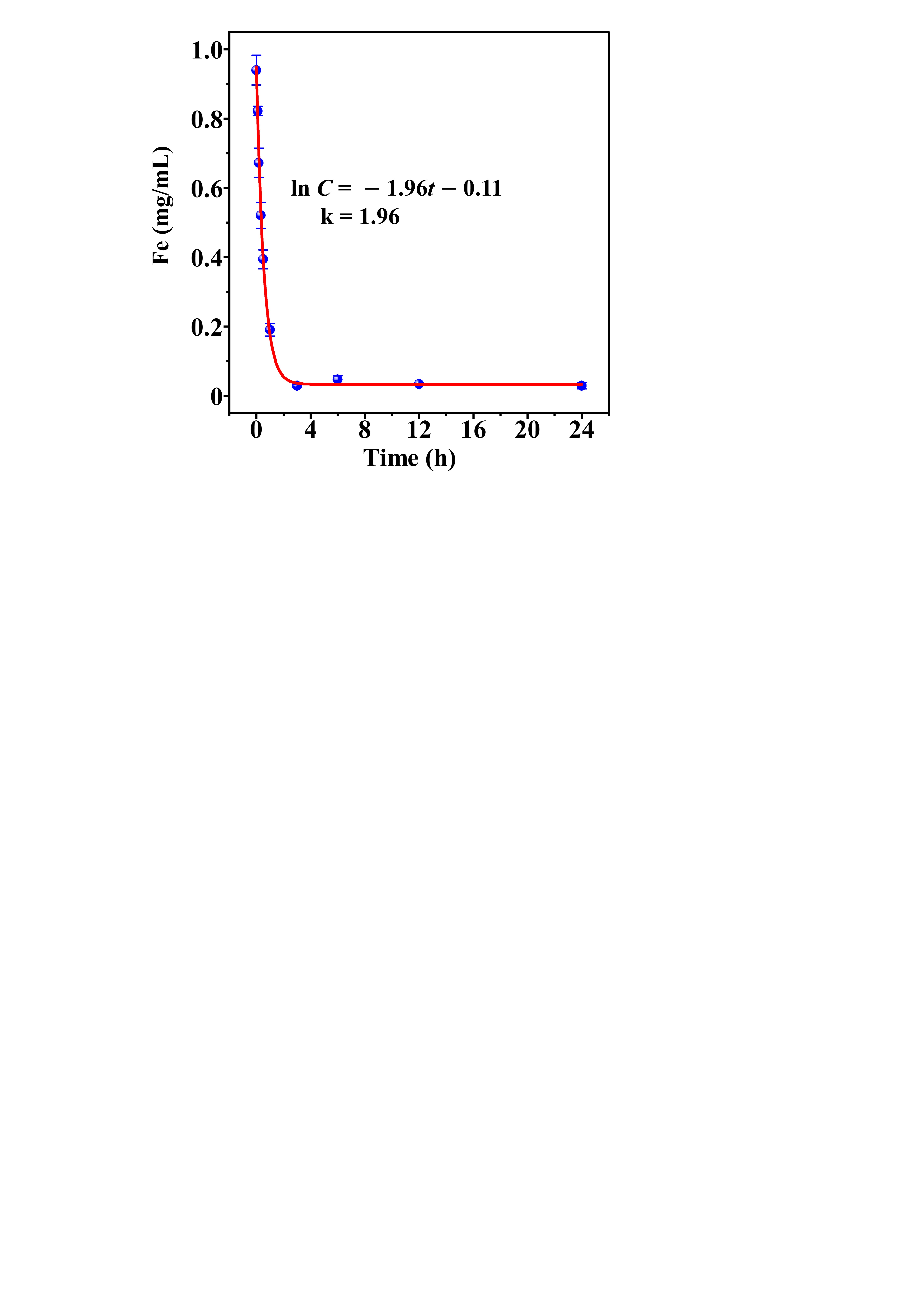


**Figure S6.** Pseudo-first-order reaction kinetics of Fe^2+^ removal from DOX-Fe complexes over time.


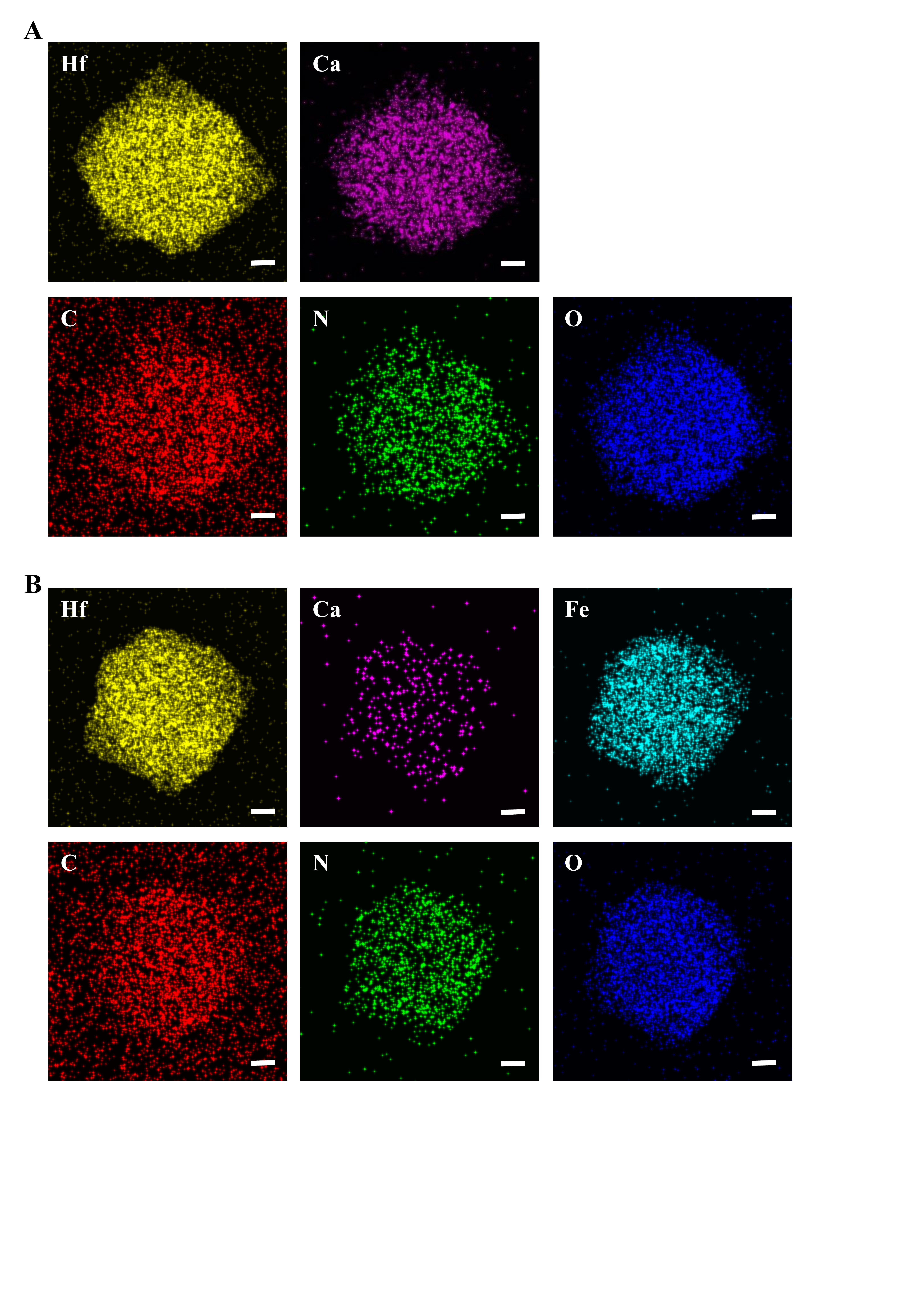


**Figure S7.** Elemental mapping of **Ca@ME** before (A) and after (B) FeCl_2_ treatment. Scale bar = 40 nm.


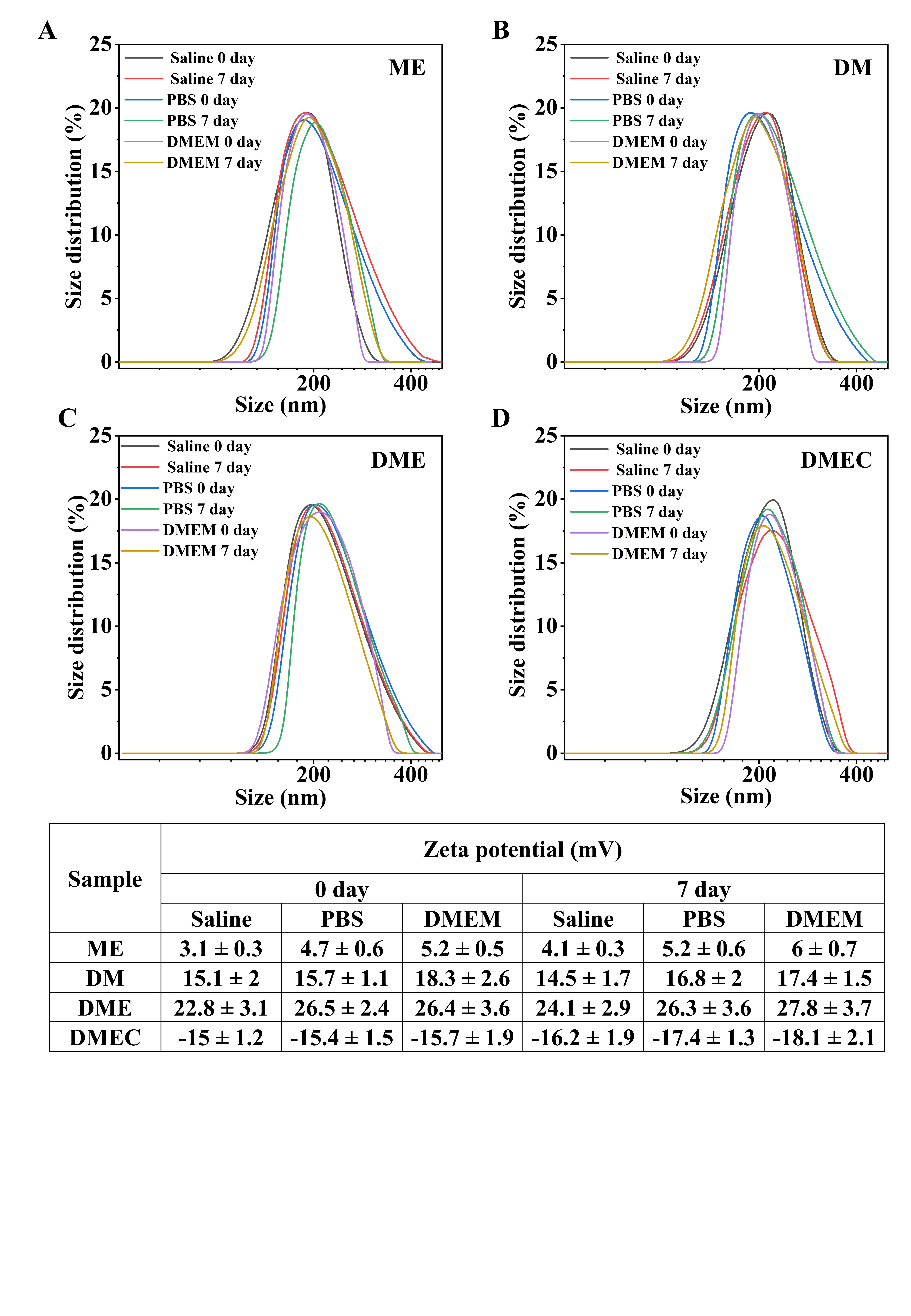


**Figure S8.** DLS analysis of **ME**, **DM**, **DME** and **DMEC** nanoparticles in different solutions over time.


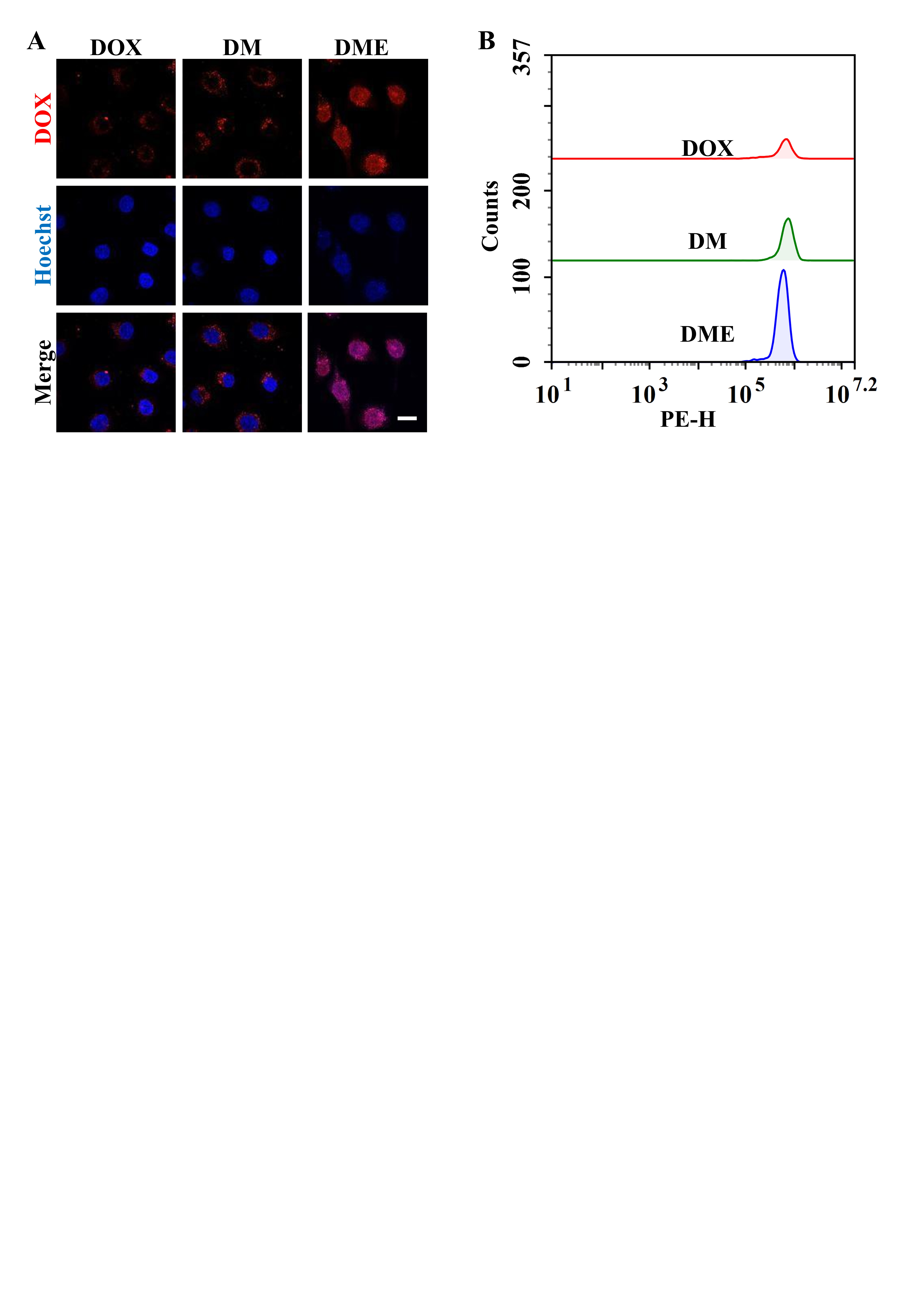


**Figure S9.** (A) CLSM images of MCF-7 cellular uptake of DOX, **DM** and **DME**. Scale bar = 20 μm. (B) Quantitative analysis of cellular uptake of DOX, **DM** and **DME** by flow cytometry.


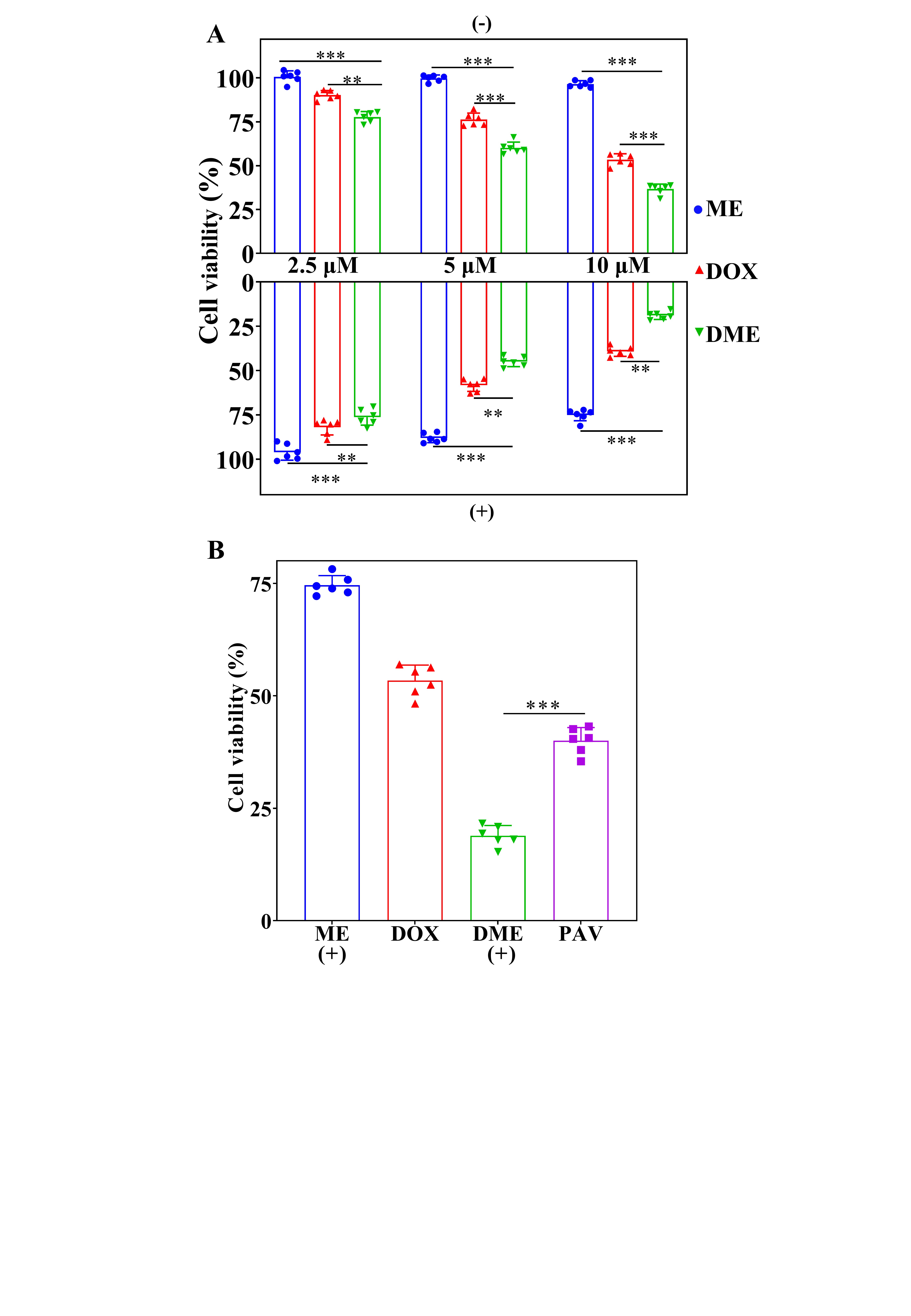


**Figure S10.** (A) MCF-7/ADR cell viability after incubated with **ME**, DOX and **DME** at various concentrations (2.5 μM, 5 μM, 10 μM based on DOX) with or without 4 Gy X-ray irradiation. (B) The projected additive value (PVA) of **DME**. The PVA is calculated by multiplying the cell viability of **ME (+)** and DOX group, + and − represent with or without 4 Gy X-ray irradiation. ***p* < 0.01, ****p* < 0.001.


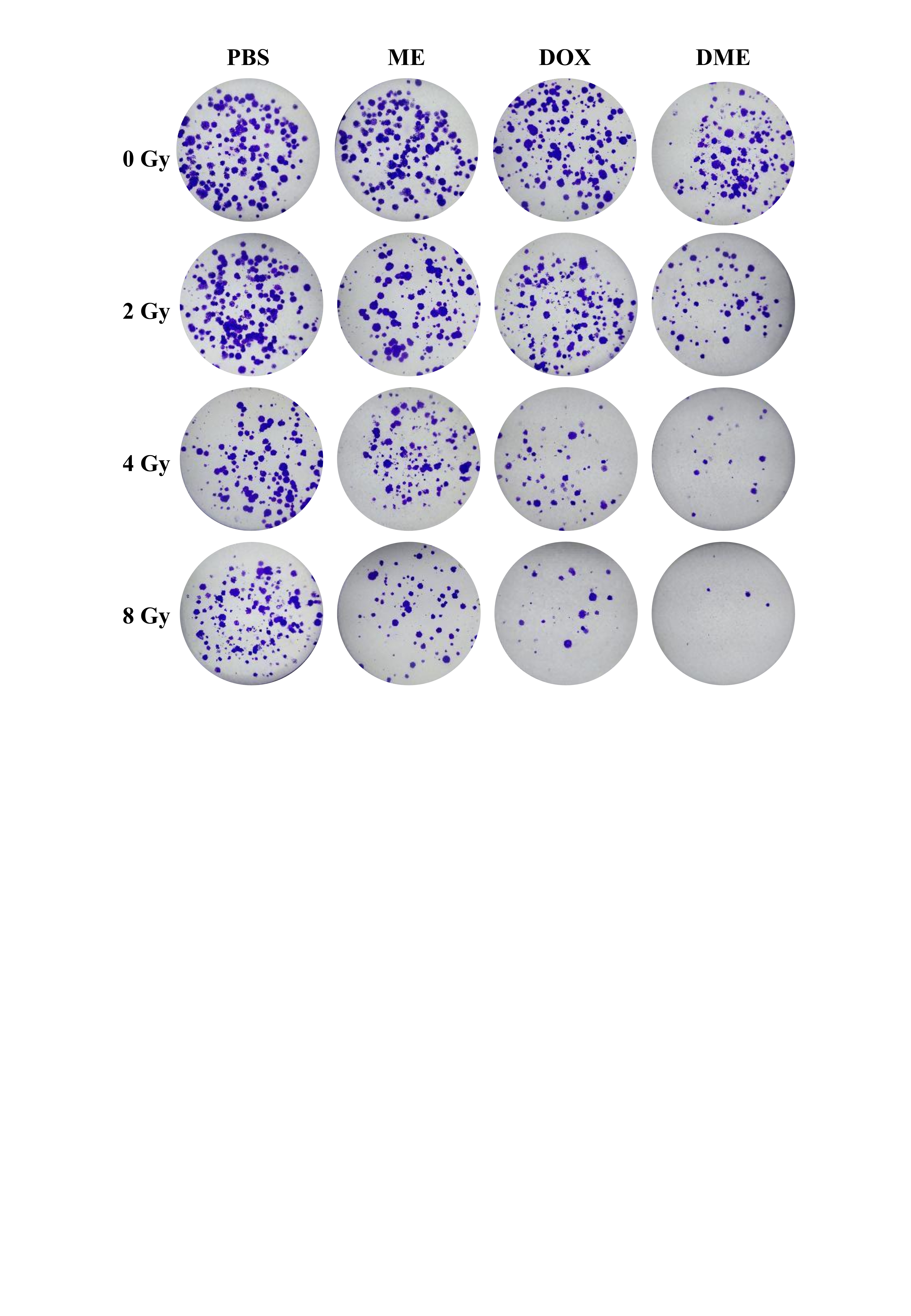


**Figure S11.** Colony formation assay of MCF-7 cells incubated with PBS, **ME**, DOX and **DME** (2 μM based on DOX) with X-ray (0 Gy, 2 Gy, 4 Gy, 8 Gy) irradiation.


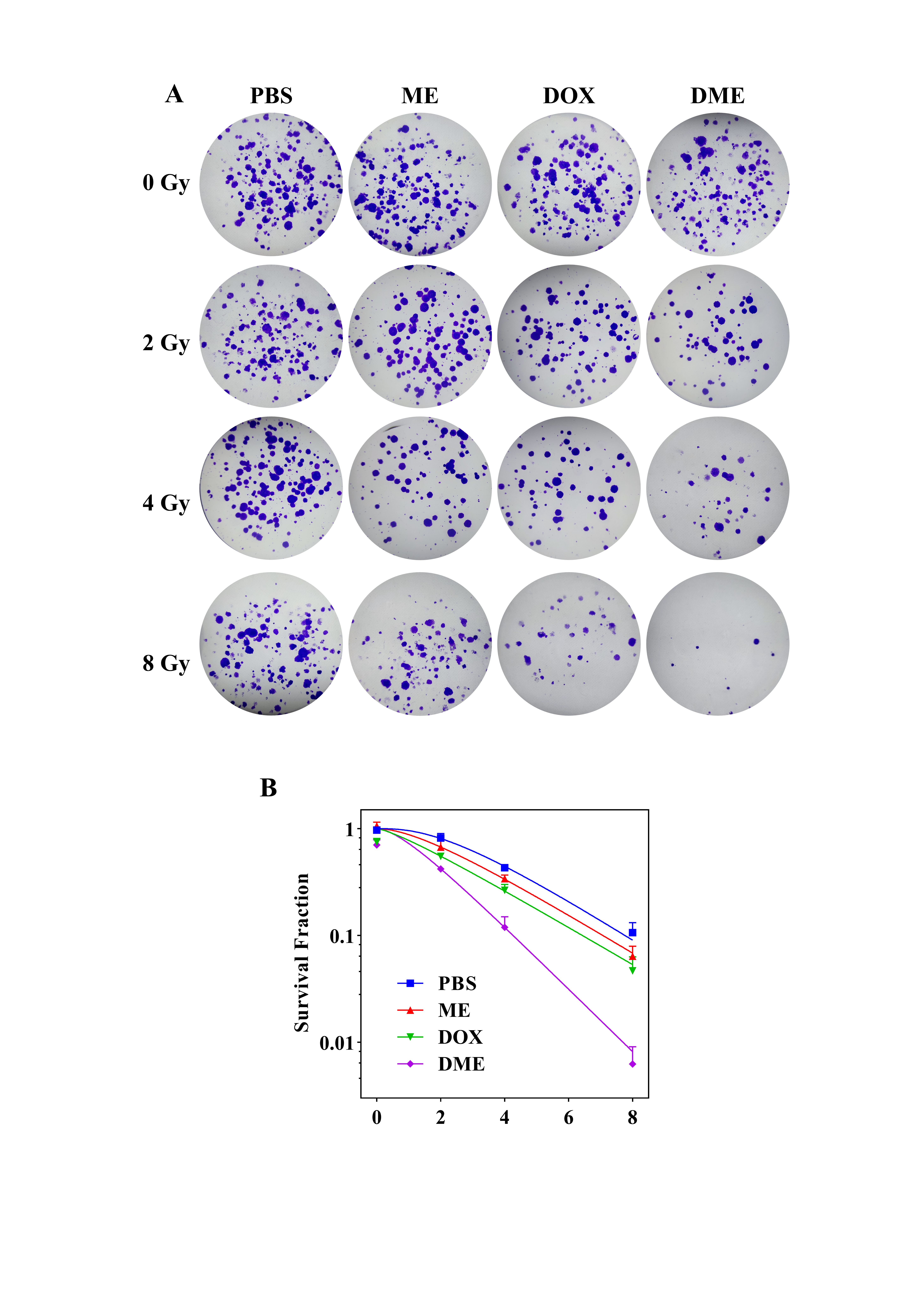


**Figure S12.** (A) Colony formation assay of MCF-7/ADR cells incubated with PBS, **ME**, DOX and **DME** (5 μM based on DOX) with X-ray (0 Gy, 2 Gy, 4 Gy, 8 Gy) irradiation. (B) The surviving fraction of MCF-7/ADR cells with various treatments.


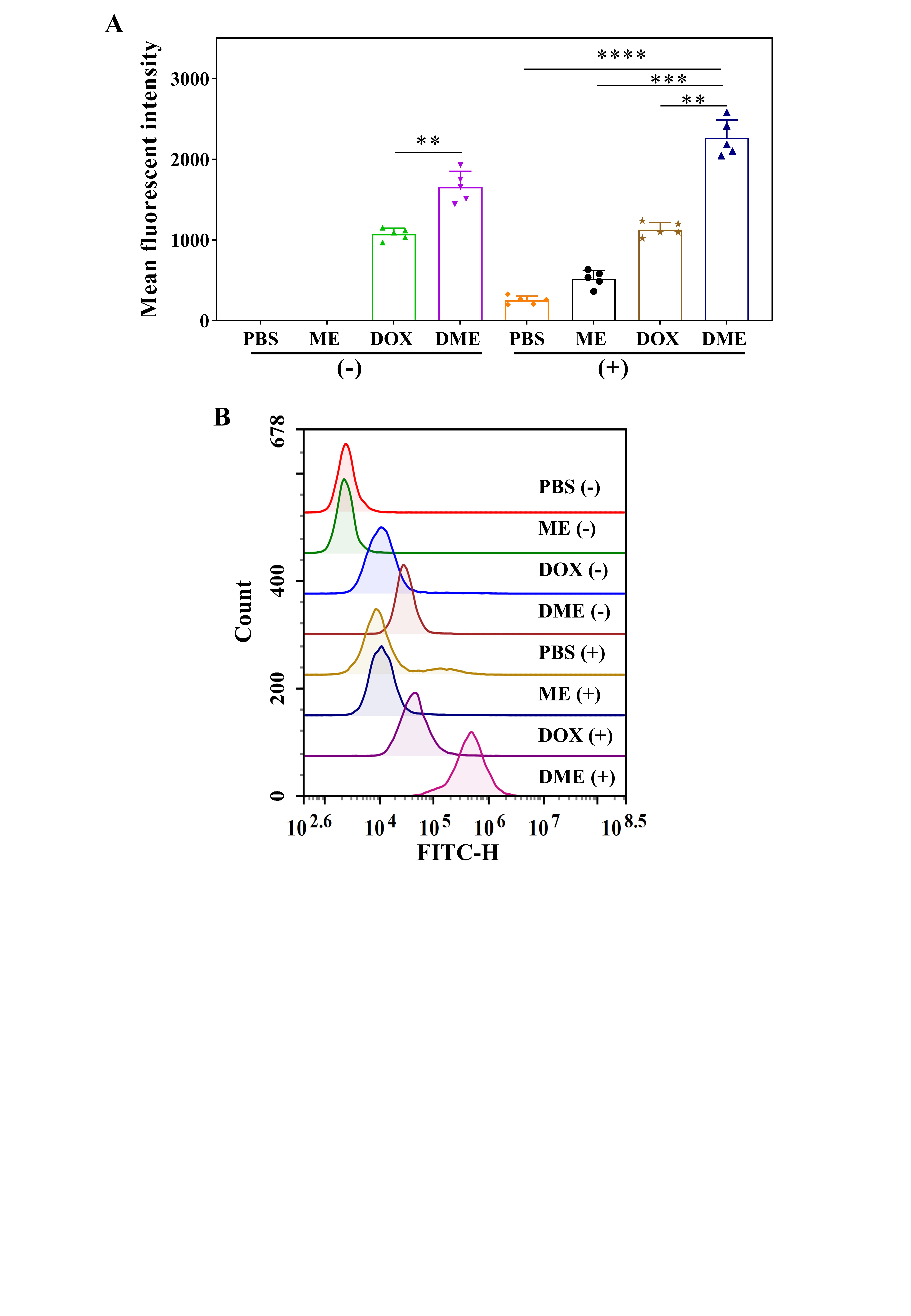


**Figure S13.** (A) Quantitative analyses of Figure 3F. (B) Flow cytometry analysis of •OH levels in MCF-7 cells after various treatments. + and − represent with or without 4 Gy X-ray irradiation. ***p* < 0.01, ****p* < 0.001, *****p* < 0.0001.


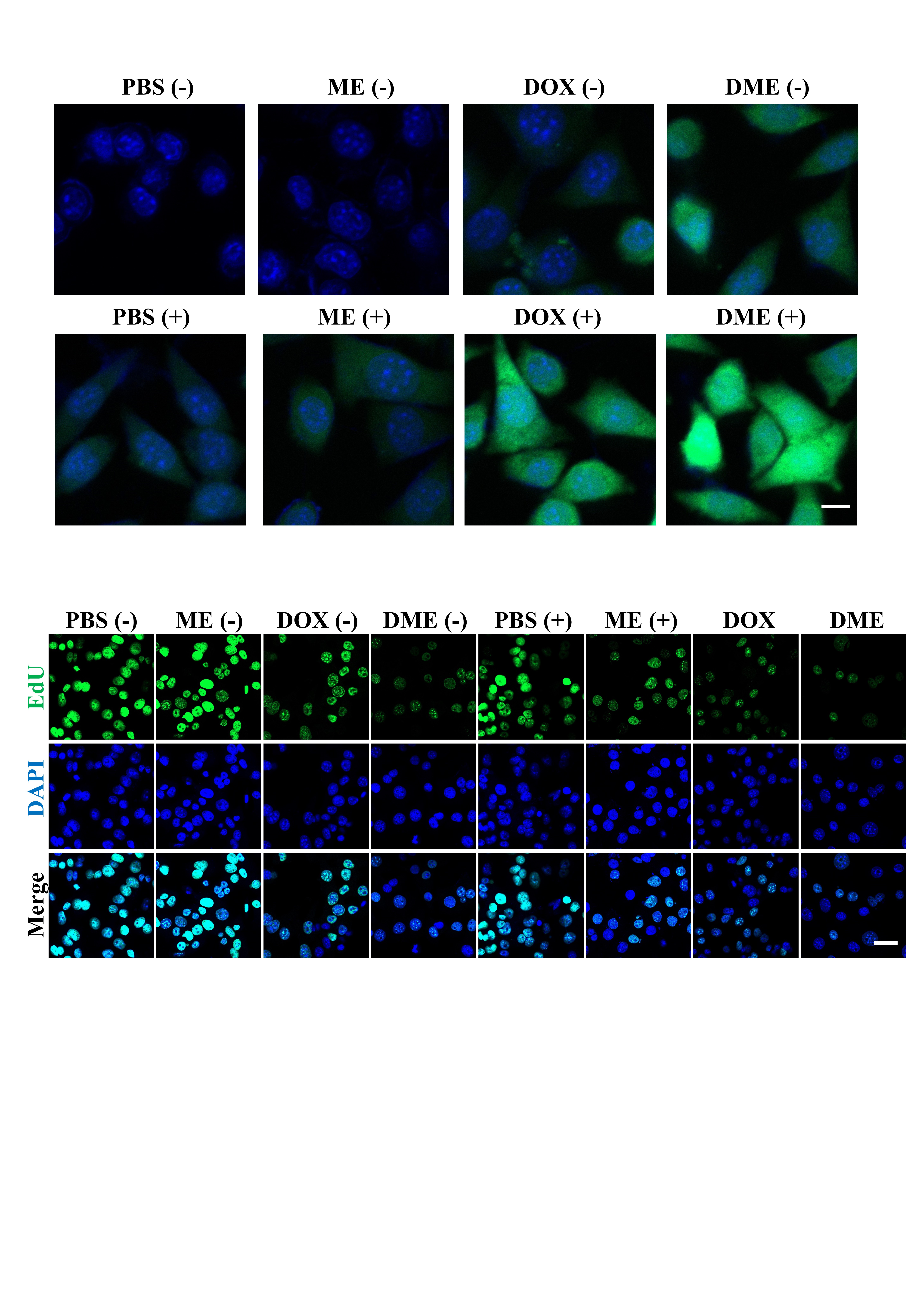


**Figure S14.** CLSM images of MCF-7 cells stained with DCFH-DA (green) and Hoechst (blue) after various treatments, + and − represent with or without 4 Gy X-ray irradiation. Scale bar = 10 µm.


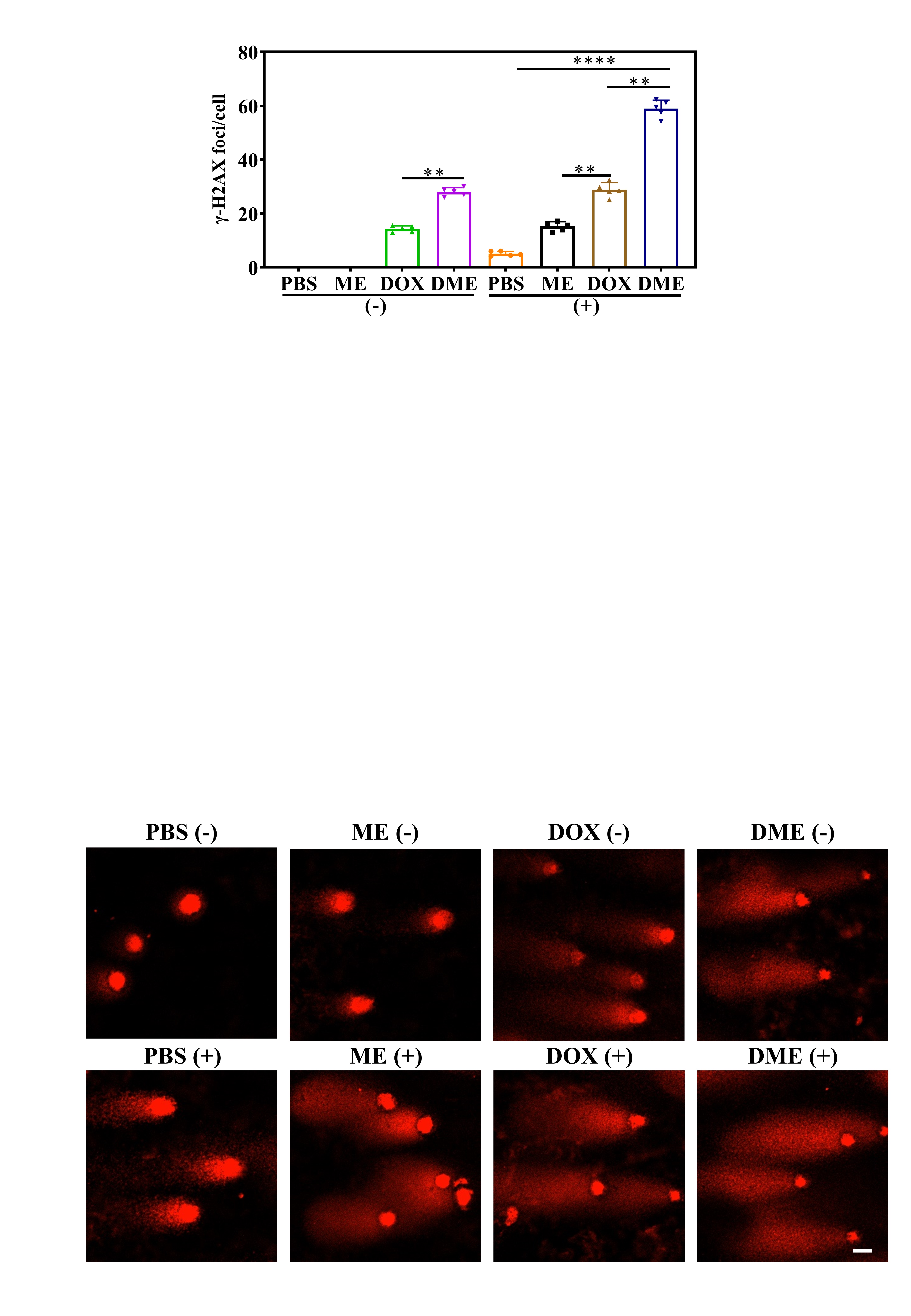


**Figure S15.** CLSM images of MCF-7 cells stained with comet assay (red) after various treatments, + and − represent with or without 4 Gy X-ray irradiation. Scale bar = 20 µm.


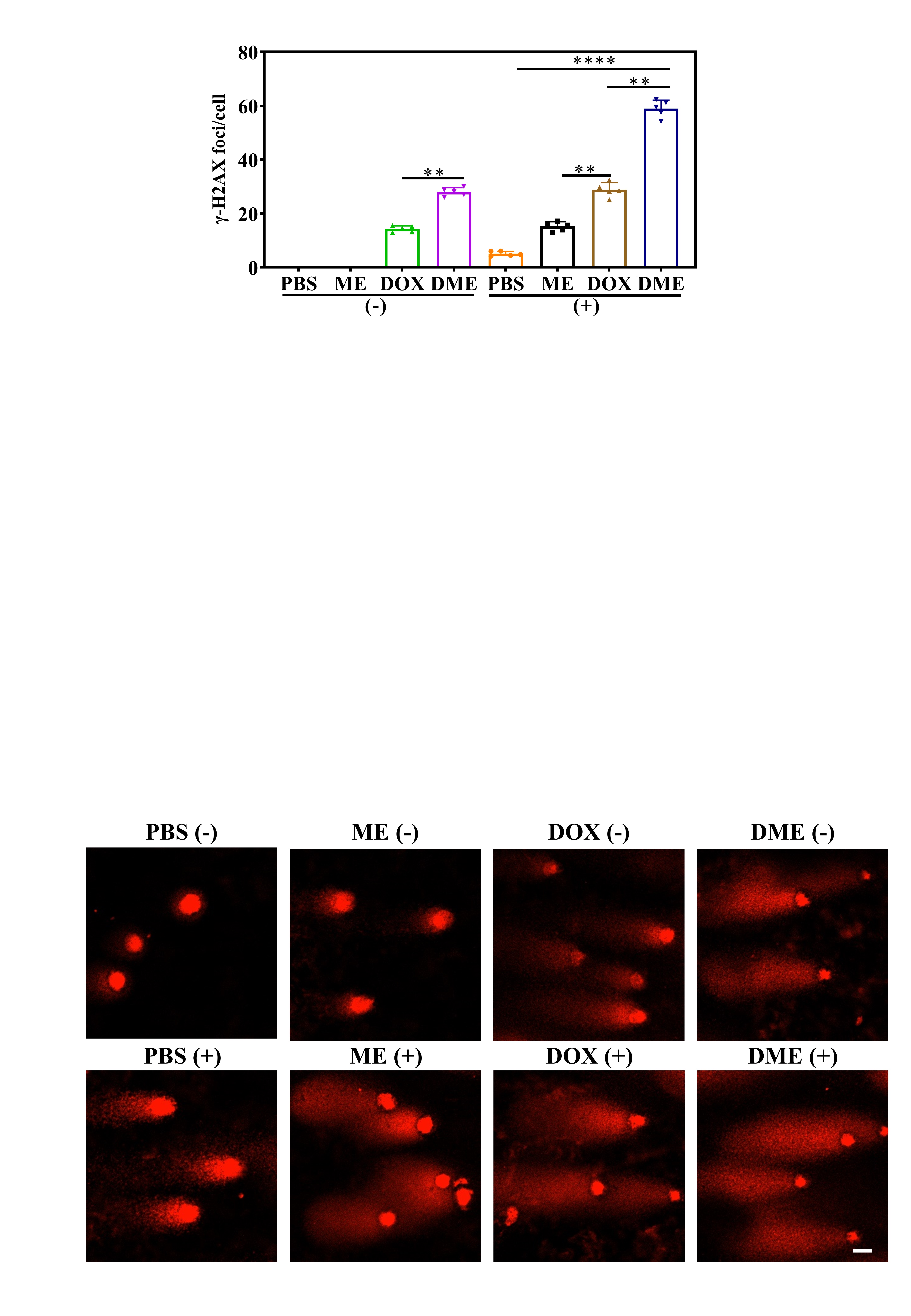


**Figure S16.** The number of red fluorescent foci of γ-H2AX with different treatments. + and − represent with or without 4 Gy X-ray irradiation. ***P* < 0.01 and ****P* < 0.001.


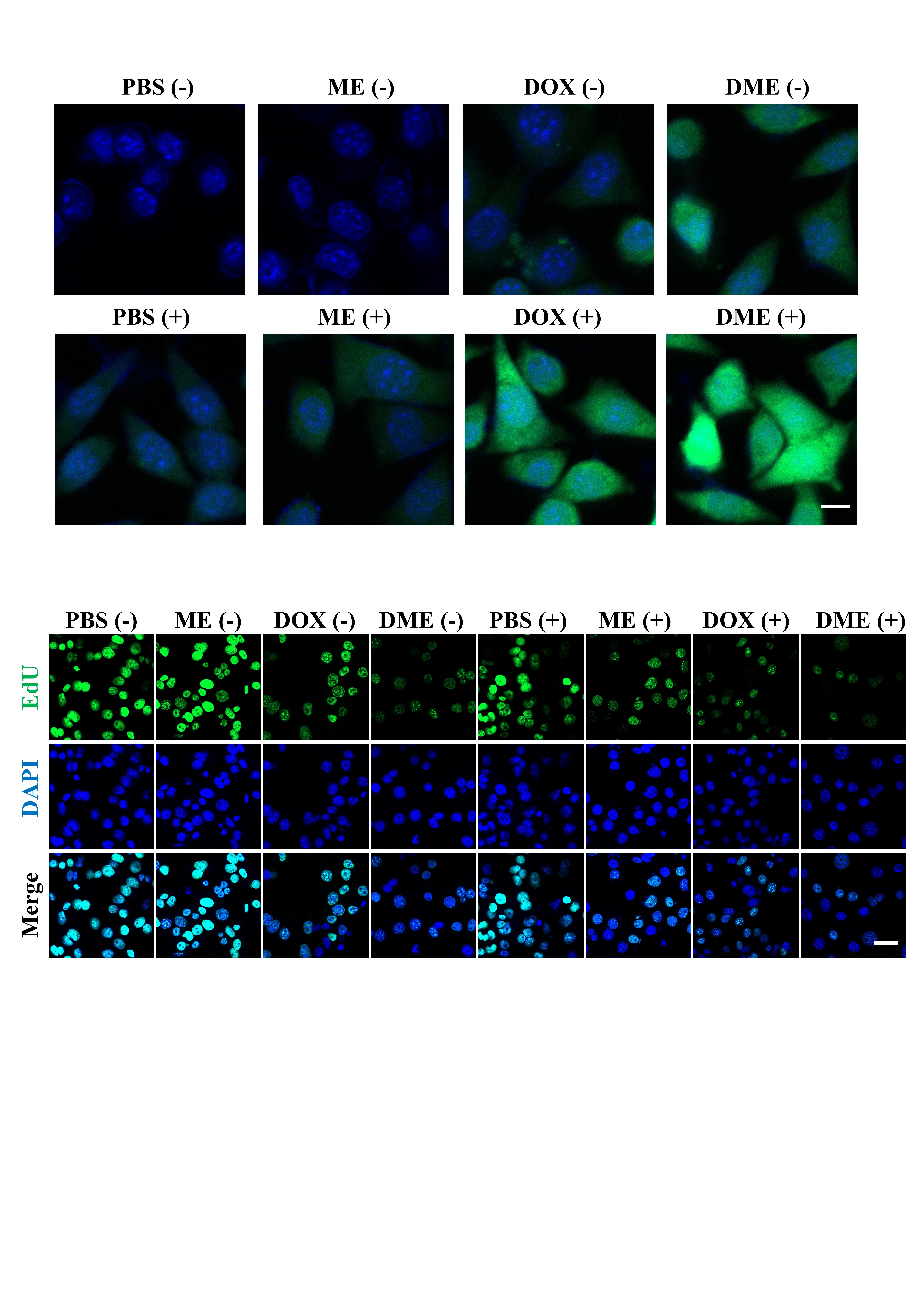


**Figure S17.** CLSM images of MCF-7 cells stained with EdU (green) and DAPI (blue) after various treatments, + and − represent with or without 4 Gy X-ray irradiation. Scale bar = 40 µm.


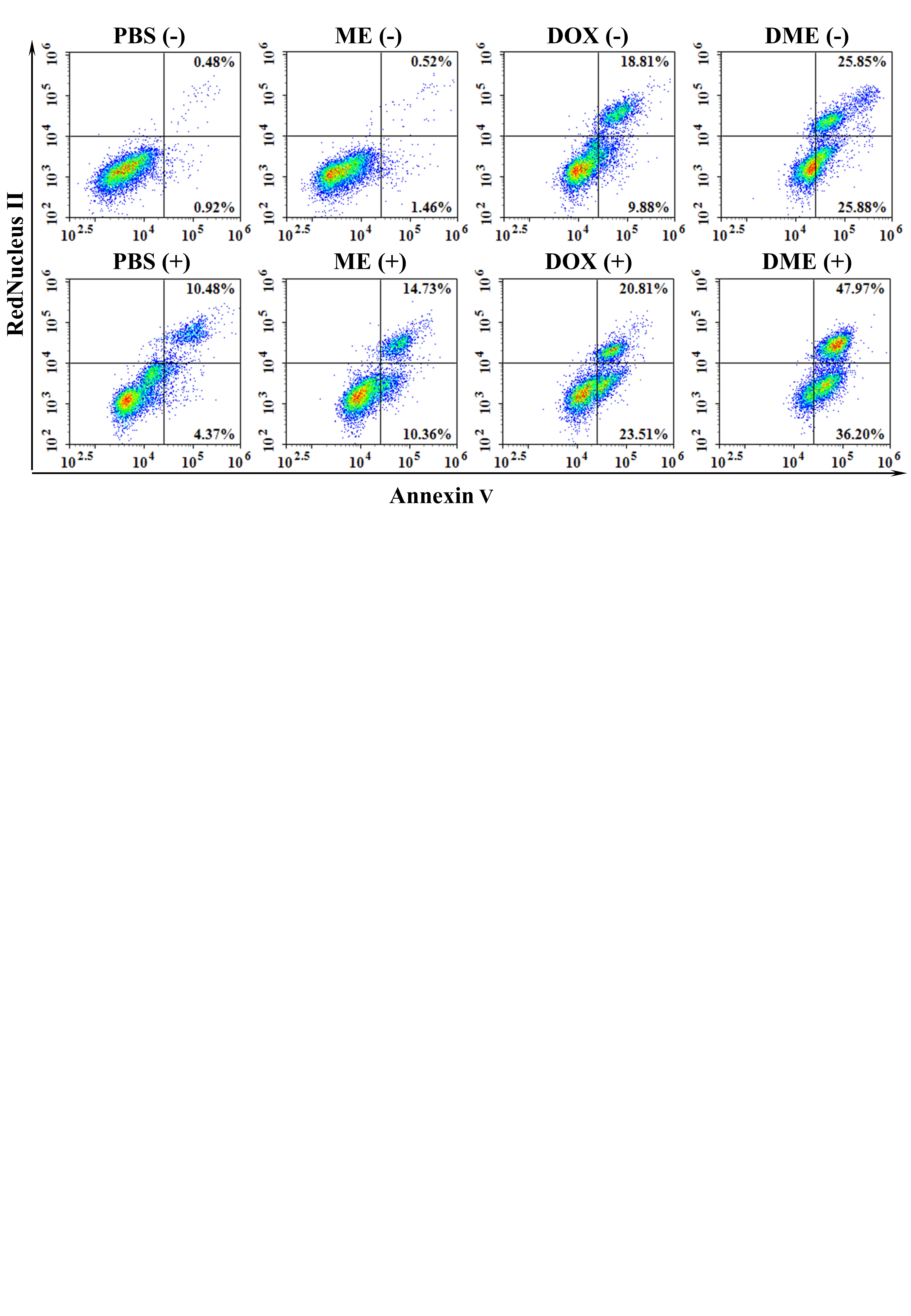


**Figure S18.** Flow cytometry analysis of apoptosis in MCF-7 cells after various treatments. + and − represent with or without 4 Gy X-ray irradiation, respectively.


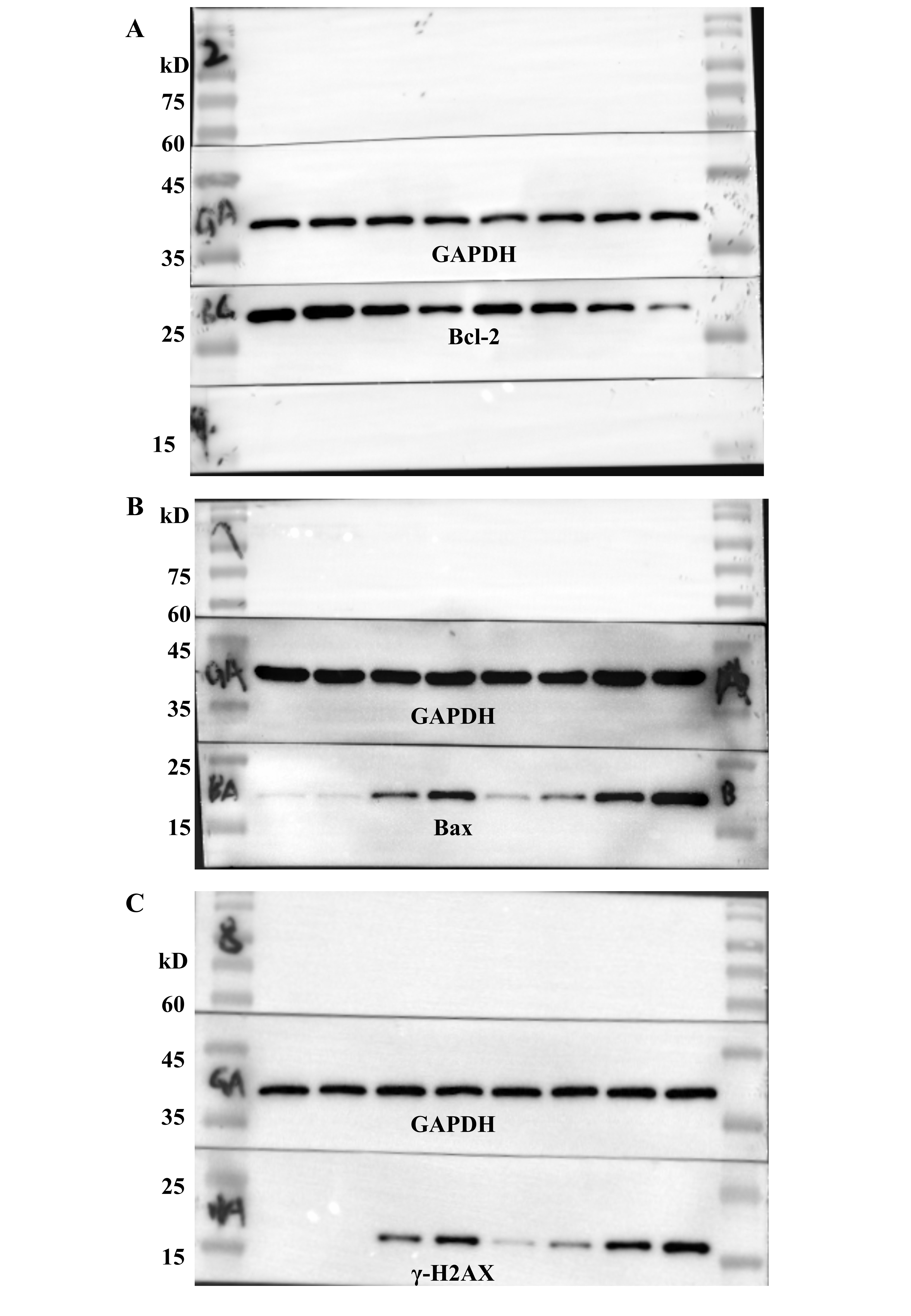


**Figure S19.** The full Western blot analysis of Bcl-2 (A), Bax (B) and γ-H2AX (C) proteins in MCF-7 cells after various treatments.


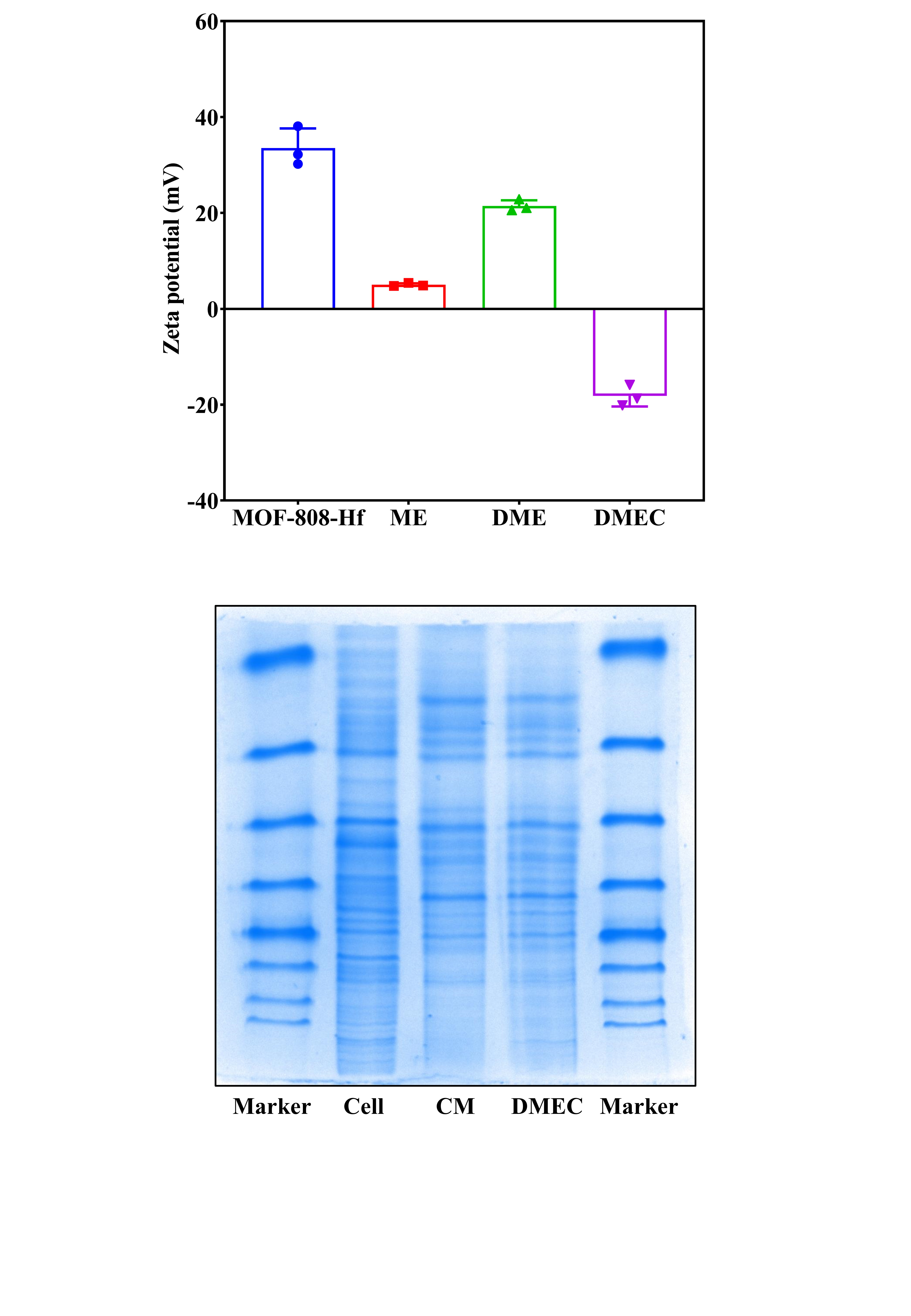


**Figure S20.** Zeta potentials of MOF-808-Hf, **ME**, **DME** and **DMEC**.


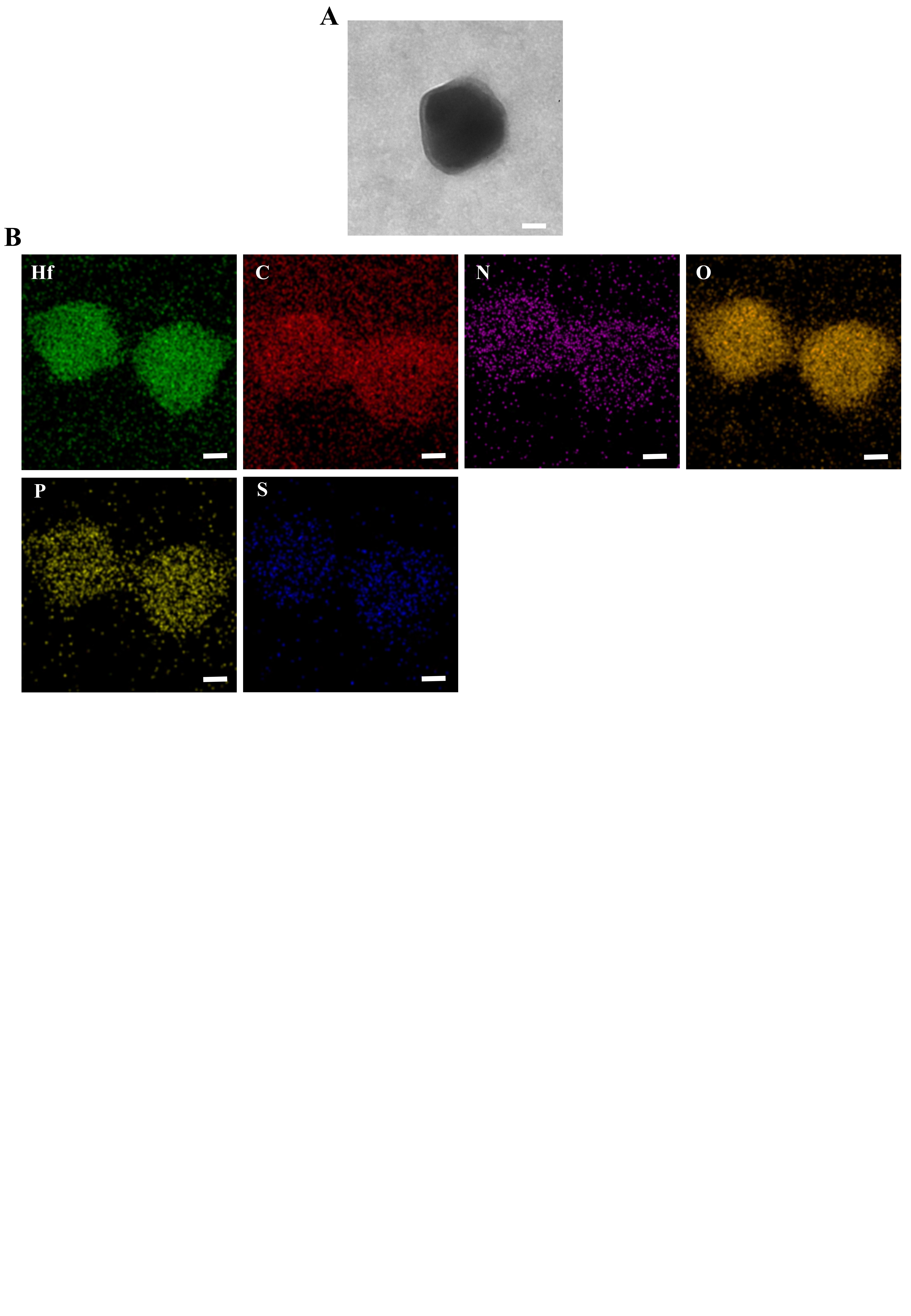


**Figure S21.** (A) TEM image of **DMEC**. Scale bar = 50 nm. (B) Element mapping of **DMEC**. Scale bar = 50 nm.


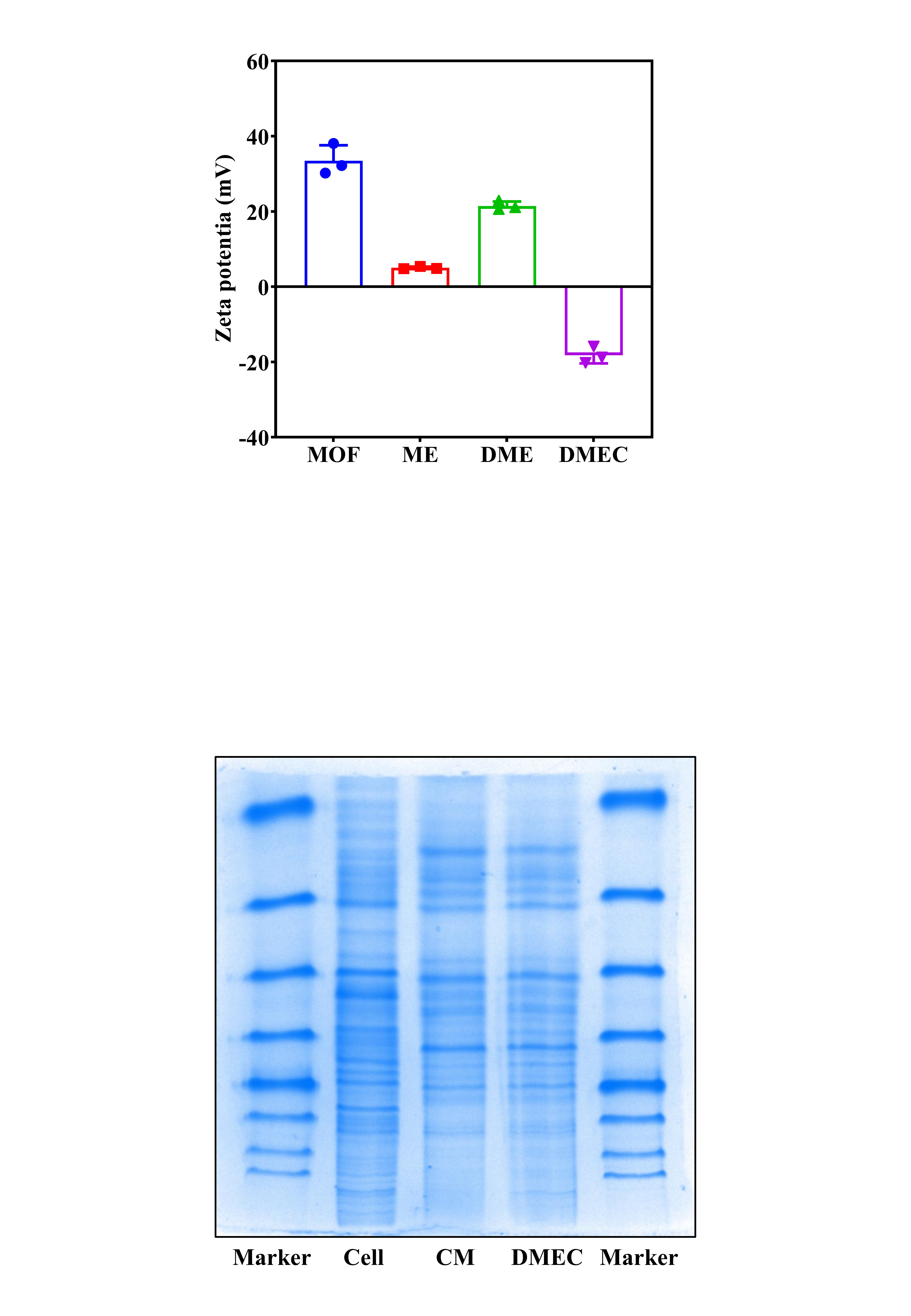


**Figure S22.** Protein gel electrophoresis image of MCF-7 cell, membrane of MCF-7 cell (CM) and **DMEC**.


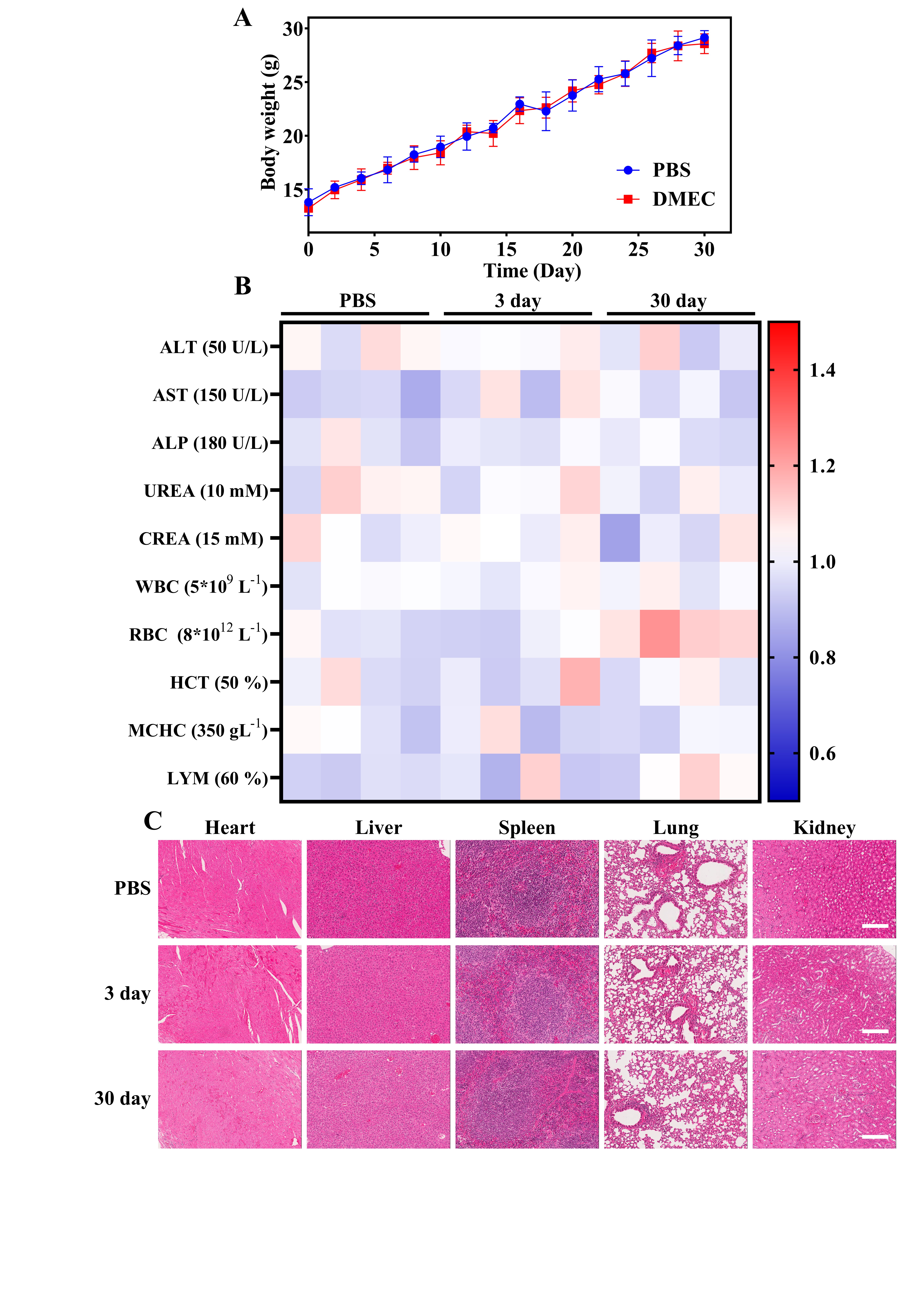


**Figure S23.** (A) Body-weight curves of the mice treated by PBS and **DMEC**. (B) Serological biochemical indicators on the PBS or **DMEC** treatments. (C) H&E staining images of mice primary organs (heart, liver, spleen, lung, and kidney) on the PBS or **DMEC** treatments. The tissue sections were harvested in 3 and 30 days. Scale bar = 100 µm.


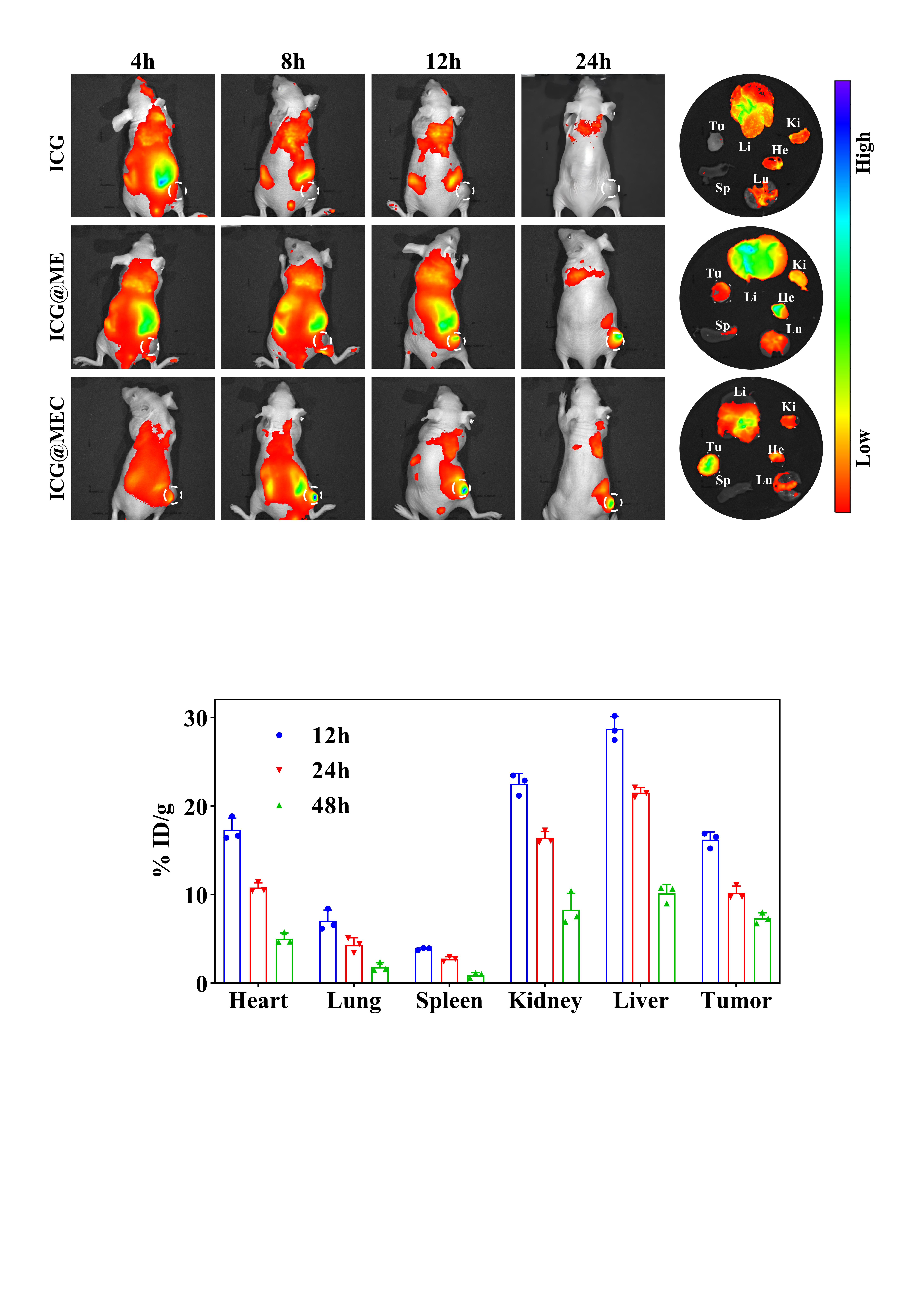


**Figure S24.** Small animal fluorescence imaging system monitors tissue distribution of the ICG, ICG@**ME** and ICG@**MEC**. Li: liver, He: heart, Lu: lung, Ki: kidney, Sp: spleen and Tu: tumor.


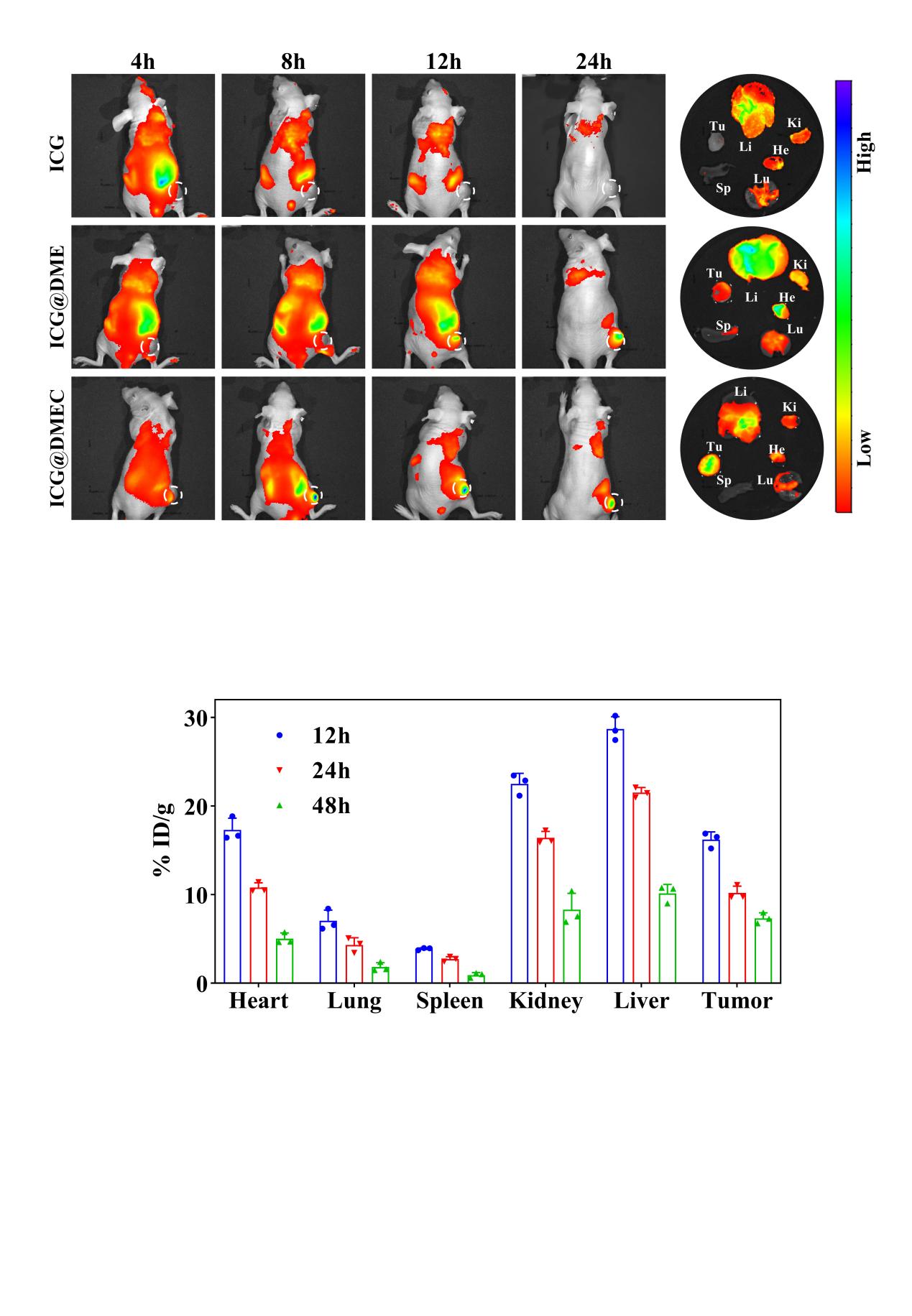


**Figure S25.** The biodistribution of **DMEC** in different organs and tumors at various times.


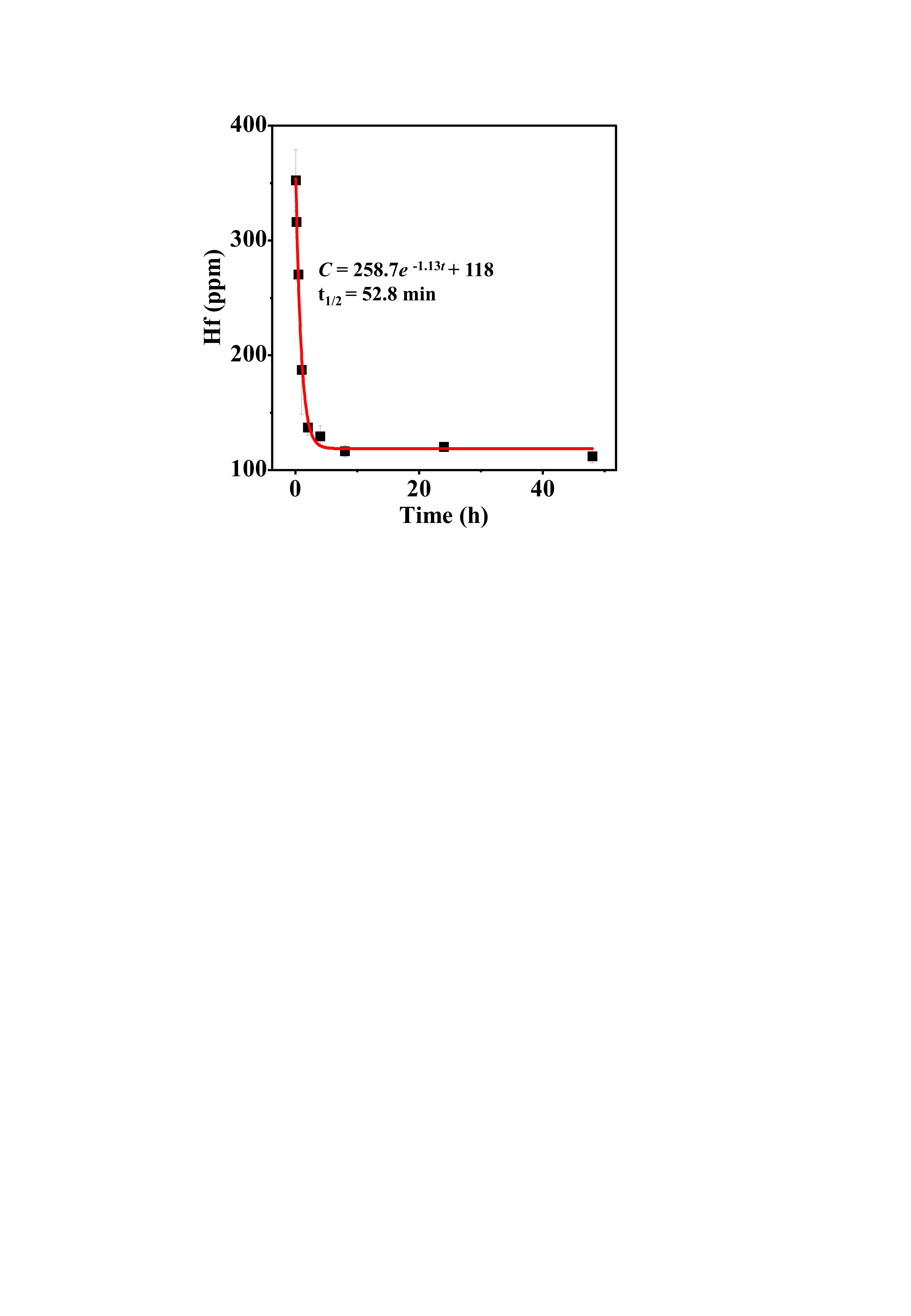


**Figure S26.** Time-dependent blood circulation of **DMEC**. The blood circulation half-time of **DMEC** was calculated to be about 52.8 min based on the one-component pharmacokinetic model.


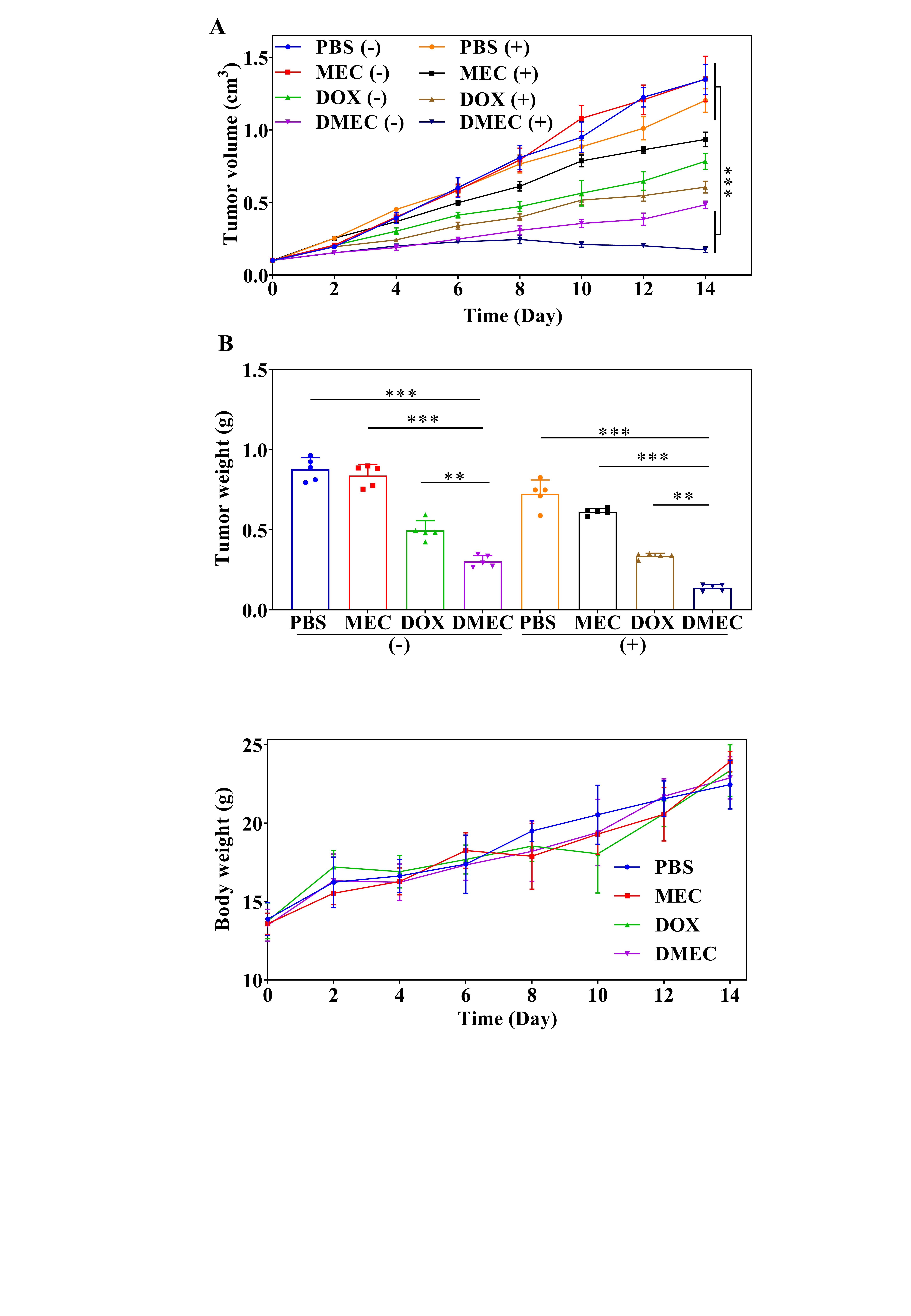


**Figure S27.** (A) Tumor volume and (B) tumor weight of MCF-7 xenografted mice after different treatments, + and − represent with or without X-ray irradiation. ***p* < 0.01, ****p* < 0.001.


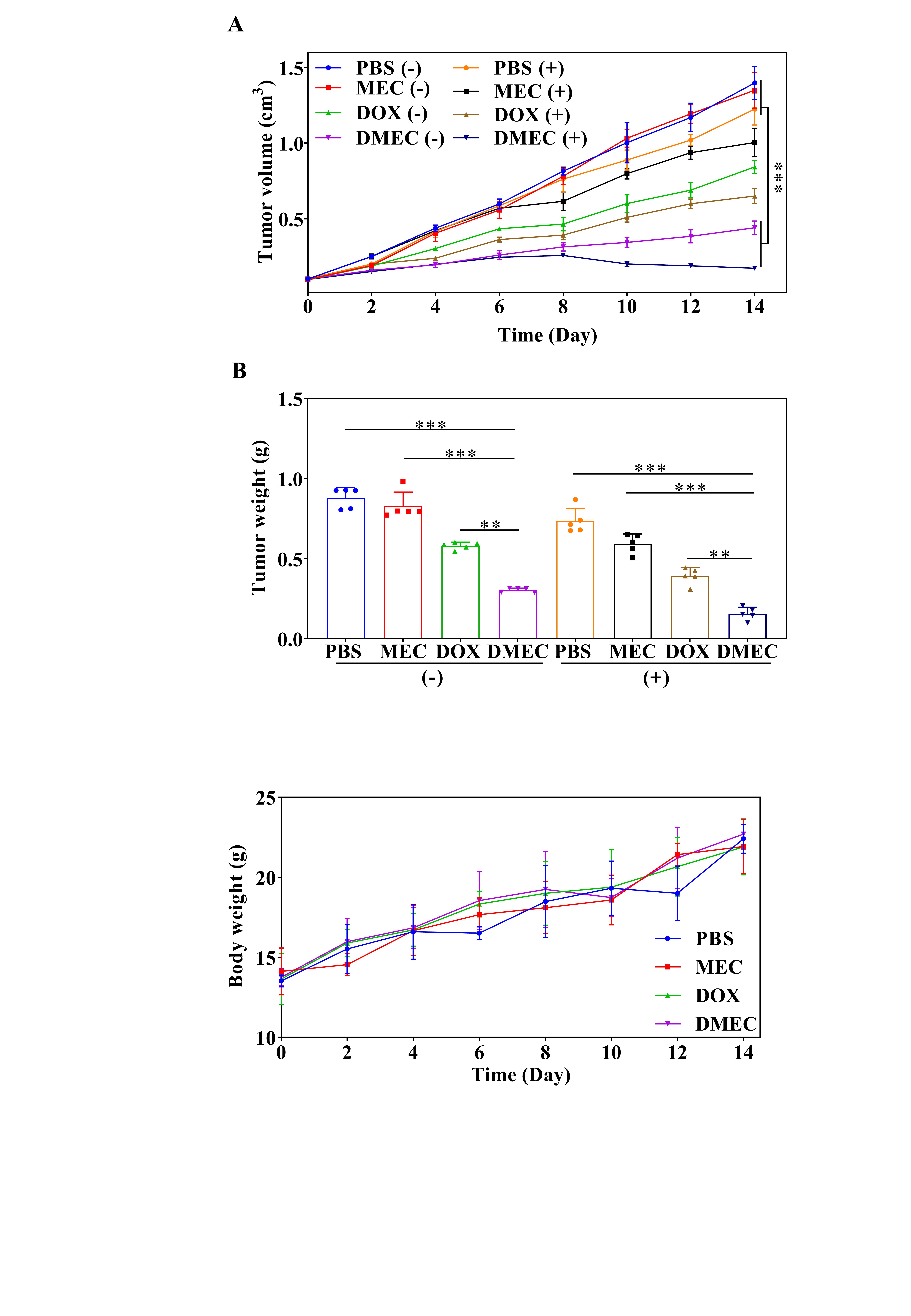


**Figure S28.** (A) Tumor volume and (B) tumor weight of MCF-7/ADR xenografted mice after different treatments, + and − represent with or without X-ray irradiation. ***p* < 0.01, ****p* < 0.001.


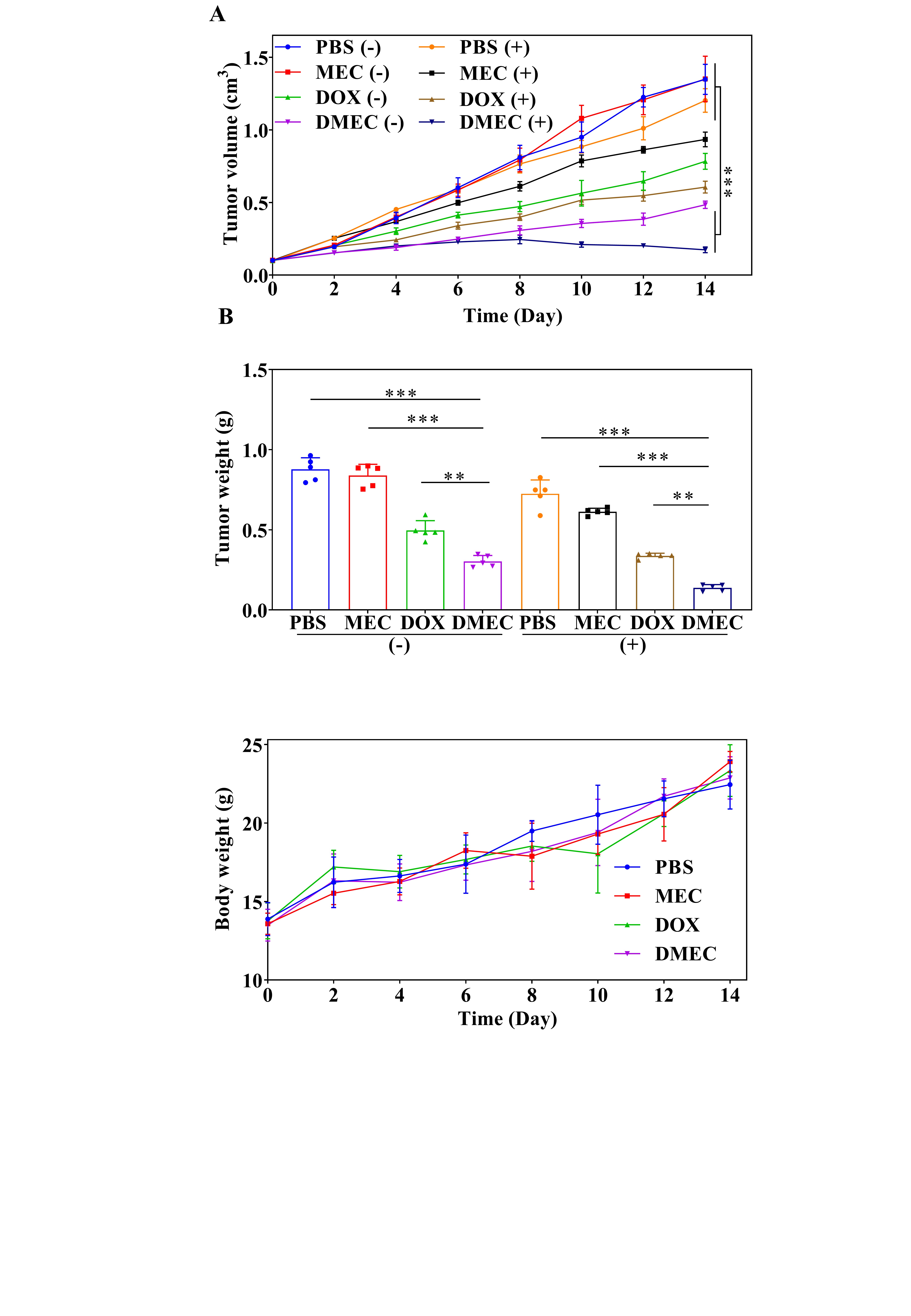


**Figure S29.** Body weight of MCF-7 tumor-bearing mice during the treatment.


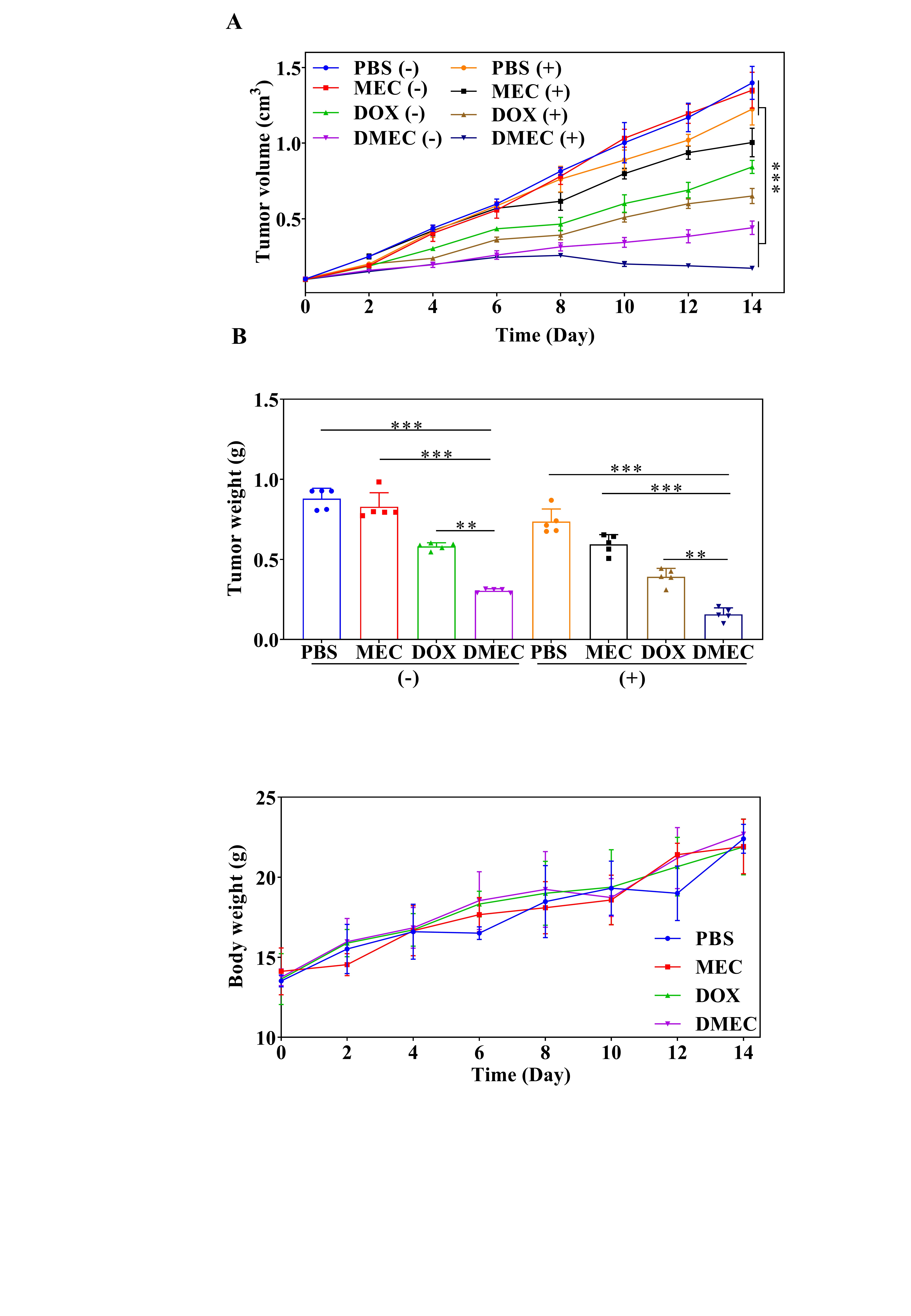


**Figure S30.** Body weight of MCF-7/ADR tumor-bearing mice during the treatment.


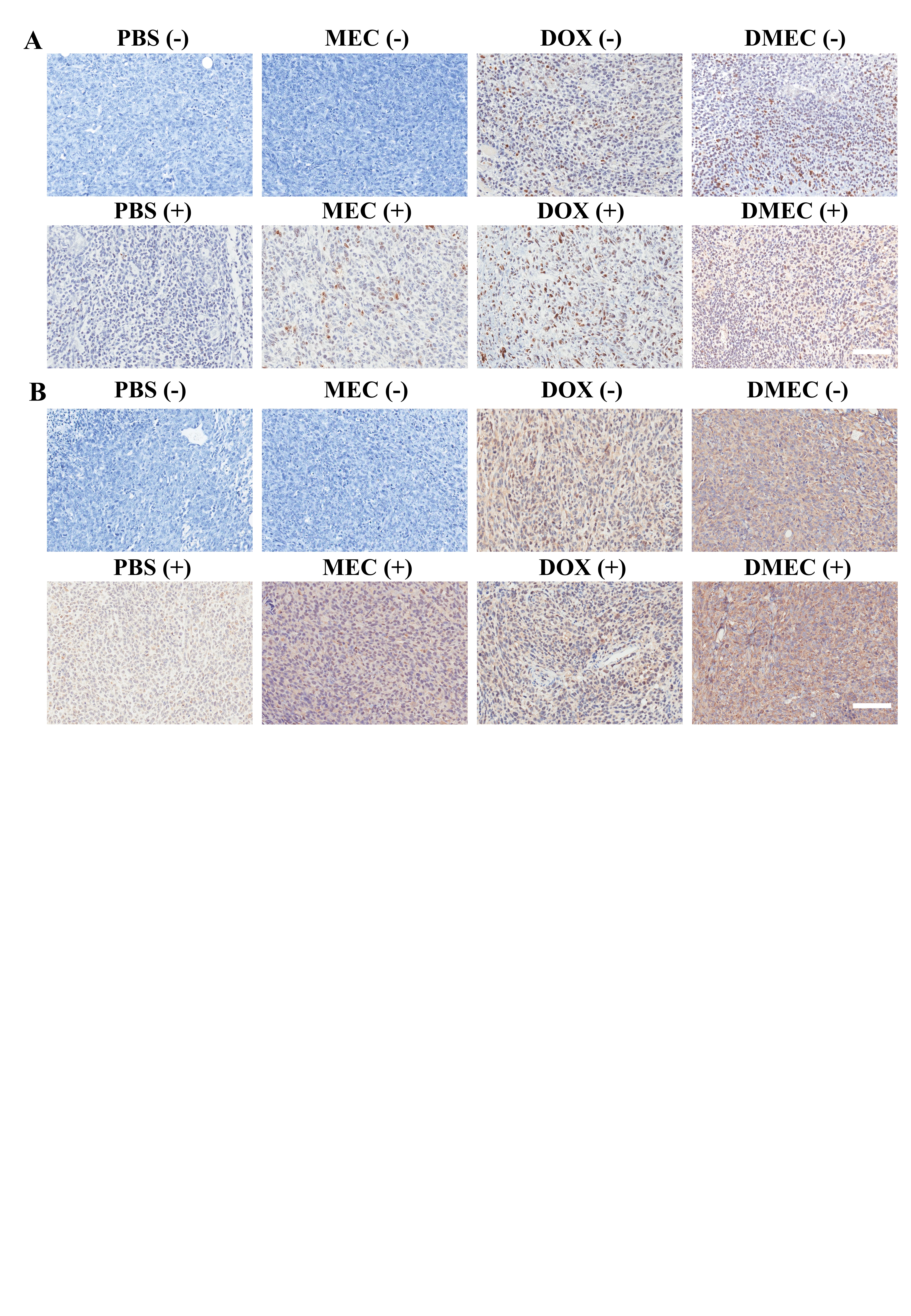


**Figure S31.** γ-H2AX (A) and Bax (B) immunohistochemical staining images of the MCF-7 tumor tissues after different treatments, + and − represent with or without X-ray irradiation. Scale bar = 100 μm.


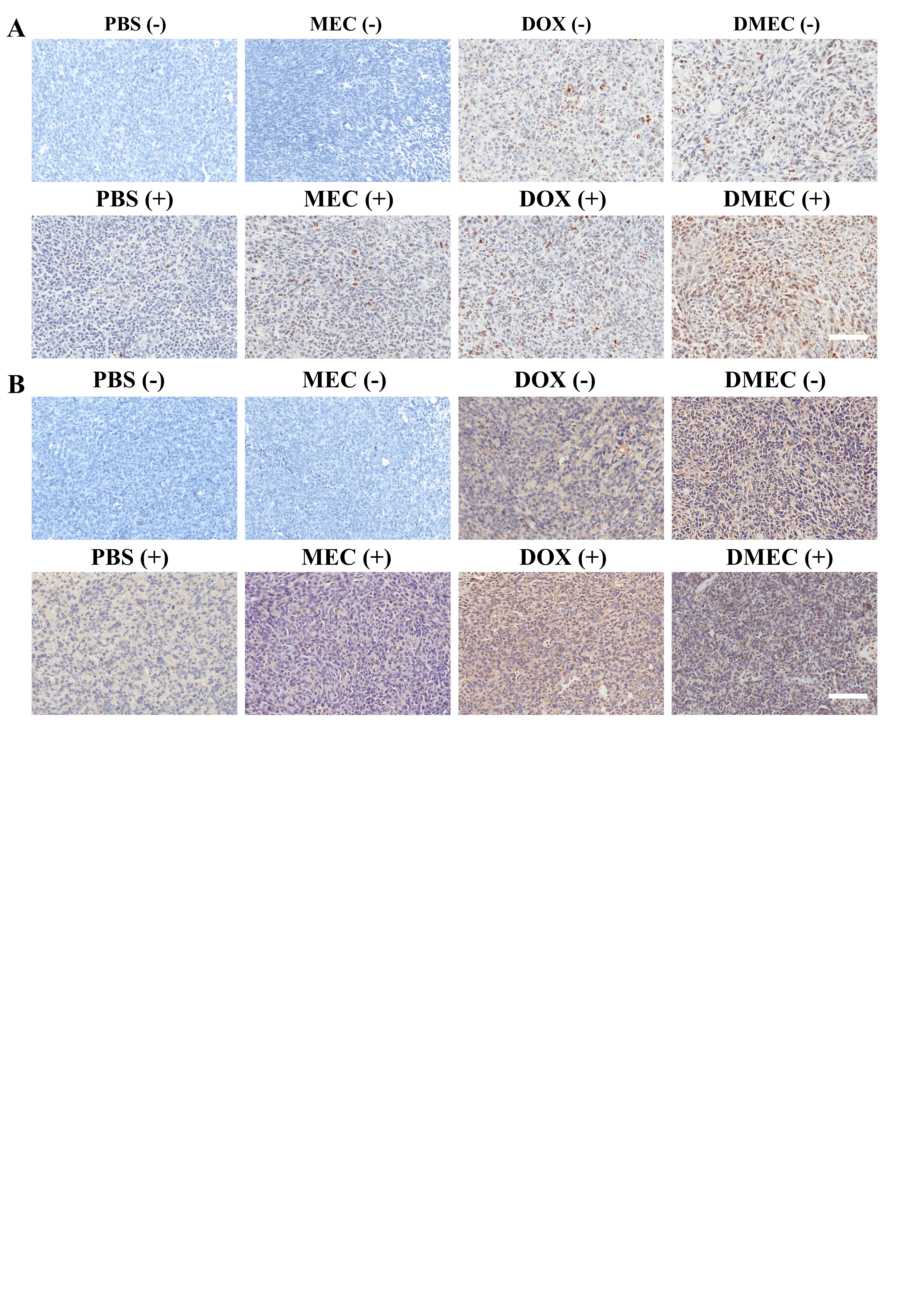


**Figure S32.** γ-H2AX (A) and Bax (B) immunohistochemical staining images of the MCF-7/ADR tumor tissues after different treatments, + and − represent with or without X-ray irradiation. Scale bar = 100 μm.


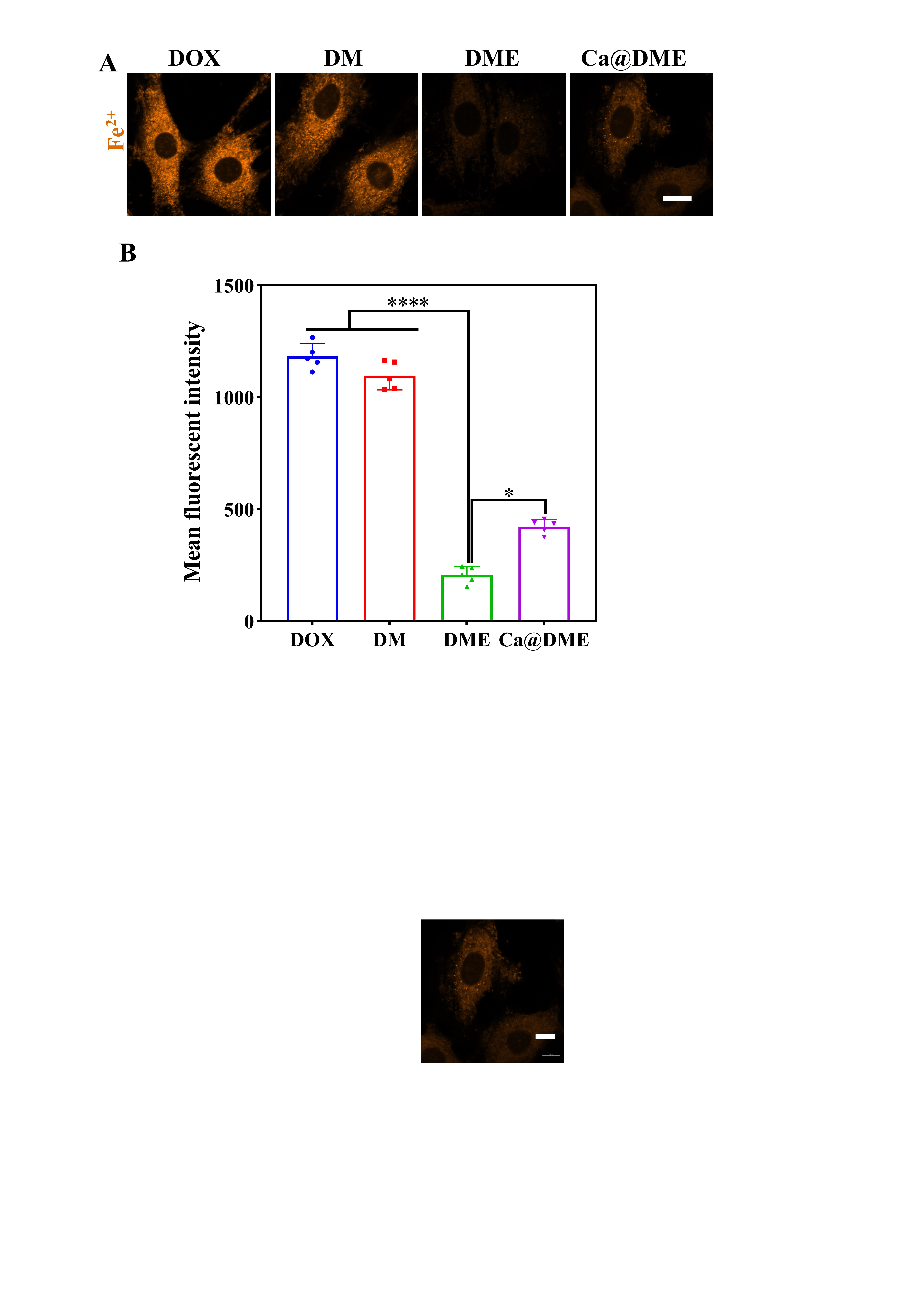


**Figure S33.** Detection of intracellular Fe^2+^ levels after different treatments (A) and the corresponding quantitative analysis (B). Scale bar = 15 µm, **p* < 0.05, *****p* < 0.0001.


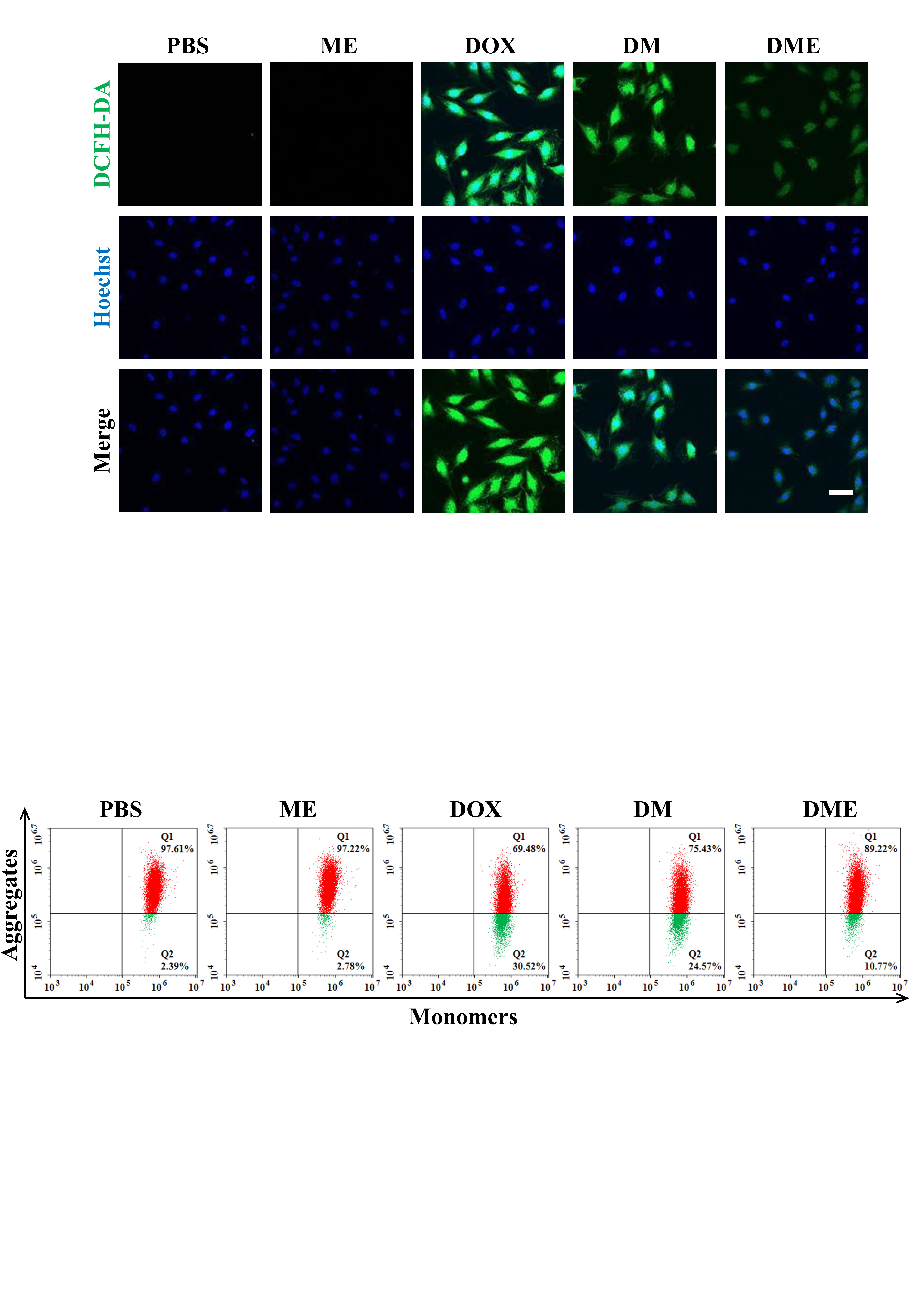


**Figure S34.** CLSM images stained with DCFH-DA (green) and Hoechst (blue) to detect ROS in cardiomyocytes after various treatments. Scale bar= 50 µm.


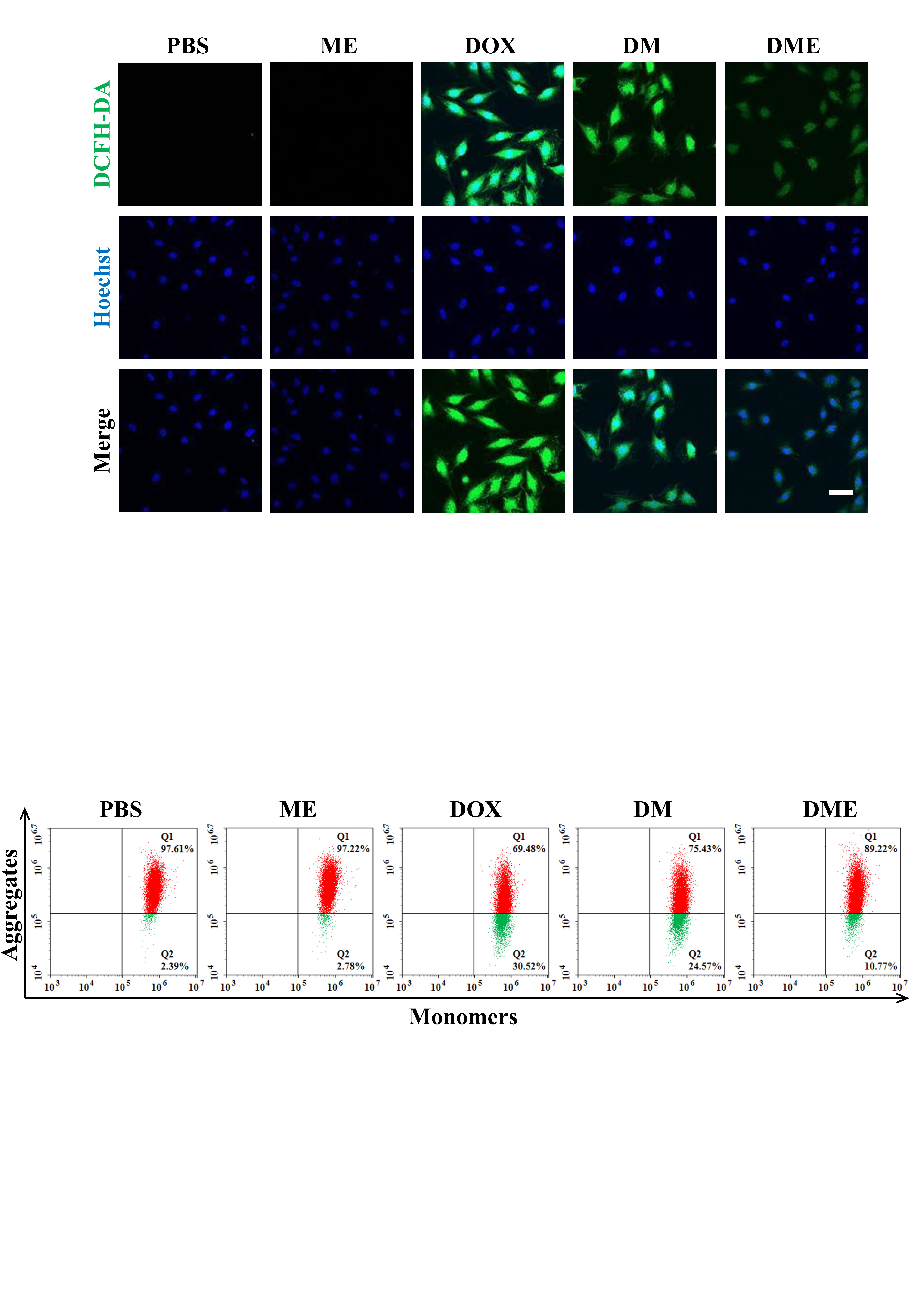


**Figure S35.** Quantitative analysis of mitochondrial membrane potential in cardiomyocytes after various treatments by flow cytometry.


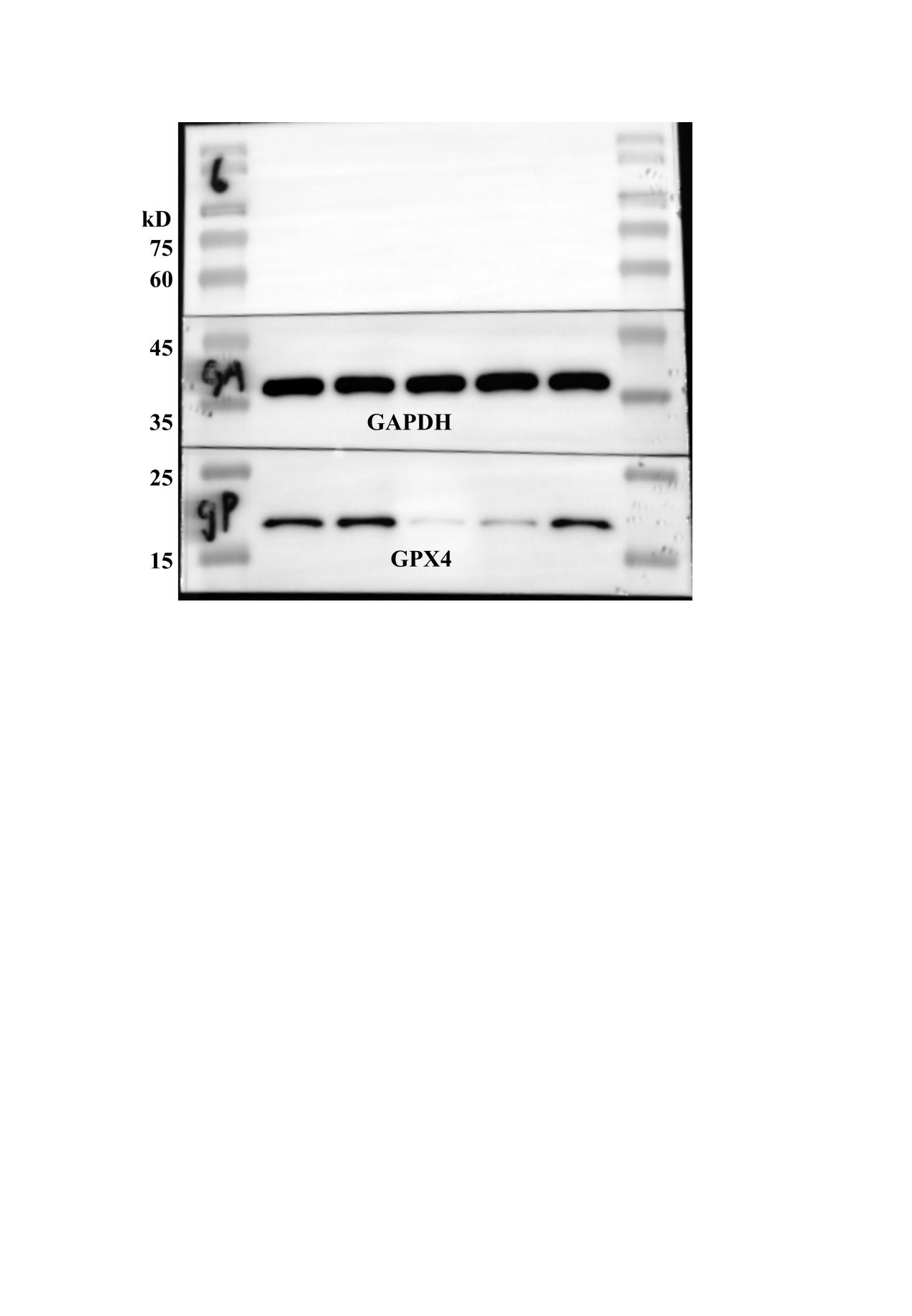


**Figure S36.** The full Western blot analysis of GPX4 protein in cardiomyocytes after various treatments.


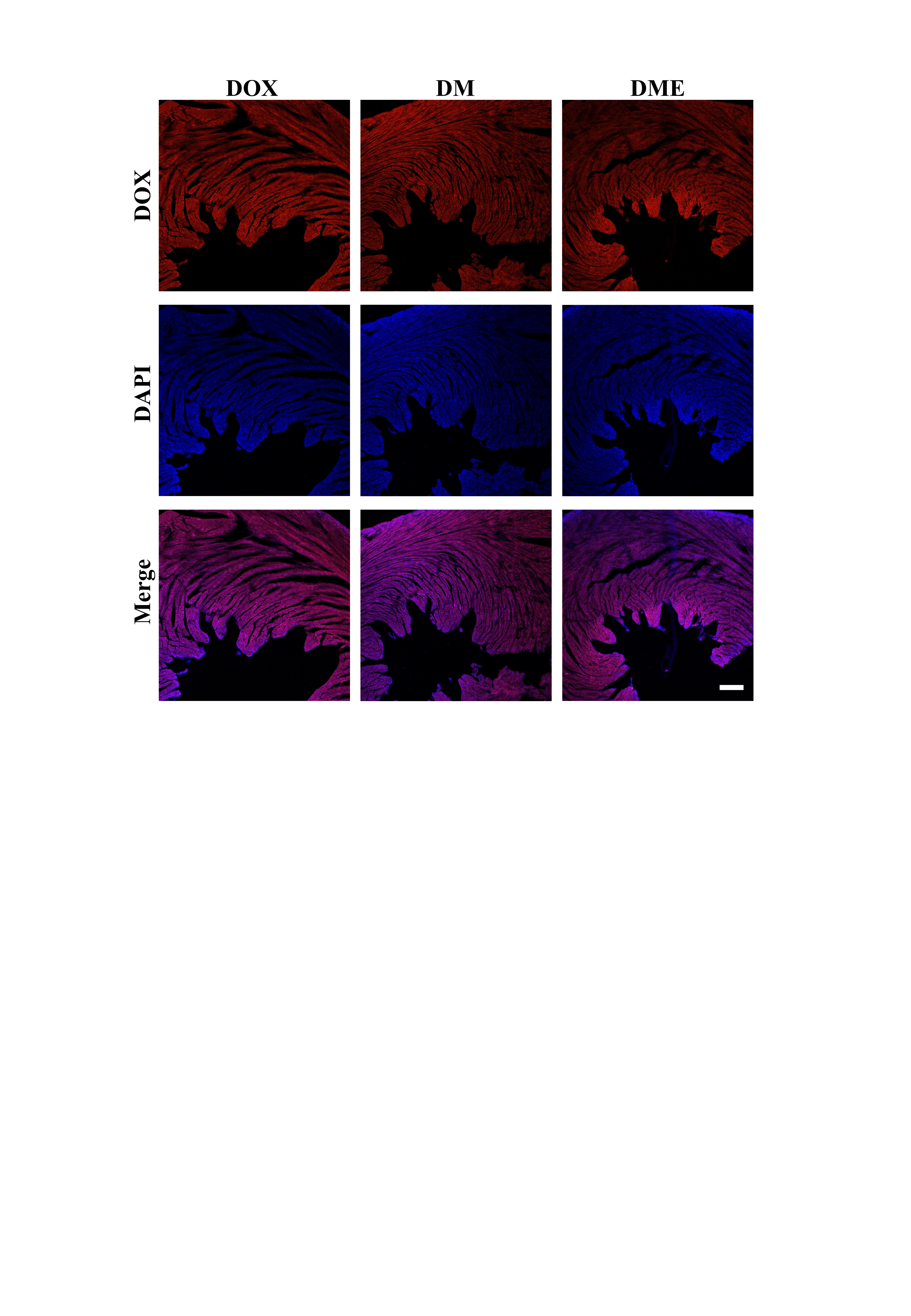


**Figure S37.** CLSM images of frozen section assay to detect DOX accumulation in cardiac tissues after various treatments. Scale bar = 250 µm.


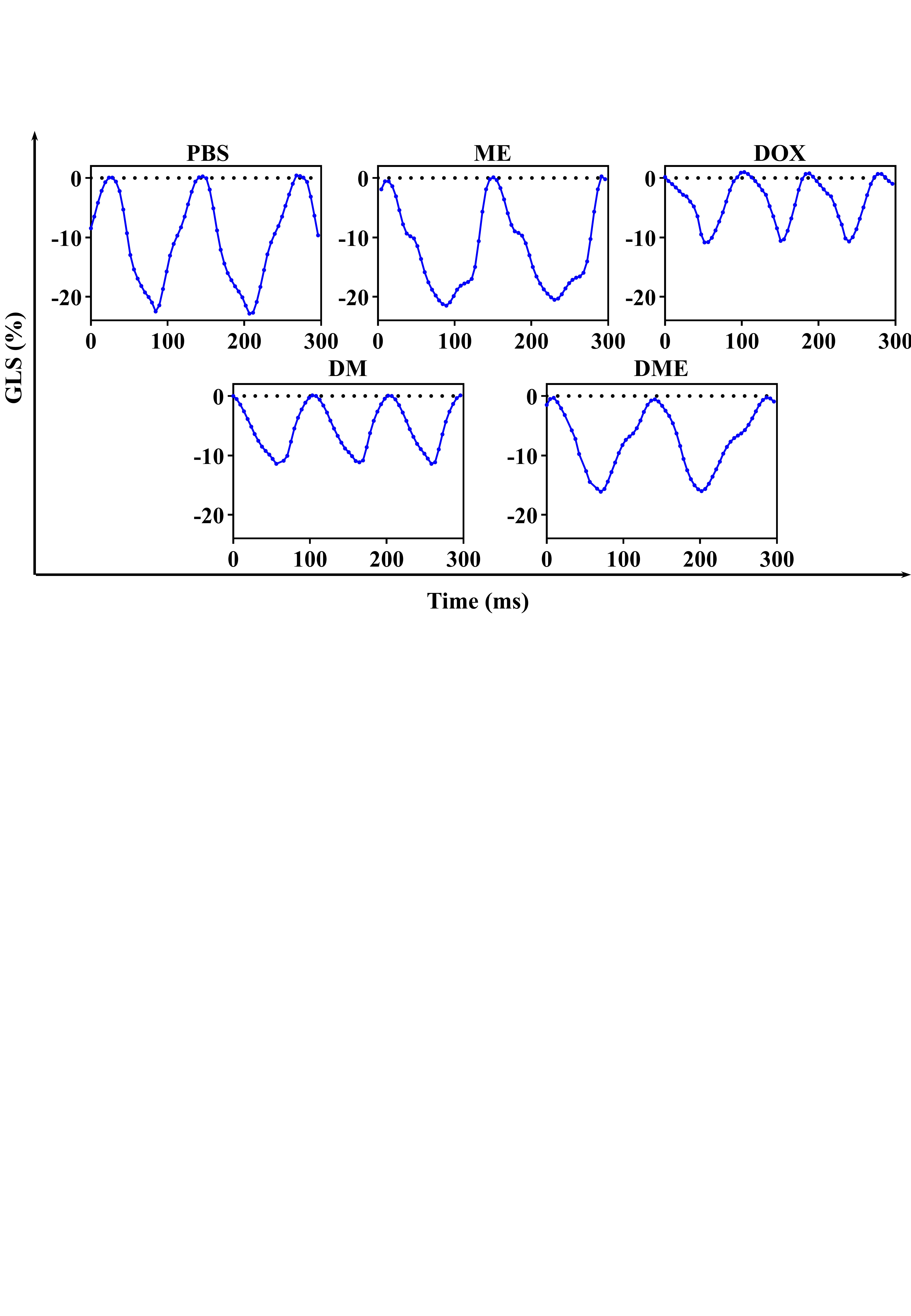


**Figure S38.** GLS-time curves of left ventricle after various treatments.


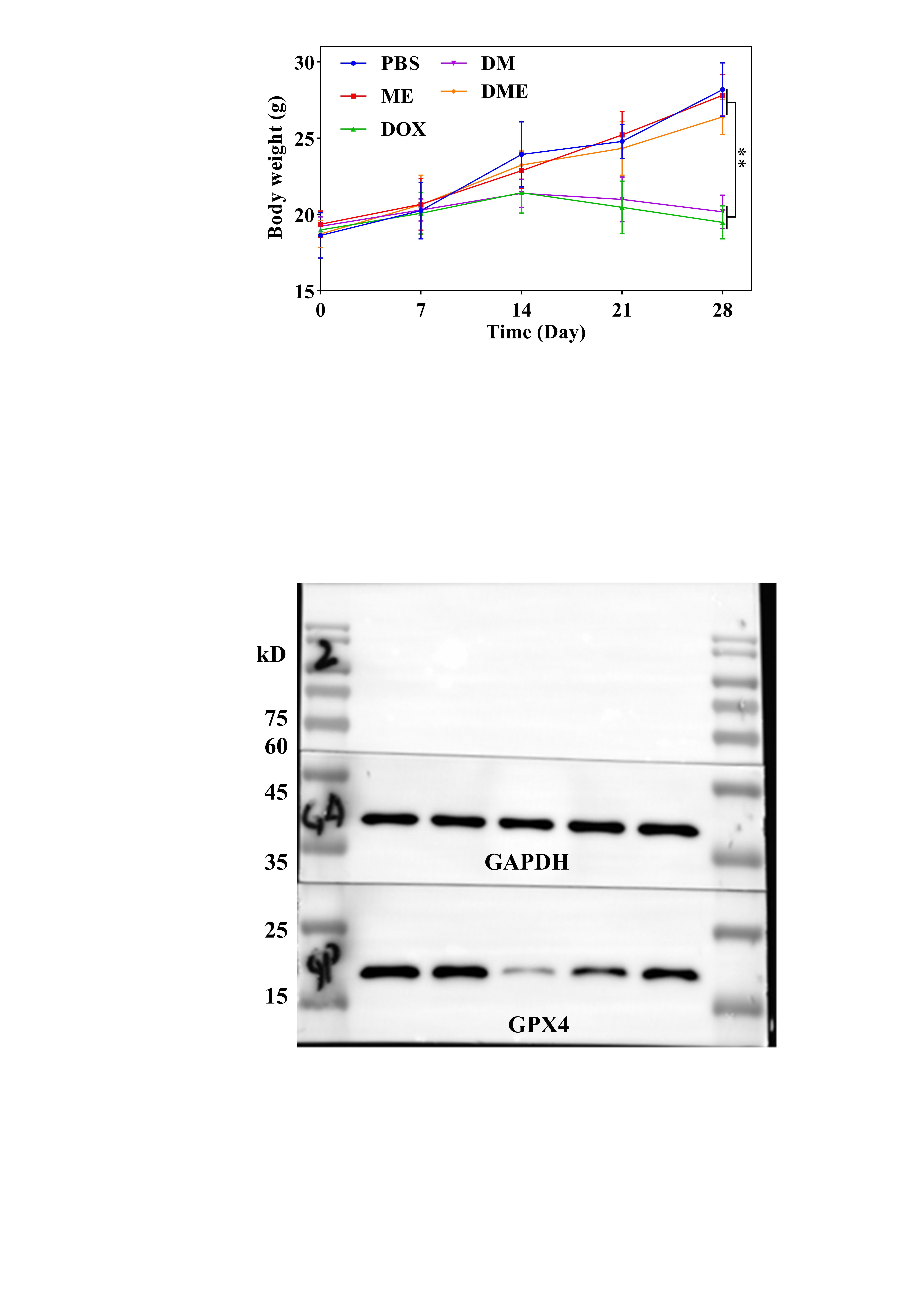


**Figure S39.** Body weight of mice during the treatment. ***p* < 0.01.


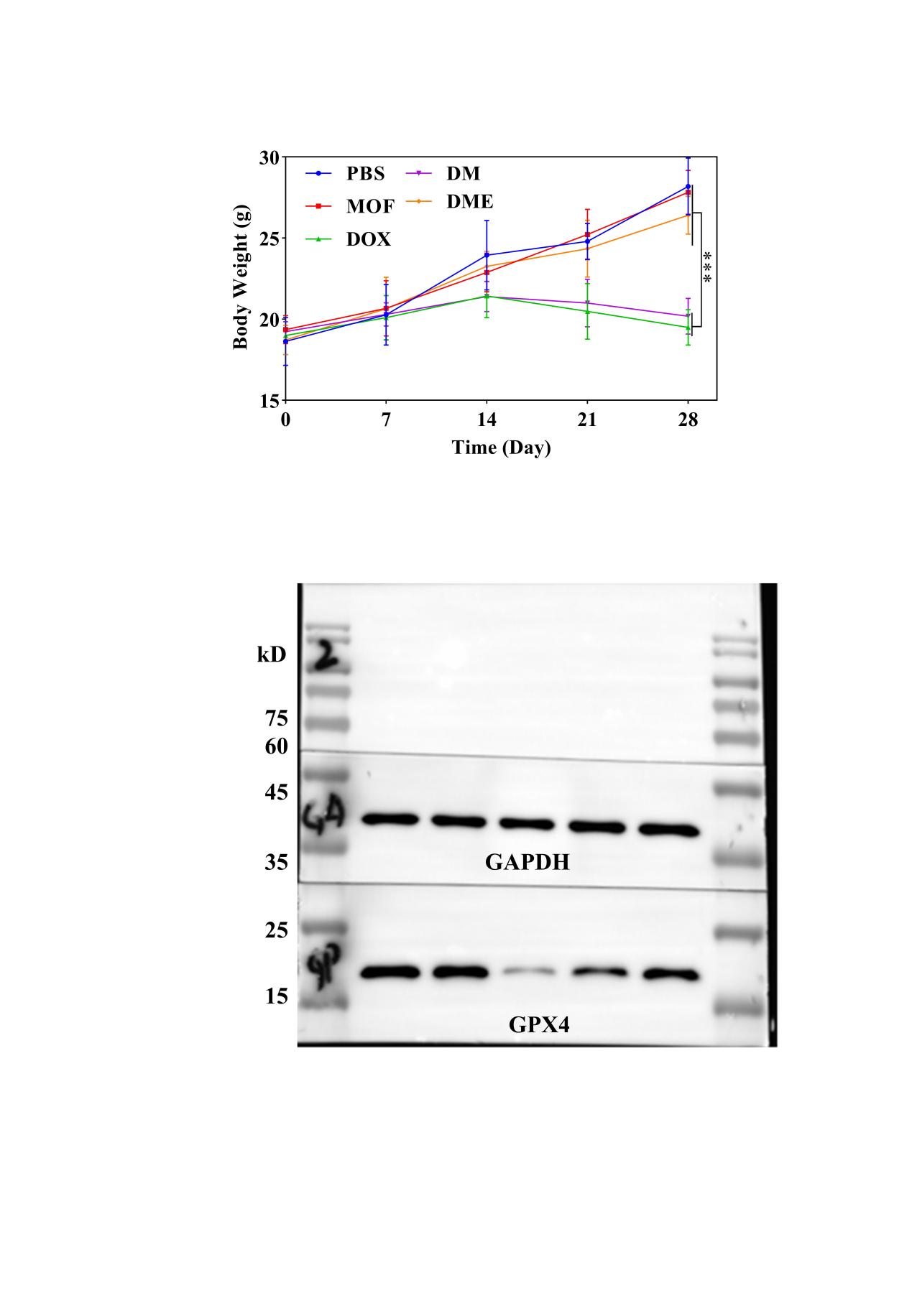


**Figure S40.** The full Western blot analysis of GPX4 protein in cardiac tissues after various treatments.


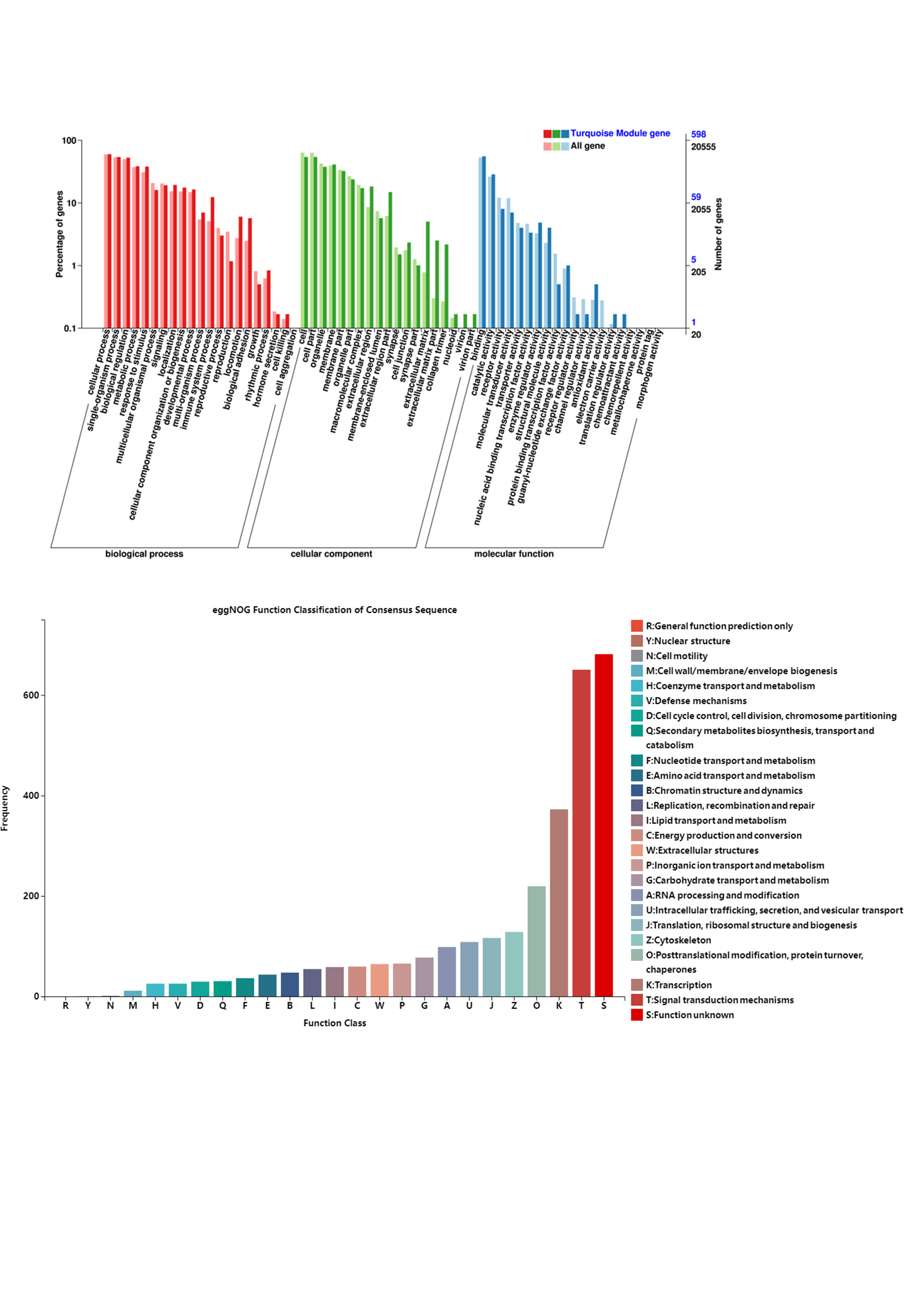


**Figure S41.** Gene Ontology (GO) enrichment analysis of differentially expressed genes (DEGs).


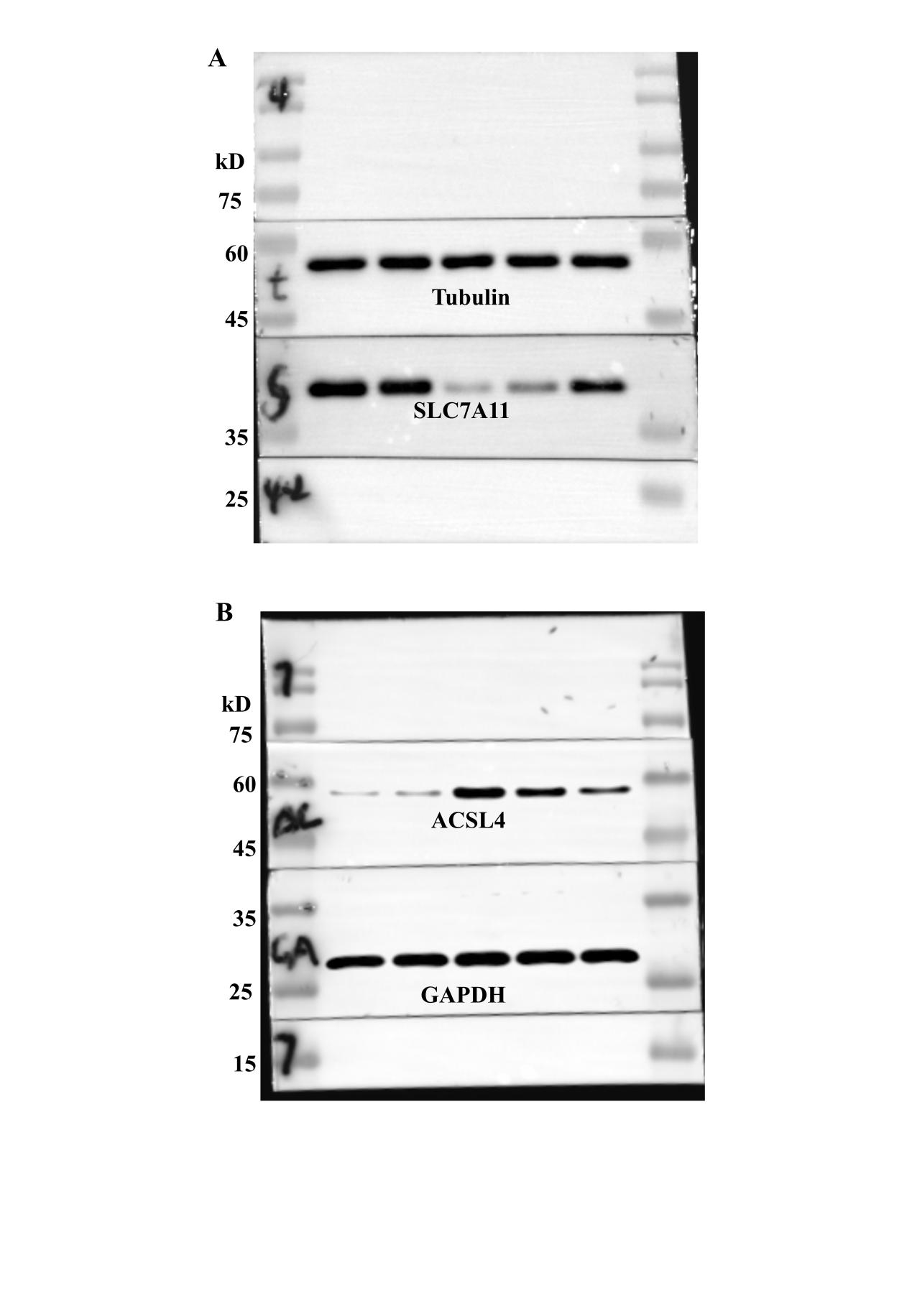


**Figure S42.** The full Western blot analysis of SLC7A11 (A) and ACSL4 (B) proteins in cardiomyocytes after various treatments.


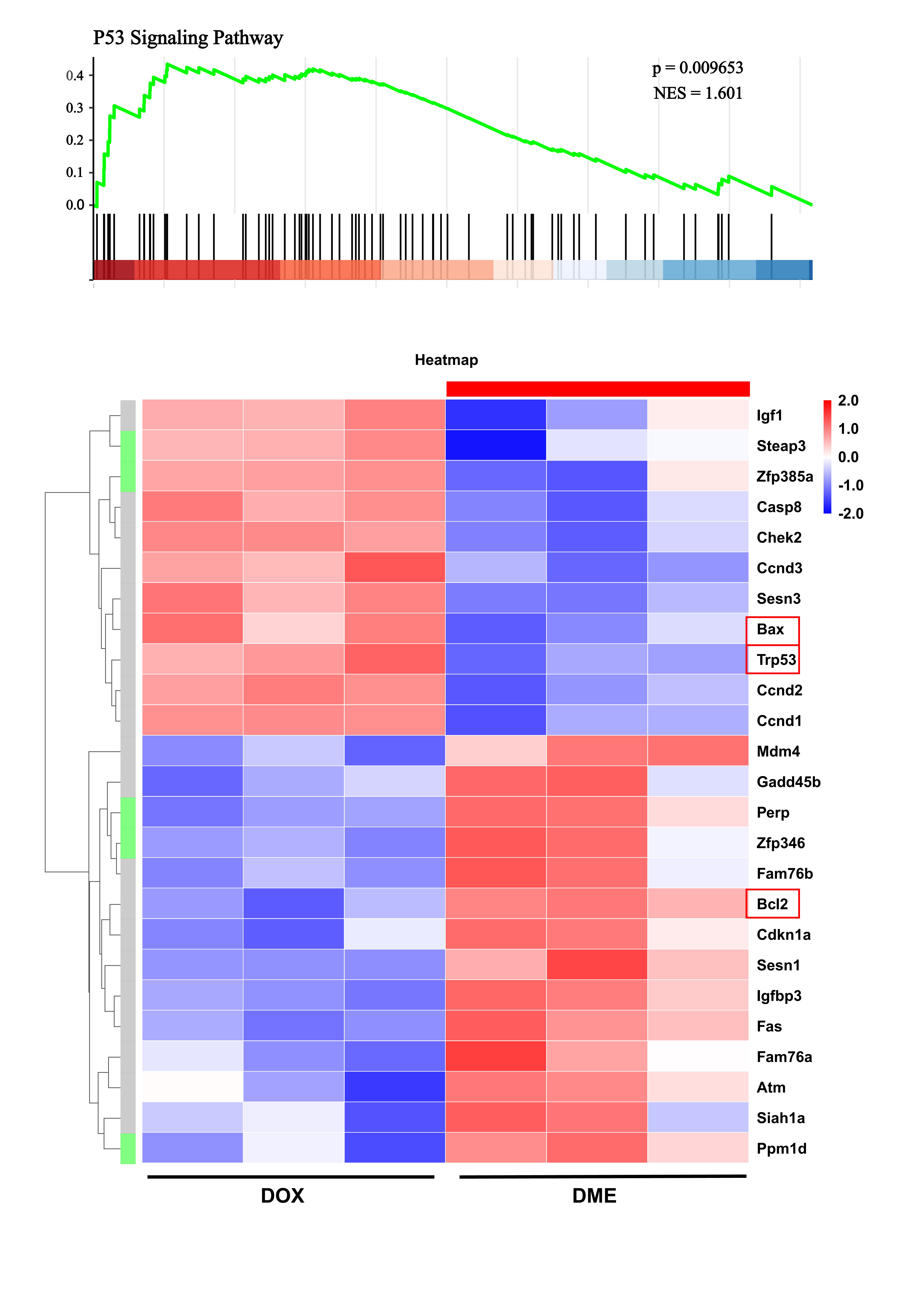


**Figure S43.** Gene Set Enrichment Analysis (GSEA) of P53 signaling pathway.


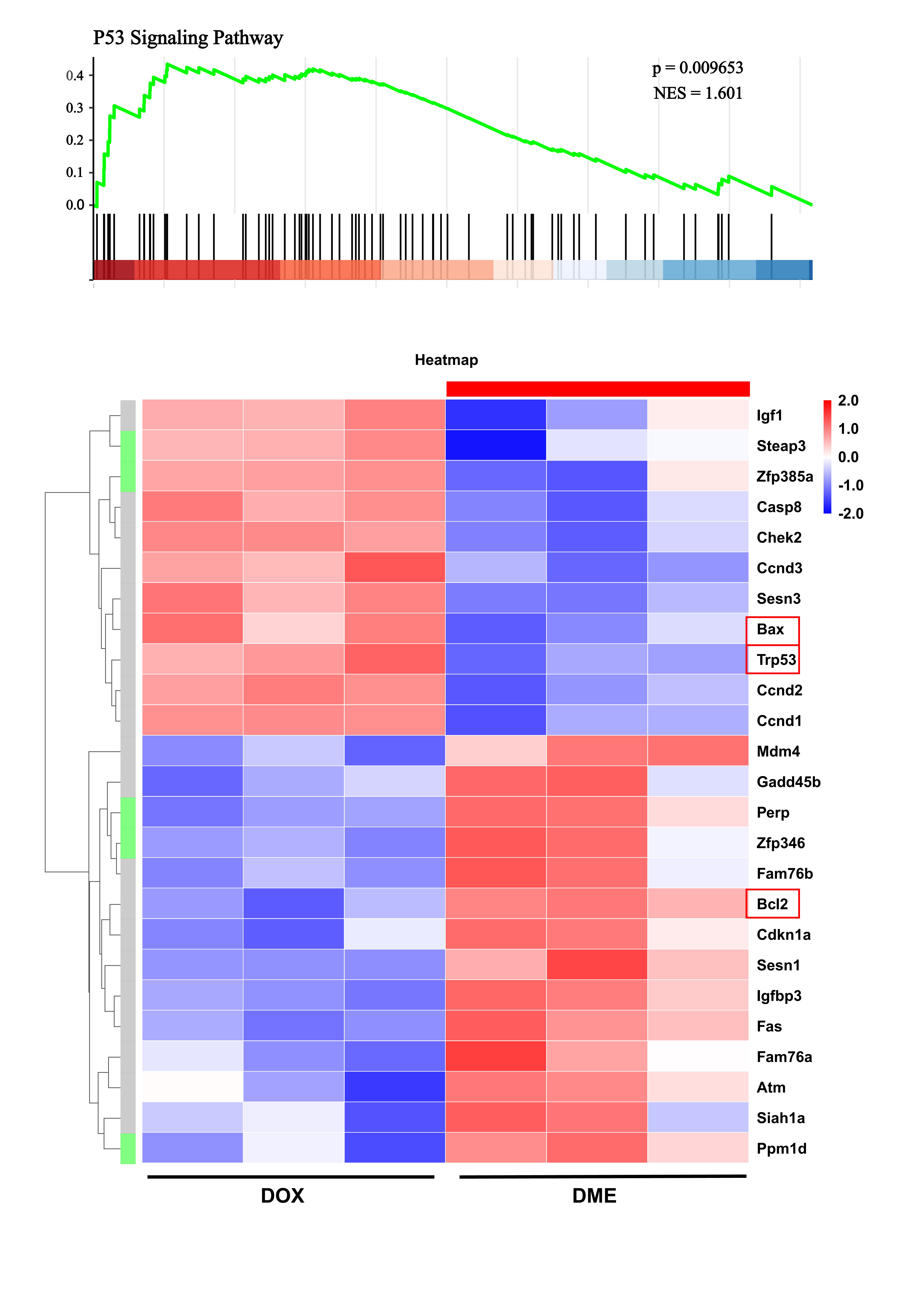


**Figure S44.** Heatmap of gene expressions in P53 signaling pathway.


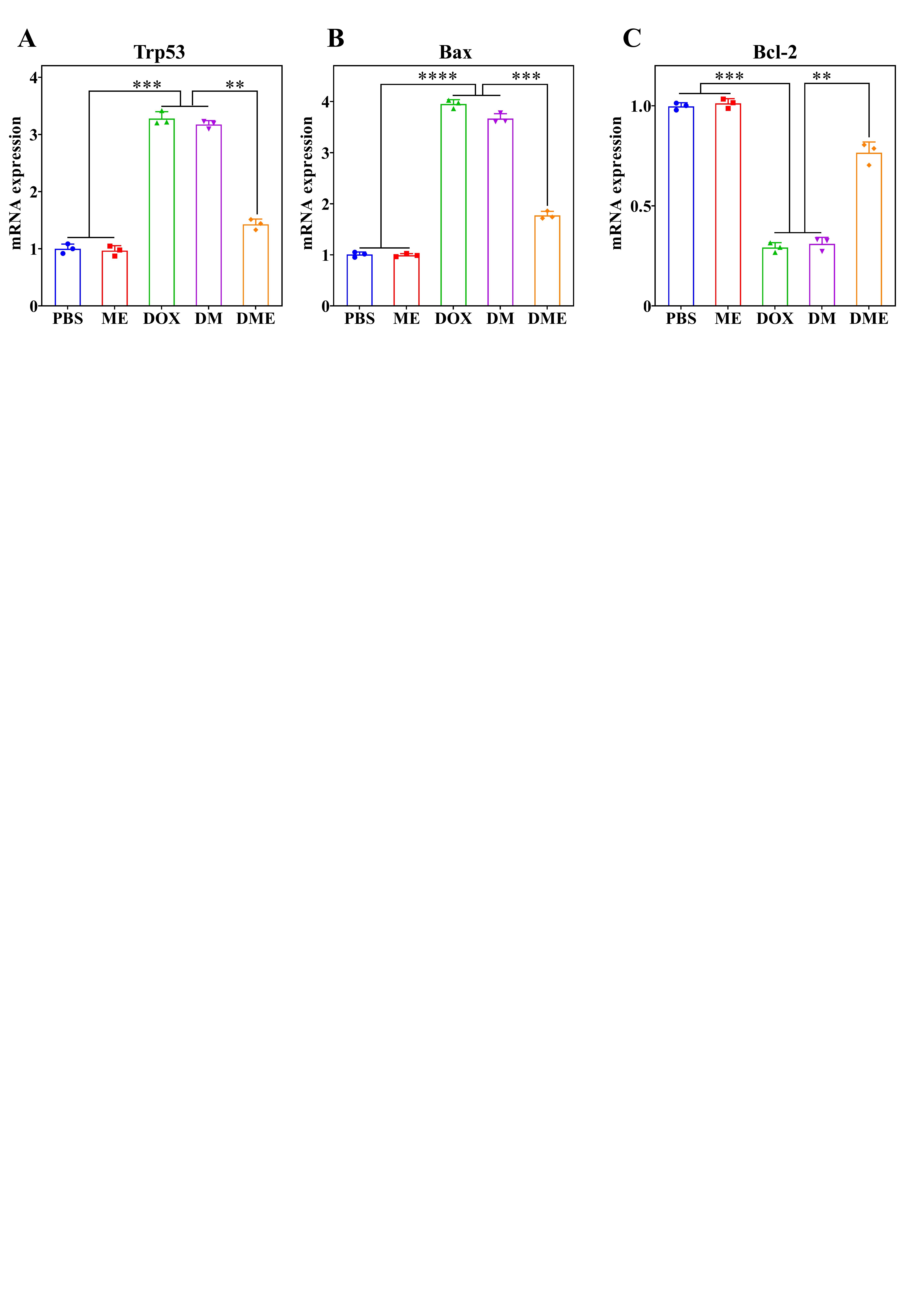


**Figure S45.** qPCR analysis of Trp53 (A), Bax (B) and Bcl-2 (C) mRNA expression in cardiomyocytes after various treatments. ***p* < 0.01, ****p* < 0.001, *****p* < 0.0001.


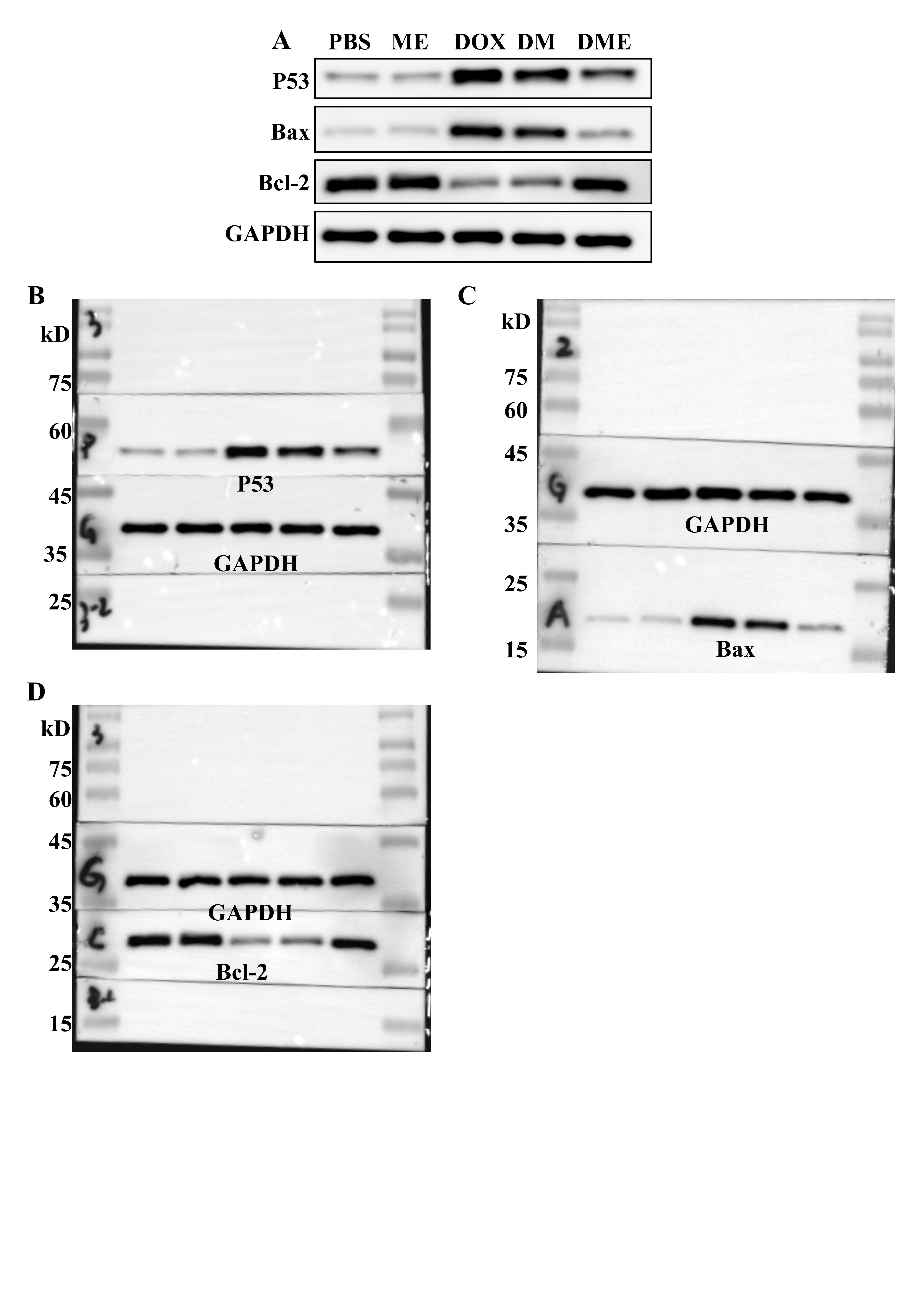


**Figure S46.** Western blot analysis of P53, Bax, and Bcl-2 proteins following various treatments in cardiomyocytes (A) and their full images (B-D).


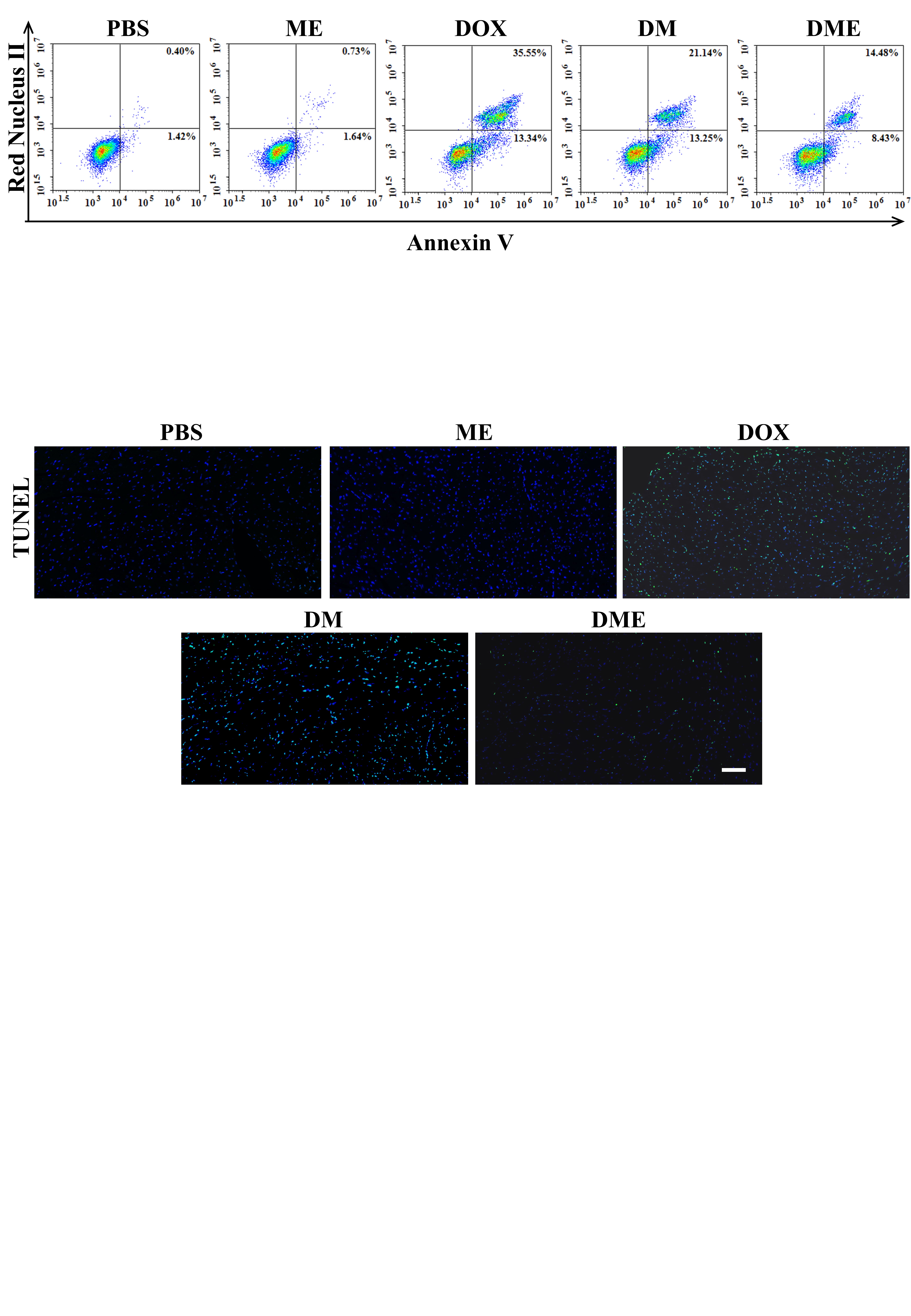


**Figure S47.** Flow cytometry stained with Annexin V/Red Nucleus Ⅱ to detect apoptosis of cardiomyocytes after various treatments.


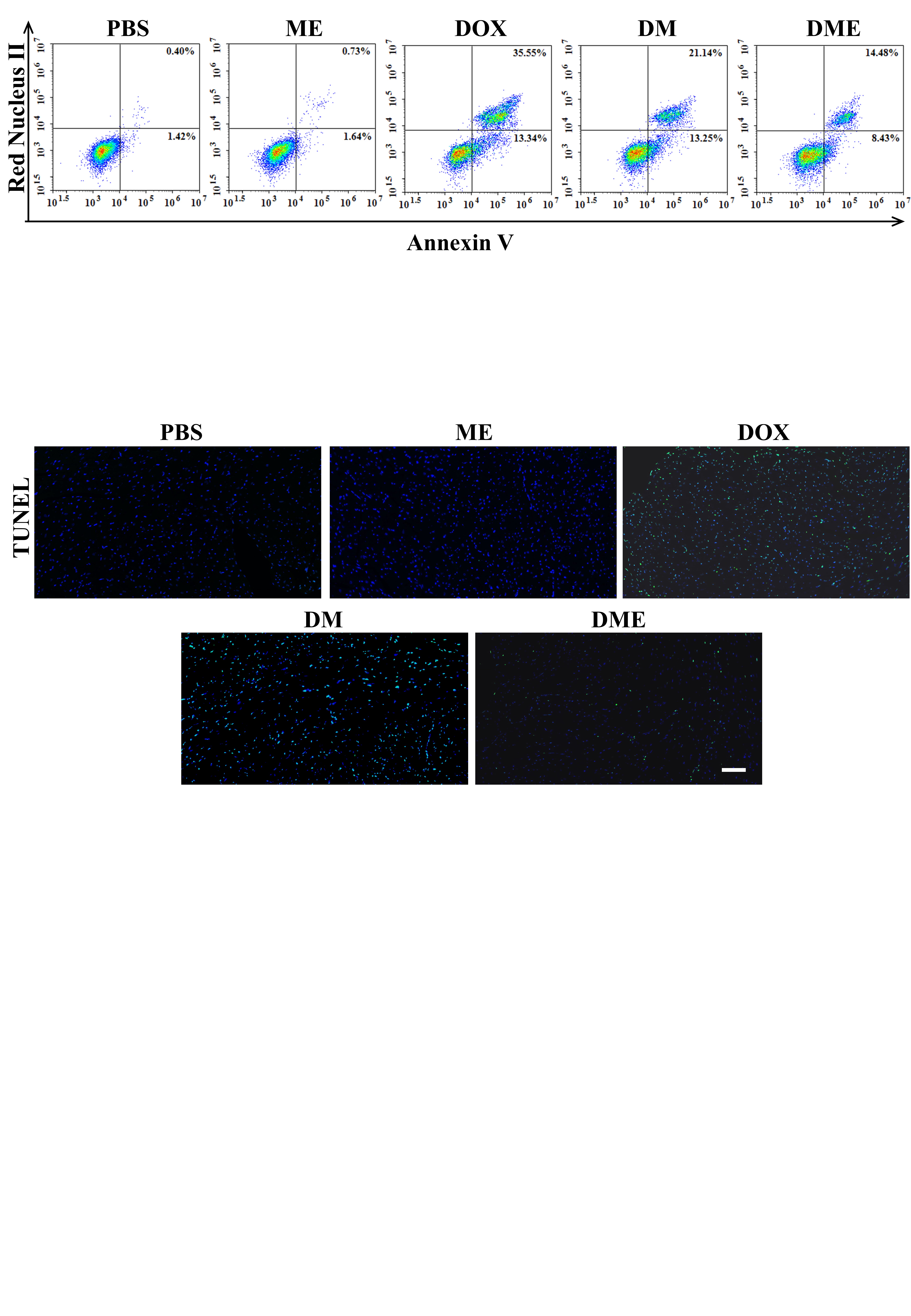


**Figure S48.** TUNEL staining images of cardiac tissues after different treatments. Scale bar = 50 µm.

**Figure S49.** The biodistribution of **DME** in different organs at various times determined by Hf^4+^ ions.


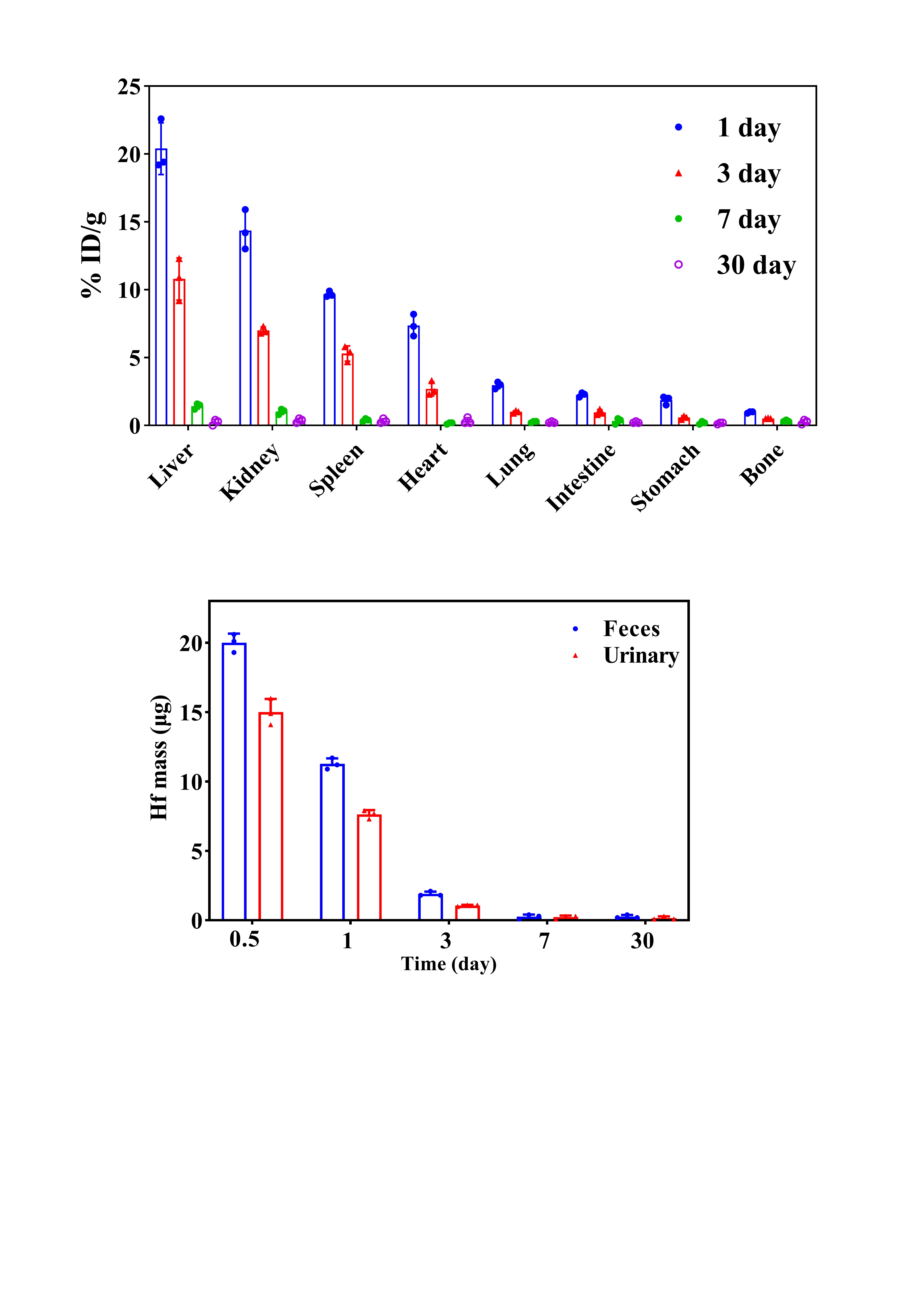


**Figure S50.** The content of Hf^4+^ ions in feces and urine at various times after the intravenous injection of **DME**.**Supplementary Table**

**Table S1.** Zeta potential of **ME, DM, DME** and **DMEC** nanoparticles in different solutions over time.


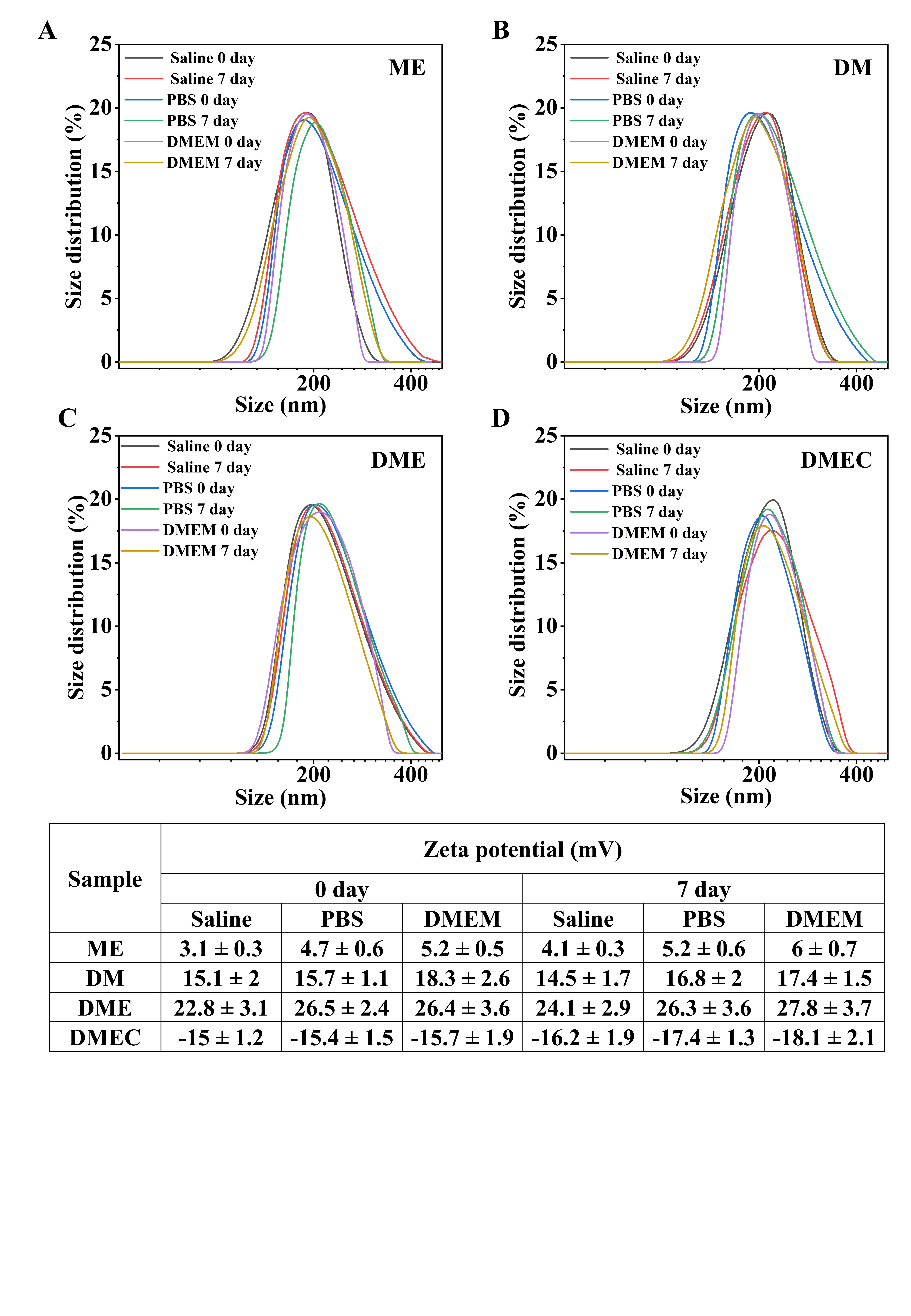


**Table S2.** Sensitization enhancement ratio (SER) values ascertained through colony formation assay after X-ray irradiation.


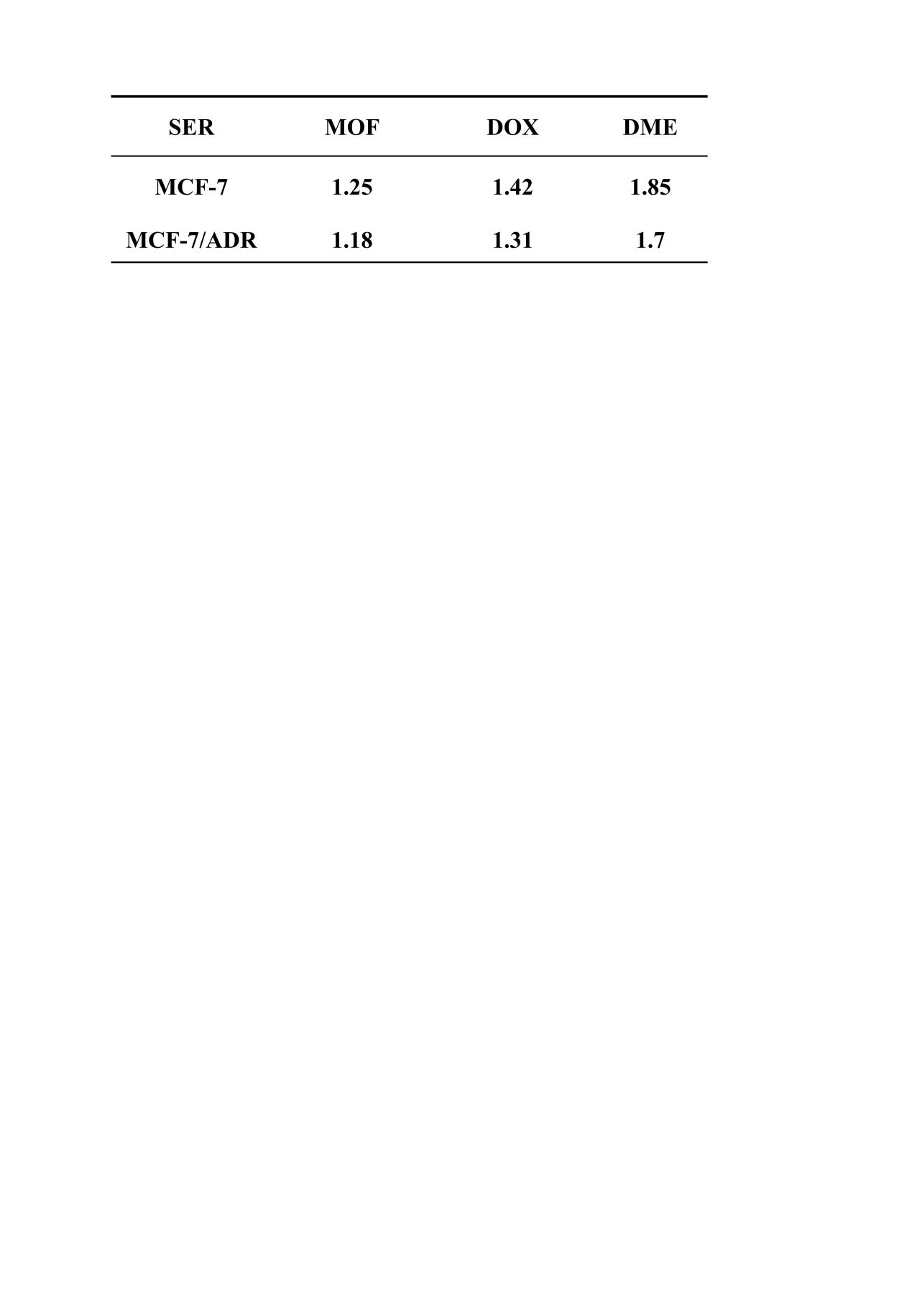


**Other Supplementary Materials for this manuscript include the following:**

**Movies S1 and S2**

**Movie S1:** Movie frame of M-mode echocardiography based on the left ventricle parasternal short-axis (PSAX) view.

**Movie S2:** Movie frame of dynamic speckle-tracking longitudinal strain based on the left ventricle parasternal long-axis (PLAX) view.**Supplementary Reference**

[1] N. Ma, Y. W. Jiang, X. Zhang, H. Wu, J. N. Myers, P. Liu, H. Jin, N. Gu, N. He, F. G. Wu, Z. Chen, *ACS Appl. Mater. Interfaces* **2016**, 8, 28480.
